# cGAS activation converges with intracellular acidification to promote STING aggregation and pyroptosis in tumor models

**DOI:** 10.1172/JCI188872

**Published:** 2025-07-15

**Authors:** Li Xiao, Yuan-li Ai, Xiang-yu Mi, Han Liang, Xiang Zhi, Liu-zheng Wu, Qi-tao Chen, Tong Gou, Chao Chen, Bo Zhou, Wen-bin Hong, Lu-ming Yao, Jun-jie Chen, Xianming Deng, Fu-nan Li, Qiao Wu, Hang-zi Chen

**Affiliations:** 1 The First Affiliated Hospital of Xiamen University, State Key Laboratory of Cellular Stress Biology, School of Life Sciences, Xiamen University, Xiamen, Fujian, China.; 2 Fujian Provincial Key Laboratory of Innovative Drug Target Research, School of Pharmaceutical Sciences, Xiamen University, Xiamen, China.

**Keywords:** Cell biology, Oncology, Cancer, Mitochondria

## Abstract

The cyclic GMP-AMP synthase (cGAS)/stimulator of IFN genes (STING) pathway is intimately associated with antitumoral immunity; however, the direct involvement of this pathway in tumor cell demise remains elusive. Here, we identified a compound, dodecyl 6-hydroxy-2-naphthoate (DHN), that induces pyroptosis in melanoma cells by activating noncanonical cGAS/STING signaling. DHN targets mitochondrial protein cyclophilin D (CypD) to induce the release of mitochondrial DNA, leading to cGAS activation and cyclic GMP-AMP (cGAMP) generation. Meanwhile, DHN-caused intracellular acidification induces protein kinase R-like endoplasmic reticulum kinase (PERK) activation, which promotes STING phosphorylation and polymerization in the presence of cGAMP, thereby facilitating the aggregation of STING in the ER, which serves as a platform to recruit Fas-associated via death domain (FADD) and caspase-8, leading to caspase-8 activation and subsequent gasdermin E cleavage, which ultimately results in pyroptosis of tumor cells and tumor regression in mouse models. The occurrence of this noncanonical cGAS/STING pathway–associated pyroptosis is also observed when both cGAS is activated and intracellular pH declines. Collectively, our findings reveal a pathway that links noncanonical cGAS/STING signaling to gasdermin E–mediated pyroptosis, thereby offering valuable insights for tumor therapy.

## Abstract 2

Non-canonical activation of the cGAS-STING signaling pathway by the small molecular compound DHN, resulting in ER-dependent aggregation of STING and subsequent GSDME-mediated pyroptosis.

## Graphical abstract

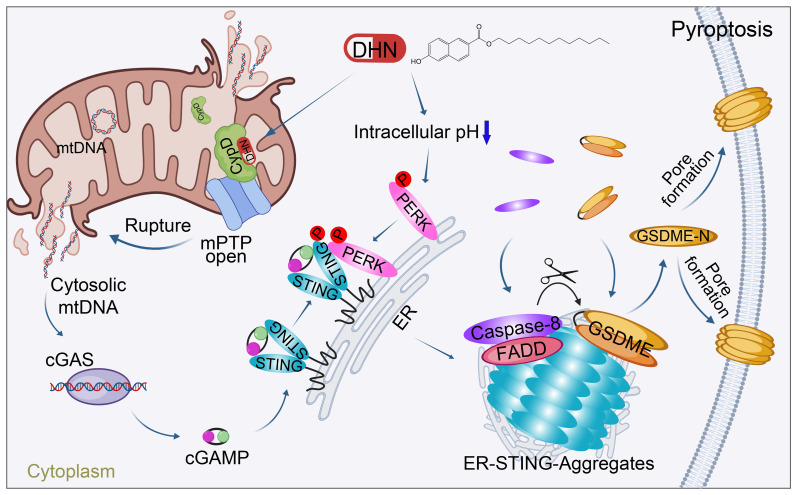

## Introduction

Pyroptosis, a recently discovered form of regulated cell death, is mediated by members of the gasdermin (GSDM) family that undergo cleavage to generate the N-terminal domain responsible for cell membrane perforation (1). Several proteases have been identified for GSDM cleavage, with caspases being prominent examples. For instance, activation of caspase-8 by metabolite α-ketoglutarate or TNF-α leads to GSDMC cleavage and subsequent induction of pyroptosis in tumor cells (2, 3). Caspase-1/4/5/11 are known to cleave GSDMD during Gram-negative bacterial infections in many cases (4, 5). However, upon *Yersinia* infection, caspase-8 is implicated in GSDMD cleavage. In many studies, caspase-3 has been reported to cleave GSDME after stimulation with a chemotherapy drug (6–8), and coculture with killer lymphocytes induces granzyme B–dependent GSDME cleavage (9). These studies underscore the significance of elucidating additional regulatory mechanisms governing protease-mediated GSDM cleavage under different circumstances.

As a cytoplasmic DNA sensor, cyclic GMP-AMP synthase (cGAS) recognizes not only foreign microbial (viral and bacterial) DNA in the cytoplasm but also its own DNA. Upon activation by cytosolic DNA, cGAS catalyzes ATP and GTP to generate cGAMP, which binds to and activates stimulator of IFN genes (STING). Activated STING translocates from the ER to the Golgi apparatus, where it undergoes polymerization. The polymerized STING recruits TBK1 to cause its autoactivation, thereby facilitating IRF3 phosphorylation. Phosphorylated IRF3 then translocates into the nucleus to initiate IFN transcriptional expression (10). In tumor cells, cytoplasmic DNA accumulation arises due to defects in the DNA damage response, chromosomal instability, replicative stress, reactivation of endogenous retroelements, or release of mitochondrial DNA (mtDNA). These events activate the cGAS/STING signaling pathway, leading to upregulation of type I IFNs, proinflammatory cytokines, and chemokines (11), thereby playing a crucial role in antitumor immunity.

In addition to its canonical proinflammatory effect, STING is also involved in the induction of regulatory cell death through a noncanonical pathway. Upon activation, STING localizes to the lysosome and triggers membrane permeabilization, leading to lysosomal cell death in human myeloid cells (12). In neuronal cells, glutamate excitotoxicity activates STING, resulting in autophagic degradation of glutathione peroxidase 4, which is essential for neuronal redox homeostasis and consequently induction of ferroptosis (13). STING activation has been reported to mediate PANoptosis in diffuse large B cell lymphoma cells (14). However, the role of STING in pyroptosis remains controversial. On the one hand, oxidative stress–induced cGAS/STING activation stimulates NLRP3 inflammasome–mediated pyroptosis in human nucleus pulposus cells (15); on the other hand, depletion of STING in renal cell carcinoma leads to caspase-8 activation and GSDMD-mediated pyroptosis (16). Yet, the interplay between cGAS/STING signaling and GSDME-dependent pyroptosis has not been elucidated.

Protein kinase R-like endoplasmic reticulum kinase (PERK) is a key sensor of the unfolded protein response in the ER. Upon activation, PERK inhibits protein synthesis, maintains cellular oxidative homeostasis, and enhances ER quality control (17). Recently, the identification of a noncanonical cGAS/STING/PERK pathway has emerged as pivotal in regulating senescence and organ fibrosis through modulation of the translational program (18). However, whether PERK participates in pyroptosis induction through regulation of the cGAS/STING pathway remains unknown. Herein, we elucidate a mechanism whereby pyroptosis is induced by noncanonical STING signaling regulated by PERK. A small molecule compound, dodecyl 6-hydroxy-2-naphthoate (DHN), from our in-house library was identified to have the capability of binding to mitochondrial protein cyclophilin D (CypD), leading to mitochondrial permeability transition pore (mPTP) opening and subsequent release of mtDNA to the cytoplasm. This process activates cGAS and results in cGAMP production. Additionally, DHN-induced intracellular acidification activates PERK that interacts with and phosphorylates STING in the presence of cGAMP. This STING phosphorylation promotes its oligomerization and subsequent aggregation in the ER, thereby providing a platform for recruitment and cleavage of GSDME by caspase-8, ultimately inducing pyroptosis. Overall, this study reveals the function and mechanism involved in the formation of STING-dependent aggregates for pyroptotic induction.

## Results

### Compound DHN induces pyroptosis through caspase-8–mediated GSDME cleavage.

**Figure 1:**
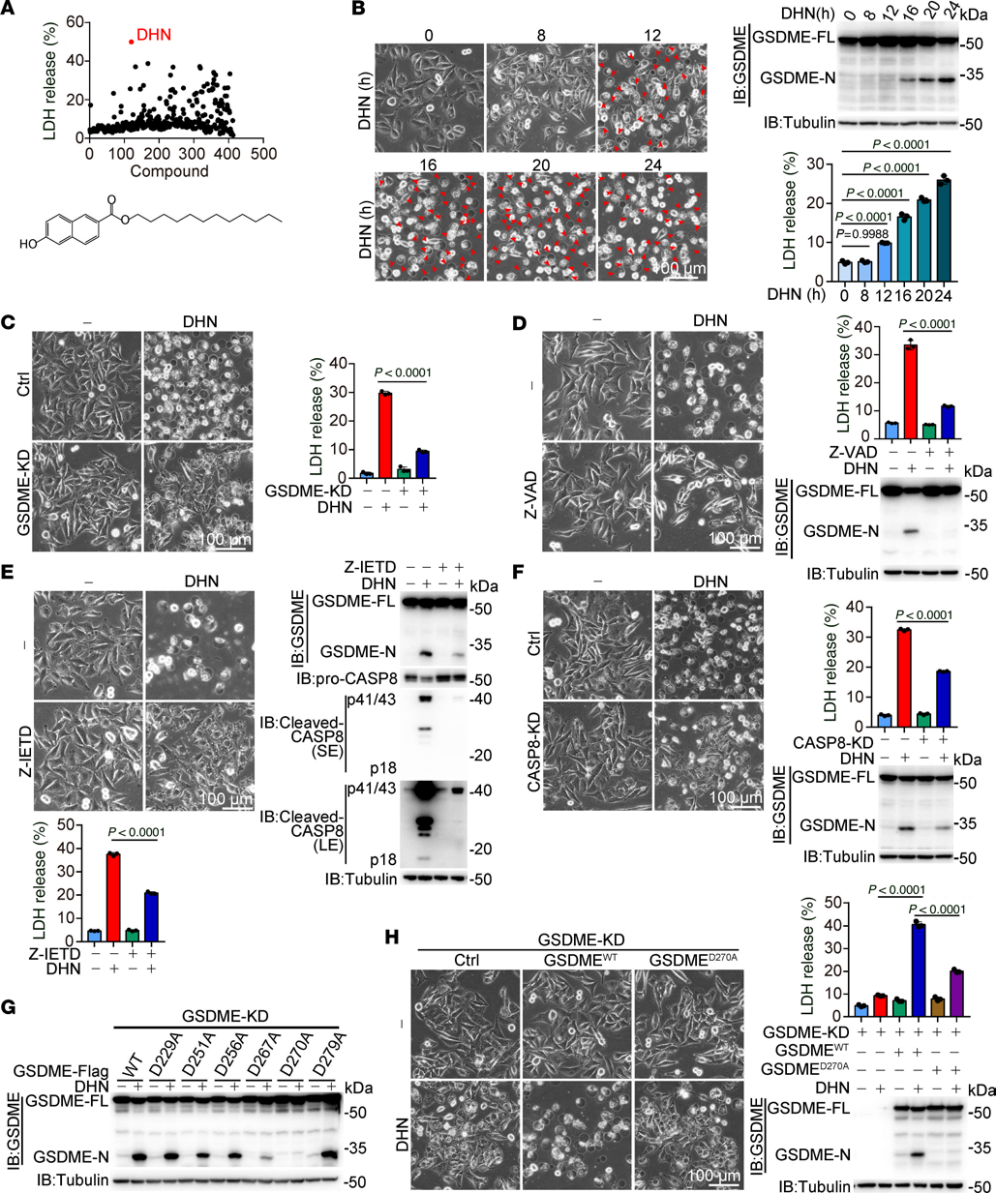
DHN induces pyroptosis by caspase-8–mediated cleavage of GSDME. Melanoma A375 cells were treated with DHN (15 μM) for 20 hours to assess pyroptotic features (including characteristic morphology, GSDME cleavage, and LDH release), unless specifically defined. (**A**) Compound library screening and chemical structure of DHN. (**B**) DHN-induced pyroptosis at different time points and cells with characteristic pyroptotic morphology indicated by red arrows. Cleavage of GSDME was detected by Western blot, and cell death was evaluated by accessing LDH release. (**C**–**F**) GSDME (**C**) or caspase-8 (**F**) were separately knocked down in cells or cells were cotreated with Z-VAD (**D**, 20 μM) or Z-IETD (**E**, 10 μM), followed by detection of pyroptosis. (**G**) GSDME^WT^, GSDME^D229A^, GSDME^D251A^, GSDME^D256A^, GSDME^D267A^, GSDME^D270A^, or GSDME^D279A^ were separately transfected into GSDME-knockdown cells, then the cleavage level of GSDME was detected. (**H**) GSDME^WT^ or GSDME^D270A^ were separately transfected into GSDME-knockdown cells, then pyroptosis was detected. Tubulin was used to determine the amount of loading protein. Data are presented as mean ± SEM of 3 independent experiments. Statistical analyses were determined by 1-way ANOVA with Tukey’s multiple-comparison test (**B**) and 2-way ANOVA with Tukey’s multiple-comparison test (**C**–**F** and **H**). *P* values are indicated in figures. Scale bars: 100 μm. All Western blots were repeated at least twice, and 1 of them is shown. IB, immunoblot; LE, long exposure; SE, short exposure.

Considering the inherent resistance of melanoma cells to apoptosis, lytic cell death may offer a more effective therapeutic approach by activating antitumoral immunity. To identify agents capable of inducing lytic cell death, we screened our in-house compound library using the lactate dehydrogenase (LDH) release assay, a well-established method for quantifying lytic cell death. This proprietary library was collaboratively developed by our research team and partners, and primarily consists of derivatives of Csn-B and THPN, compounds known to induce apoptosis and autophagic cell death, respectively (19, 20). Our results demonstrated that a compound named DHN was the most potent compound for inducing lytic cell death in A375 melanoma cells (Figure 1A). Further morphological assessment after DHN treatment revealed characteristic pyroptotic features, including cell swelling and the formation of large bubbles from the plasma membrane, as indicated by red arrowheads in Figure 1B. This DHN-induced characteristic pyroptotic morphology was closely associated with LDH release and cleavage of the pyroptotic executor GSDME in melanoma A375 cells and other cancer cell lines (Figure 1B and Supplemental Figure 1A; supplemental material available online with this article; https://doi.org/10.1172/JCI188872DS1). Knockdown of GSDME, but not other GSDM proteins, in A375 cells attenuated DHN-induced pyroptosis (Figure 1C and Supplemental Figure 1, B and C), demonstrating the involvement of GSDME-mediated pyroptosis. Notably, no DNA laddering or annexin V^+^/propidium iodide^–^ cells, which are typical apoptotic markers, were observed upon DHN stimulation (Supplemental Figure 1D). Furthermore, pretreatment with Lip-1, Fer-1 (ferroptosis inhibitors), Necrosulfonamide, Nec-1 (necroptosis inhibitors), or Tetrathiomolybdate (a cuproptosis inhibitor) did not affect DHN-induced LDH release or pyroptotic morphology (Supplemental Figure 1E). Additionally, we investigated whether DHN could induce pyroptosis in nontumor cells and found that DHN exhibited a diminished capacity for pyroptotic induction in nontumor cells, including HK-2 human kidney proximal tubule epithelial cells, AC16 human cardiomyocytes, HEK293T cells, THP-1 human leukemia monocytic cells, HL-1 mouse cardiomyocytes, L929 mouse fibroblasts, and primary mouse BM-derived macrophages and BM-derived DCs, as compared with A375 melanoma cells (Supplemental Figure 1F). Collectively, these findings indicate that DHN predominantly induced GSDME-dependent pyroptosis rather than apoptosis, ferroptosis, necroptosis, or cuproptosis in tumor cells.

Cotreatment with Z-VAD, a pan-caspase inhibitor, abrogated DHN-induced pyroptosis (Figure 1D), suggesting the participation of a caspase protein in GSDME cleavage. In accordance with previous reports, incubation of immunoprecipitated GSDME protein with recombinant caspase-3 resulted in clear cleavage of GSDME in the in vitro assay (6, 7). However, we unexpectedly found that knockdown of caspase-3 in cells had no impact on DHN-induced pyroptosis (Supplemental Figure 1G), excluding the possibility of caspase-3–mediated GSDME cleavage upon stimulation with DHN. The treatment with DHN was found to markedly induce caspase-8 activation while only minimally activating caspase-3 (Supplemental Figure 1H). Cotreatment with Z-IETD, a specific inhibitor of caspase-8, or knockdown of caspase-8 effectively attenuated DHN-induced pyroptosis in A375 cells (Figure 1, E and F, and Supplemental Figure 1B), suggesting the involvement of caspase-8 in cleaving GSDME for pyroptotic induction. Although recombinant caspase-8 exhibited mild cleavage of GSDME under normal in vitro conditions, the appearance of the cleaved band of GSDME migrating at approximately 30–35 kDa was markedly enhanced as the pH value decreased to 6.5 in the in vitro cleavage buffer (Supplemental Figure 1I), implying that caspase-8 is capable of cleaving GSDME directly within an acidic environment.

Given that caspase-8 cleaves its substrate after an Asp residue (21), different Asp residues around the hinge region of GSDME were mutated into Ala. The in vitro results showed that GSDME^D270A^ completely blocked caspase-8–mediated cleavage (Supplemental Figure 1J), suggesting that caspase-8 may target GSDME Asp270 for cleavage, similar to caspase-3 (6, 7). When different GSDME mutants were separately expressed in the GSDME-knockdown A375 cells, only GSDME^D270A^ completely blocked DHN-induced GSDME cleavage (Figure 1G), thereby attenuating pyroptotic induction (Figure 1H). Taken together, these results demonstrated that DHN serves as a compound capable of inducing pyroptosis through caspase-8–mediated cleavage of GSDME.

### DHN targets mitochondrial protein CypD to promote the opening of mPTP.

**Figure 2:**
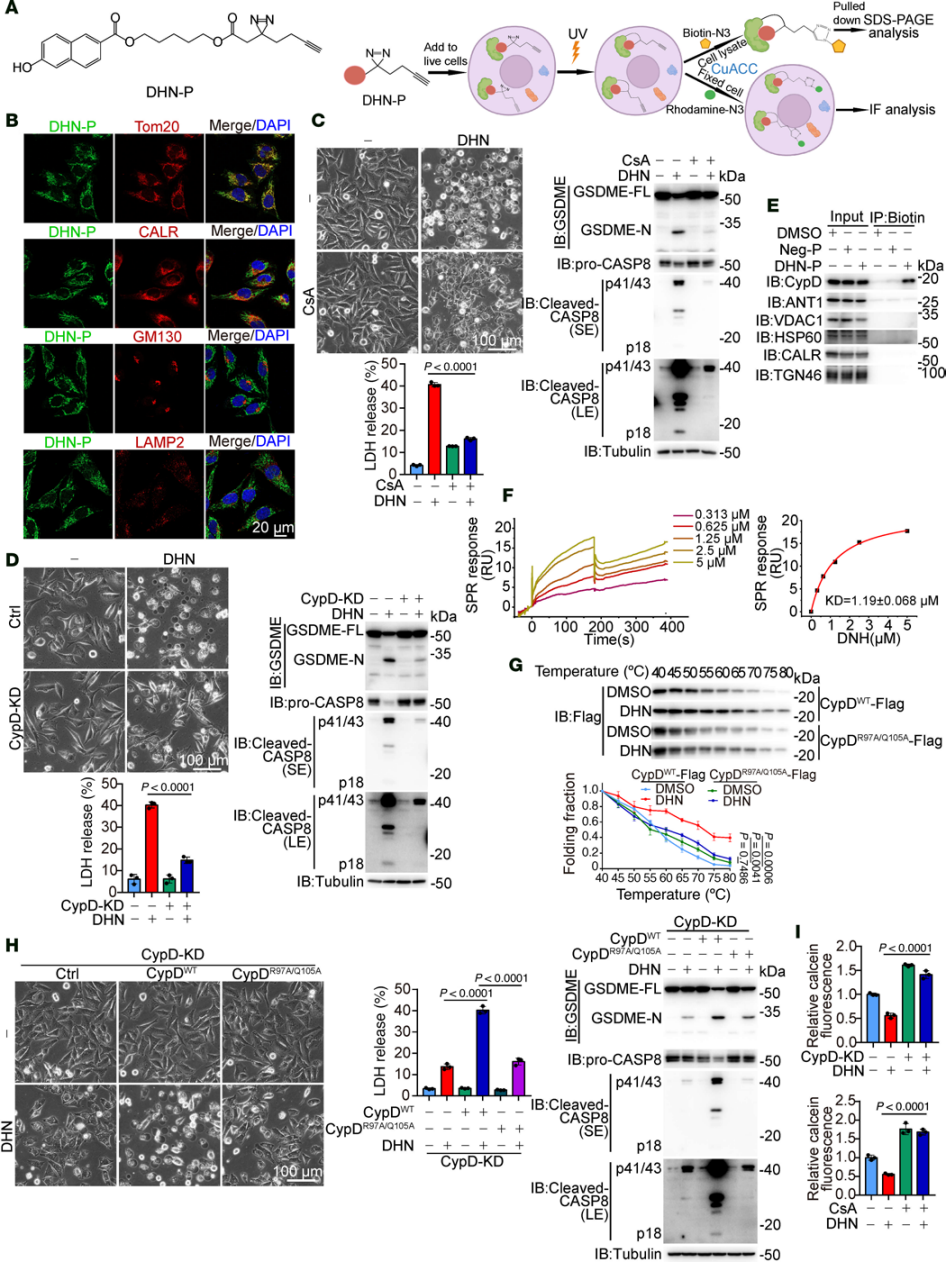
DHN promotes the opening of mPTP by targeting mitochondrial protein CypD. Melanoma A375 cells were treated with DHN (15 μM) for 20 hours to assess pyroptotic features (including characteristic morphology, caspase8/GSDME cleavage, and LDH release), unless specifically defined. (**A**) Chemical structure of DHN probe (DHN-P, left) and workflow of click chemistry for DHN-P (right). (**B**) Cells were treated with DHN-P (150 μM) for 2 hours. Azide-rhodamine was conjugated with DHN-P, and the localization of DHN-P is shown (Tom20, mitochondria marker; CALR, ER marker; GM130, Golgi marker; LAMP2, lysosomal marker). Scale bar: 20 μm. (**C** and **D**) Cells were treated with DHN in the presence of CsA (**C**, 5 μM) or in CypD-knockdown cells (**D**), followed by the detection of pyroptosis. Scale bars: 100 μm. (**E**) Cells were treated with DHN-P (150 μM) for 2 hours; azide-biotin was added to conjugate with DHN-P. DHN-P–targeted CypD was assayed by streptavidin beads. (**F**) The binding affinity between DHN and CypD was determined by surface plasmon resonance. (**G**) Cellular thermal shift assay. The proteins of CypD^WT^ or CypD^R97A/Q105A^ were immunoprecipitated from cells, followed by treatment with DHN and subsequent differential temperature incubation for 15 minutes. Resulting lysates were subjected to Western blot analysis. (**H**) CypD^WT^ or CypD^R97A/Q105A^ were separately transfected into CypD-knockdown cells, followed by detection of pyroptosis. Scale bar: 100 μm. (**I**) CypD was knocked down in cells, or cells were cotreated with CsA (5 μM) for 12 hours, followed by detection of mPTP opening. Tubulin was used to determine the amount of loading protein. DAPI was used to indicate nucleus in confocal microscopy. Data are presented as mean ± SEM of 3 independent experiments. Statistical analyses were determined by 2-way ANOVA with Tukey’s multiple-comparison test (**C**, **D**, **G**, **H**, and **I**). *P* values are indicated in figures. All Western blots were repeated at least twice, and 1 of them is shown.

We aimed to elucidate the target of DHN by synthesizing a photoactive DHN probe (referred to as DHN-P) that exhibited similar properties to DHN in inducing pyroptosis (Supplemental Figure 2A). We detected the subcellular localization of DHN-P using click chemistry (Figure 2A) and found that DHN-P predominantly colocalized with Tom20, a mitochondrial marker protein, whereas minimal colocalization was observed with CALR (an ER marker), GM130 (a Golgi apparatus marker), or LAMP2 (a lysosomal marker) (Figure 2B), indicating that mitochondria may be the organelle for DHN function. Our previous studies have demonstrated that the induction of pyroptosis is associated with an upregulation of various ROS (2, 7, 8, 22). We thus investigated whether mitochondrial ROS (mito-ROS) are involved in DHN-induced pyroptosis. Treatment with DHN indeed resulted in a significant increase in mito-ROS levels; however, the scavenging of mito-ROS by mito-TEMPO or mitoQ failed to rescue DHN-induced pyroptosis (Supplemental Figure 2, B and C), which excluded the association between mito-ROS and DHN-induced pyroptosis. To elucidate the crucial mitochondrial functions underlying DHN-induced pyroptosis, we employed various inhibitors, and the results indicated that inhibition of the electron transport chain by antimycin A, rotenone, or oligomycin; suppression of the TCA cycle by CPI-613 or dimethyl malonate; and attenuation of fatty acid oxidation by ranolazine had no impact on DHN-induced pyroptosis. Furthermore, modulation of mitochondrial fission through Mdivi-1 treatment or regulation of mitochondrial calcium homeostasis via MCU-i4 did not affect DHN-induced pyroptosis either (Supplemental Figure 2C). However, cyclosporin A (CsA), an inhibitor targeting cyclophilin D (CypD) within the mPTP complex, markedly blocked DHN-induced caspase-8 activation and GSDME-mediated pyroptotic cell death (Figure 2C). Similar results were also obtained in CypD knockdown cells (Figure 2D). Given that DHN-P could effectively pull down CypD but not other components in the mPTP complex, such as adenine nucleotide translocator1 (ANT1) and voltage-dependent anion channel 1 (VDAC1), or proteins in the ER (CALR) or Golgi apparatus (TGN46) (Figure 2E), it is likely that DHN targets CypD for pyroptotic induction.

To further verify that CypD is the direct target of DHN, we conducted surface plasmon resonance experiments and confirmed the direct interaction between DHN and CypD, with a *K_D_* of 1.19 ± 0.068 μM (Figure 2F). We additionally performed fluorescence labeling–based differential scanning fluorimetry (FL-DSF), a well-established method for evaluating protein-ligand interactions (23). The FL-DSF assay yielded a *K_D_* value of 0.69 ± 0.33 μM (Supplemental Figure 2D). We also performed cellular thermal shift assays to detect drug-target interactions through analyzing melting temperature shifts. Addition of DHN significantly enhanced the thermal stability of CypD (Figure 2G), indicating direct binding of DHN to CypD. Molecular docking indicated the theoretical binding mode of DHN to CypD (PDB: 5CBV), in which the naphthalene ring of DHN formed a distinct cationic π-interaction with R97 of CypD, and Q105 formed a hydrogen bond with the oxygen atom while the hydrophobic carbon chain of DHN lay flat in the pocket (Supplemental Figure 2E). When these 2 critical residues were mutated (CypD^R97A/Q105A^), DHN could no longer bind to CypD (Figure 2G). As a result, DHN failed to induce GSDME cleavage and pyroptosis in CypD^R97A/Q105A^-expressing cells (Figure 2H). Therefore, DHN induces pyroptosis through binding to CypD.

Considering the crucial role of CypD in mPTP (24), we propose that DHN may regulate mPTP opening by targeting CypD. Indeed, DHN augmented mPTP opening, which was effectively inhibited by either CypD knockdown or CsA treatment (Figure 2I). The essential role of mPTP opening in DHN-induced pyroptosis is further supported by our observation that knockdown of ANT1, another constituent of the mPTP, also impaired DHN-induced mPTP opening, GSDME cleavage, and subsequent pyroptosis (Supplemental Figure 2, F and G). Together, these findings suggest that DHN binding to CypD facilitates mPTP opening, ultimately leading to pyroptosis.

### DHN-induced mtDNA release activates the cytosolic cGAS.

**Figure 3:**
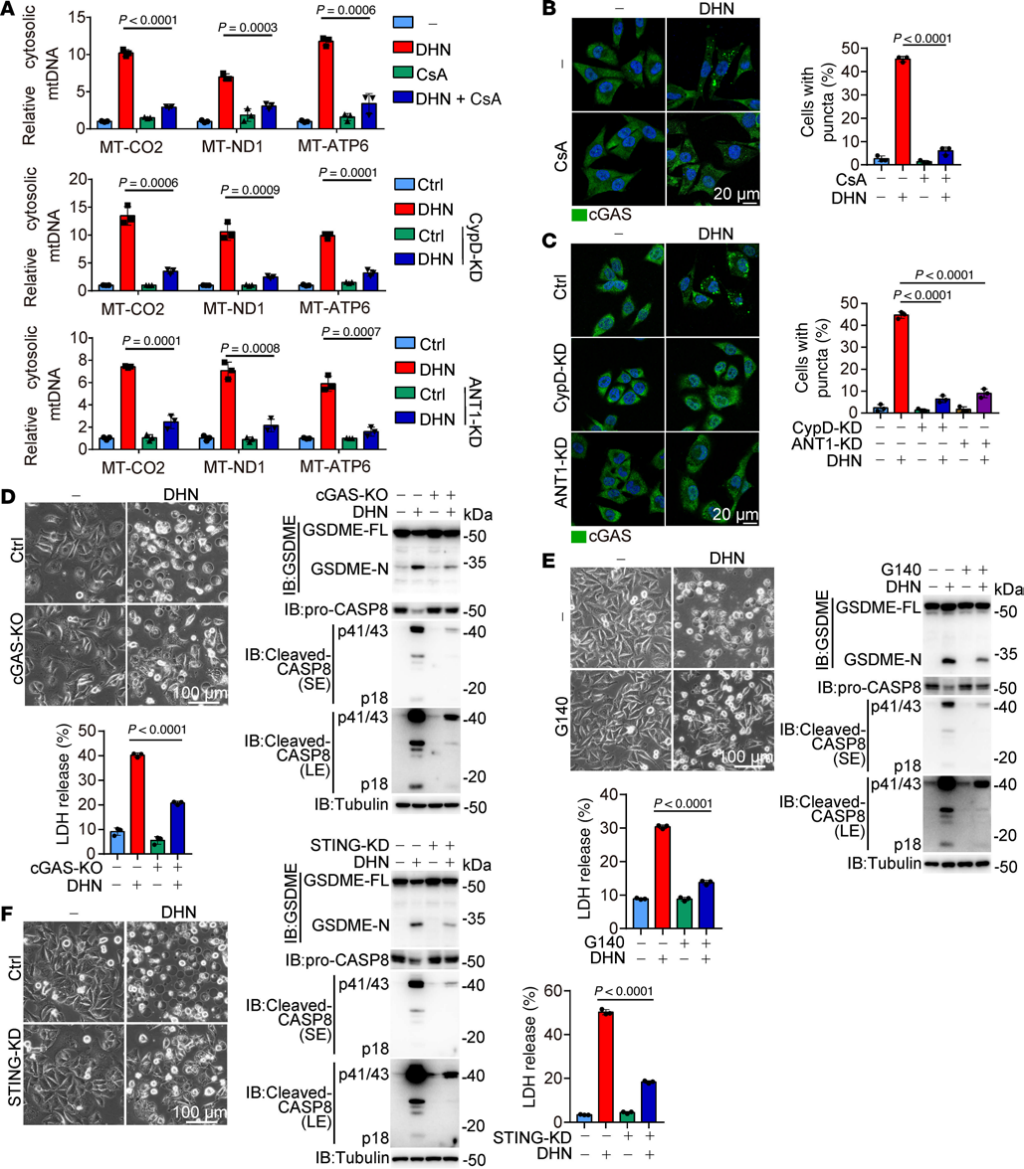
DHN-induced mtDNA release activates the cytosolic cGAS. Melanoma A375 cells were treated with DHN (15 μM) for 12 hours to show the puncta of cGAS and detect the release of mtDNA; and for 20 hours to assess pyroptotic features (including characteristic morphology, caspase8/GSDME cleavage, and LDH release), unless specifically defined. (**A**) Cells were cotreated with CsA (top, 5 μM), and CypD (middle) or ANT1 (bottom) was knocked down in cells, followed by detection of mtDNA release. (**B** and **C**) Cells were cotreated with CsA (**B**, 5 μM), and CypD or ANT1 (**C**) was knocked down in cells, then stained with anti-cGAS antibody. cGAS puncta were observed under confocal microscope. Scale bars: 20 μm. The percentage of cells with cGAS puncta was quantified (right, mean ± SEM, *n* = 3 repeats). The quantification and counting of 100 cells were performed 3 times in a single experiment, and the average value obtained from the 3 statistical measurements was recorded as 1 repetition. (**D**–**F**) Cells were cotreated with G140 (**E**, 30 μM), cGAS was knocked out (**D**), or STING was knocked down (**F**) in cells, followed by the detection of pyroptosis. Scale bars: 100 μm. Tubulin was used to determine the amount of loading protein. Data are presented as mean ± SEM of 3 independent experiments. Statistical analyses were determined by 2-way ANOVA with Tukey’s multiple-comparison test (**A**–**F**). *P* values are indicated. All Western blots were repeated at least twice, and 1 of them is shown.

It has been reported that several NLRP3 inflammasome activators can induce the oxidation of mtDNA, resulting in the release of 500–650 bp fragments into the cytosol through mPTP- and VDAC-dependent channels (25). Upon DHN stimulation, we also observed a significant increase in the amount of mtDNA in the cytoplasmic fraction that was free from mitochondrial contamination (Figure 3A and Supplemental Figure 3A). This release of mtDNA was effectively suppressed by treatment with CsA or knockdown of ANT1 or CypD (Figure 3A and Supplemental Figure 3A), emphasizing the crucial role played by mPTP in DHN-induced mtDNA release. However, no obvious oxidation of mtDNA was detected upon DHN stimulation (Supplemental Figure 3B), and DNA fragments in the cytosolic samples within the range of 500–700 bp were barely observed via agarose gel electrophoresis (Supplemental Figure 3C). Moreover, while an approximately 600 bp mtDNA fragment in the cytosolic fraction was clearly detected by PCR after DHN treatment, PCR also successfully amplified an approximately 5,000 bp mtDNA fragment (Supplemental Figure 3D), suggesting that DHN may induce the release of mtDNA fragments exceeding 5,000 bp in length. Given that only mtDNA fragments smaller than 700 bp can be released upon mPTP opening (26), it is unlikely that DHN induces direct mtDNA release from mitochondria via the mPTP channel. It has been documented that prolonged mPTP opening leads to mitochondrial rupture (27, 28). Indeed, DHN-induced mitochondrial rupture was clearly observed by transmission electron microscopy. Furthermore, the release of HSP60, a mitochondrial matrix protein, into the cytosol upon DHN stimulation confirmed mitochondrial rupture (Supplemental Figure 3E). The DHN-induced release of HSP60 and mtDNA could be effectively suppressed by CsA treatment or CypD knockdown (Supplemental Figure 3, F and G). Therefore, it can be concluded that DHN may promote prolonged mPTP opening, leading to mitochondrial rupture and subsequent mtDNA release.

Consistent with a previous report that cytoplasmic DNA triggers phase transition of cGAS to activate its activity (29), we did observe the formation of cGAS puncta in response to DHN stimulation (Figure 3B), and this DHN-induced cGAS puncta formation could be abolished by treatment with 1,6-hexanediol (1,6-HD) (Supplemental Figure 3H), a small molecule known for melting phase–separated condensates, indicating the phase transition of cGAS upon DHN stimulation. Furthermore, CsA treatment or knockdown of CypD or ANT1 abrogated the formation of these DHN-induced cGAS puncta (Figure 3, B and C). The ability of DHN to induce cGAS puncta formation was lost when the interaction between CypD and DHN was disrupted by the R97A/Q105A mutation in CypD (Supplemental Figure 3I). These findings demonstrate a direct link between mPTP-mediated mtDNA release and activation of cGAS upon binding of DHN to CypD.

The activation of cGAS is crucial for DHN-induced pyroptosis, as evidenced by the effective attenuation of DHN-induced GSDME cleavage and pyroptosis through silencing cGAS expression or inhibiting cGAS activity using inhibitor G140 (30) (Figure 3, D and E). Combined with the finding that neither knockdown of cGAS nor G140 treatment affected the DHN-induced mtDNA release (Supplemental Figure 3J), these experiments indicate that it is the release of mtDNA that activates cGAS and induces pyroptosis. It is well accepted that STING serves as a downstream effector of cGAS (10). Indeed, knockdown of STING markedly impaired DHN-induced GSDME cleavage and pyroptosis (Figure 3F). However, DHN treatment failed to induce the phosphorylation of TBK1 and IRF3 (Supplemental Figure 3K), the downstream kinases in the classical cGAS/STING pathway (10), or to regulate the transcription levels of classical downstream target genes associated with cGAS/STING signaling (including *CXCL10*, *IFNB*, *RSAD2*, *ISG15*, and *RIG1*) (Supplemental Figure 3L). Moreover, inhibition of TBK1 by GSK8612 (31) did not affect DHN-induced pyroptosis (Supplemental Figure 3M). These results appear to show that DHN may activate an alternative pathway within the cGAS/STING axis to induce pyroptosis.

### The aggregation of STING in the ER provides the platform for GSDME cleavage.

**Figure 4:**
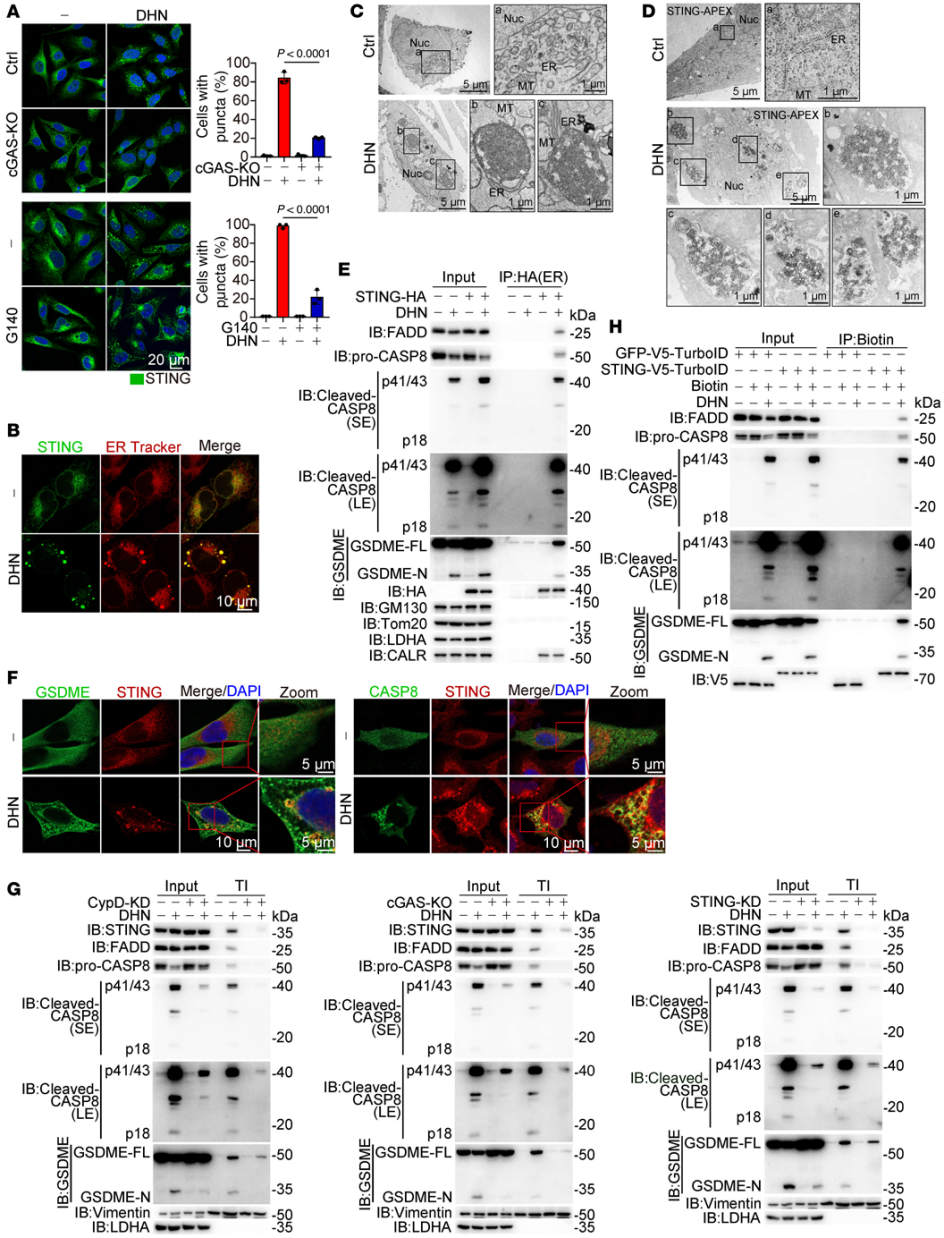
DHN induces the formation of STING aggregates to recruit caspase-8 and GSDME. Melanoma A375 cells were treated with DHN (15 μM) for 12 hours to show the puncta of STING in the ER and detect various proteins in the Triton X-100–insoluble (TI) fractions, unless specifically defined. (**A**) cGAS was knocked out in cells, or cells were cotreated with G140 (30 μM), and stained with STING antibody. STING puncta were observed under confocal microscope (left). Scale bars: 20 μm. The percentage of cells with STING puncta was quantified (right, mean ± SEM, *n* = 3 repeats). (**B**) Living cells were treated with DHN; puncta of STING and ER shown. Scale bars: 10 μm. (**C** and **D**) Observation of STING-associated ER structure using electron microscopy. A375 cells (**C**) or STING/APEX-expressing A375 cells (**D**) were treated with DHN for 12 hours; the ER morphology and the location of STING in ER was observed. Scale bars: 1 μm and 5 μm. (**E**) Indication of STING-associated organelles. Cells were transfected with STING-HA, the STING-associated organelles were immunoprecipitated, and then indicated by various antibodies (CALR, ER marker; GM130, Golgi marker; Tom20, mitochondria marker; LDHA, cytosol marker). (**F**) Cells were treated with DHN; puncta of STING and GSDME or caspase-8 shown. Scale bars: 5 μm and 10 μm. (**G**) The CypD-knockdown (left), STING-knockdown (right), or cGAS-knockout (middle) cells were treated with DHN. The localization of STING, cleaved CASP8, and GSDME in the TI is indicated. (**H**) Cells were transfected with GFP-V5-turboID or STING-V5-turboID and then labeled with biotin (100 μM) for 10 minutes; the biotin-labeled proteins were isolated and indicated by corresponding antibodies. Data are presented as mean ± SEM of 3 independent experiments. Statistical analyses were determined by 2-way ANOVA with Tukey’s multiple-comparison test (**A**). *P* values are indicated. All Western blots were repeated at least twice, and 1 of them is shown.

We also discovered that DHN could induce the formation of punctate structures of STING in a manner dependent on cGAS activity (Figure 4A) and mPTP opening (Supplemental Figure 4, A and B). However, these STING puncta were not colocalized with cGAS (Supplemental Figure 4C), suggesting that the STING puncta are distinct from cGAS puncta. Knockdown of STING did not affect DHN-induced formation of cGAS puncta (Supplemental Figure 4D). Notably, these STING puncta were exclusively localized to the ER rather than the Golgi apparatus or mitochondria (Figure 4B and Supplemental Figure 4E), resulting in obvious puncta appearance within the ER in a cGAS- and STING-dependent manner (Supplemental Figure 4F). Transmission electron microscopy revealed condensed membranous structures resembling aggregates within the ER upon DHN stimulation (Figure 4C), which could be abolished by cGAS inhibitor G140 (Supplemental Figure 4G) or knockdown of STING (Supplemental Figure 4H). Additionally, in cells expressing STING-APEX fusion protein, the APEX signal was prominently observed within these tangled ER structures upon DHN stimulation (Figure 4D). Therefore, it is likely that DHN induces the aggregation of STING in the ER through activating cGAS.

It has been reported that phase separation of STING within the ER can prevent excessive activation of classical cGAS/STING signaling (32). However, treatment with 1,6-HD failed to disrupt DHN-induced punctate aggregates of STING (Supplemental Figure 4I), and expression of STING^EE/GG^ mutants, known to abolish the STING phase separator (32), had no effect on these aggregates either (Supplemental Figure 4J). These results suggest that the DHN-induced STING aggregate in the ER (termed ER-STING aggregate) is distinct from the STING phase separator reported in another study (32) and may play a role in pyroptosis induction.

To investigate the function of these ER-STING aggregates in pyroptotic induction, we employed a detergent-free immunoprecipitation technique using an anti-HA antibody to selectively isolate ER-STING aggregates in HA-STING–expressing A375 cells. The immunoprecipitants were found to contain the ER protein CALR, while Golgi protein GM130 and mitochondrial protein Tom20 were not detected (Figure 4E), confirming the absence of contamination from the Golgi apparatus or mitochondria. Western blotting showed the activated caspase-8, full-length GSDME, and GSDME N-terminal within these ER-STING aggregates upon DHN stimulation (Figure 4E). Confocal microscopy consistently indicated the colocalization of caspase-8 and GSDME with STING puncta in the presence of DHN (Figure 4F). These results suggest the recruitment of caspase-8 and GSDME into the ER-STING aggregates. Since protein aggregates are typically resistant to mild detergents like Triton X-100, we fractionated these ER-STING aggregates into a Triton X-100–insoluble (TI) fraction. DHN stimulation obviously increased STING levels in the TI fraction, in which active caspase-8, full-length GSDME, and cleaved-GSDME were also detected (Figure 4G). The full-length GSDME appeared to be more enriched in the TI fraction compared with the cleaved-GSDME, which is consistent with previous findings that upon cleavage, cleaved-GSDME tends to localize to the plasma membrane for pyroptosis execution (6). Knockdown of CypD, cGAS, or STING or inhibition of cGAS activity using G140 resulted in loss of active caspase-8 and GSDME within the STING aggregates even in the presence of DHN (Figure 4G and Supplemental Figure 4K). Proximity labeling assays also demonstrated that DHN enhanced proximity between STING and caspase-8/GSDME (Figure 4H). Considering the role of STING as a scaffold protein (33), it is proposed that DHN-induced ER-STING aggregate represents a large protein complex, potentially serving as a platform to recruit and process the cleavage of GSDME by caspase-8 for pyroptosis induction.

We further explored the underlying mechanism of caspase-8 activation within the ER-STING aggregates. Fas-associated death domain (FADD), an adaptor protein essential for death receptor–mediated caspase-8 activation (34), was investigated for its role in this process. Our results demonstrated that DHN stimulation substantially enhanced the interaction between STING and FADD (Supplemental Figure 4L). This STING-FADD interaction is essential for the recruitment of caspase-8 by STING in response to DHN stimulation, as knockdown of FADD completely abolished the interaction between STING and caspase-8, even in the presence of DHN (Supplemental Figure 4L). Consequently, no caspase-8 was detected in STING-dependent protein aggregates upon FADD knockdown. Furthermore, FADD knockdown also eliminated DHN-induced caspase-8 activation, GSDME cleavage, and pyroptosis (Supplemental Figure 4M). The death effector domain plays a pivotal role in the mutual interaction between FADD and caspase-8. In FADD-knockdown cells, reexpression of WT FADD but not FADD^ΔDED^ restored DHN-induced caspase-8 activation, GSDME cleavage, and pyroptosis (Supplemental Figure 4N). Similarly, in caspase-8 knockdown cells, reexpression of WT caspase-8, instead of caspase-8^ΔDED^, restored DHN-induced GSDME cleavage and pyroptosis (Supplemental Figure 4O). Collectively, these findings indicate that DHN-induced interaction of STING with FADD promotes the recruitment of caspase-8 into ER-STING aggregates, where caspase-8 is activated to cleave GSDME for pyroptosis execution.

### An acid environment promotes the polymerization of STING for the formation of ER-STING aggregates.

**Figure 5:**
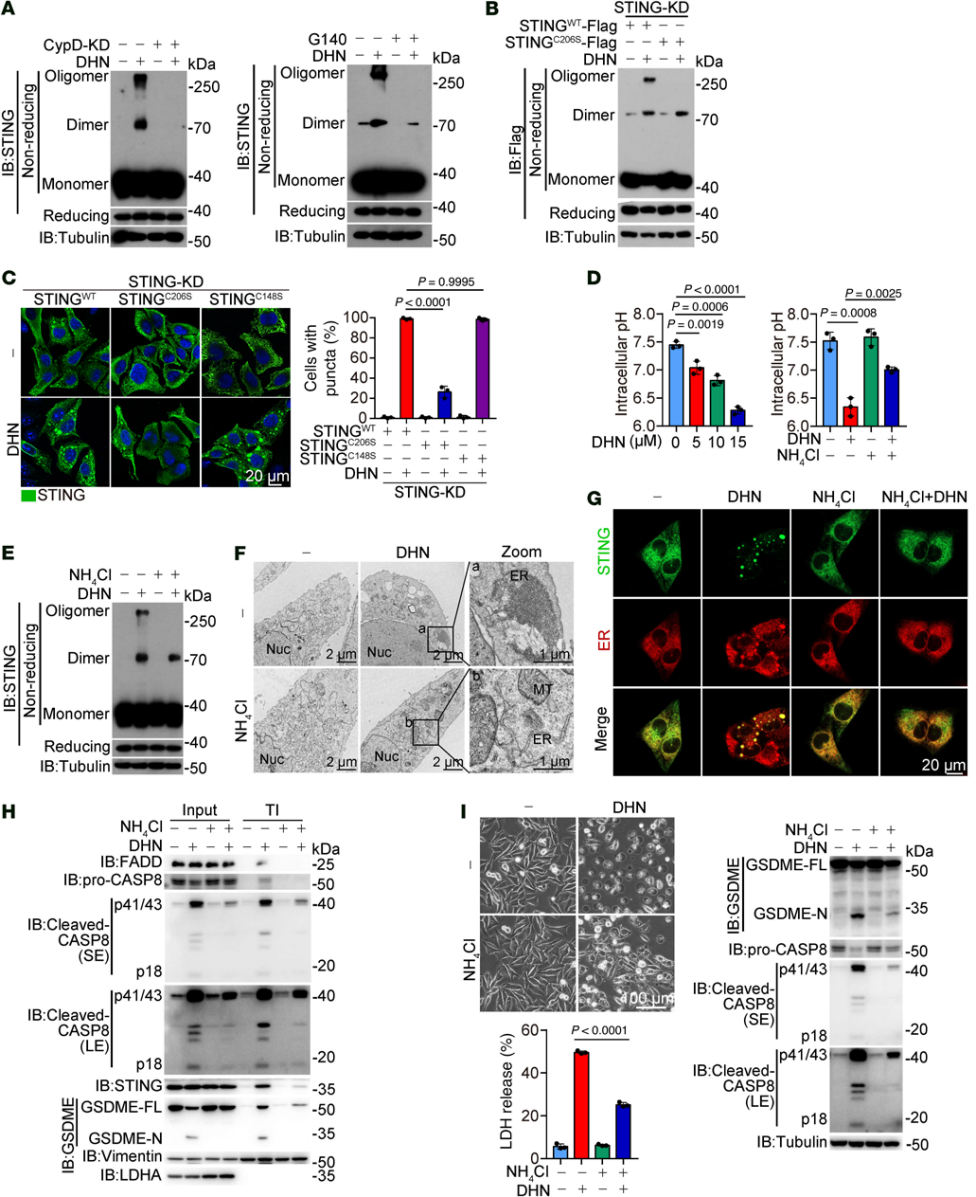
DHN-caused ER acid environment promotes the formation of STING aggregates. Melanoma A375 cells were treated with DHN (15 μM) for 12 hours to show the puncta of STING in the ER and to detect STING polymer and location of various proteins in TI and for 20 hours to assess pyroptotic features, unless specifically defined. (**A**) CypD was knocked down in cells (**A**, left), or cells were cotreated with G140 (**A**, right, 30 μM), and then polymer of STING was indicated. (**B** and **C**) STING^WT^, STING^C206S^, and STING^C148S^ were separately transfected into STING-knockdown cells, then polymer of STING was indicated (**B**). STING puncta were observed under confocal microscope (**C**, left); scale bar: 20 μm. The percentage of cells with STING puncta was quantified (**C**, right). (**D**) Cells were treated with DHN at different concentrations (left), or were cotreated with NH_4_Cl (right, 5 mM), followed by measurement of cytosolic pH values. (**E**–**I**) Cells were cotreated with NH_4_Cl (5 mM), followed by detection of polymer of STING (**E**); ER morphology using electron microscope (**F**), scale bars: 1 μm and 2 μm (zoom); STING and ER puncta using confocal microscope (**G**), scale bar: 20 μm; the localization of STING, cleaved-CASP8, and GSDME in the TI (**H**); and pyroptosis, scale bar: 100 μm (**I**). Tubulin was used to determine the amount of loading protein. Data are presented as mean ± SEM of 3 independent experiments. Statistical analyses were determined by 1-way ANOVA with Tukey’s multiple-comparison test (**D**, left) and 2-way ANOVA with Tukey’s multiple-comparison test (**C** and **D**, right; and **I**). *P* values are indicated. All Western blots were repeated at least twice, and 1 of them is shown.

Since the formation of protein aggregates typically results from protein polymerization (35), we investigated whether DHN-induced ER-STING aggregate is associated with the polymerization of STING. As anticipated, DHN markedly enhanced the formation of STING dimers and polymers, which could be abolished by knockdown of CypD and ANT1 (Figure 5A and Supplemental Figure 5A) or inhibition of cGAS by G140 (Figure 5A), indicating that the opening of mPTP and cGAS activity are essential for DHN-induced STING polymerization. Cys148 and Cys206 in STING play critical roles in the occurrence of STING polymers (36, 37). Mutation at Cys206 but not at Cys148 abrogated DHN-induced STING polymerization (Figure 5B and Supplemental Figure 5B). Although C206S mutation did not influence STING dimerization, it impaired the formation of ER-STING aggregates (Figure 5C) and subsequent pyroptotic cell death (Supplemental Figure 5C). It is likely that DHN-induced opening of mPTP and subsequent activation of cGAS facilitate the polymerization of STING, and that this STING polymerization, rather than dimerization, leads to the formation of ER-STING aggregates.

cGAMP is the product of activated cGAS (38). Interestingly, incubation of cGAMP with A375 cells induced the dimerization of STING in accordance with previous reports (36); however, it barely induced the polymerization of STING, the formation of ER-STING aggregates, and pyroptosis (Supplemental Figure 5, D–F), suggesting that cGAS activation alone is insufficient for STING polymerization and that other unknown factors may be involved in DHN-induced pyroptosis. Considering our finding that caspase-8 exhibited unique enhanced activity in an acidic environment to cleave GSDME (Supplemental Figure 1I) and that DHN, a naphthol derivative, has the property of being a weak acid, we hypothesized that DHN might acidify the intracellular environment that benefits pyroptotic induction. Indeed, treatment with DHN, but not cGAMP, effectively induced dose-dependent acidification of the intracellular milieu (Figure 5D and Supplemental Figure 5G). NH_4_Cl significantly rescued DHN-induced decline of intracellular pH (Figure 5D), which was closely associated with a series of events, including inhibited STING polymerization (Figure 5E), the suppression of ER-STING aggregates (Figure 5, F and G), the decrease of caspase-8 activation and GSDME cleavage in TI fraction (Figure 5H), and the inhibition of DHN-induced pyroptosis (Figure 5I). Clearly, an acidic intracellular environment induced by DHN is a prerequisite for the formation of ER-STING aggregates and pyroptosis.

Considering the ER localization of the ER-STING aggregates, we speculated that the pH level of the ER might be altered. To this end, we first developed a reliable method for assessing ER pH, wherein the double ratio variation in different pH values served as an indicator of the detection system (Supplemental Figure 5H). Utilizing this approach, we demonstrated that DHN did acidify the ER environment in a dose-dependent manner and this acidification could be rescued by NH_4_Cl treatment as expected (Supplemental Figure 5I), in accordance with the results observed in the cytoplasm. These findings align with previous knowledge that the ER lacks an intrinsic pH regulatory system and readily equilibrates its pH to cytoplasmic levels (39).

**Figure 6:**
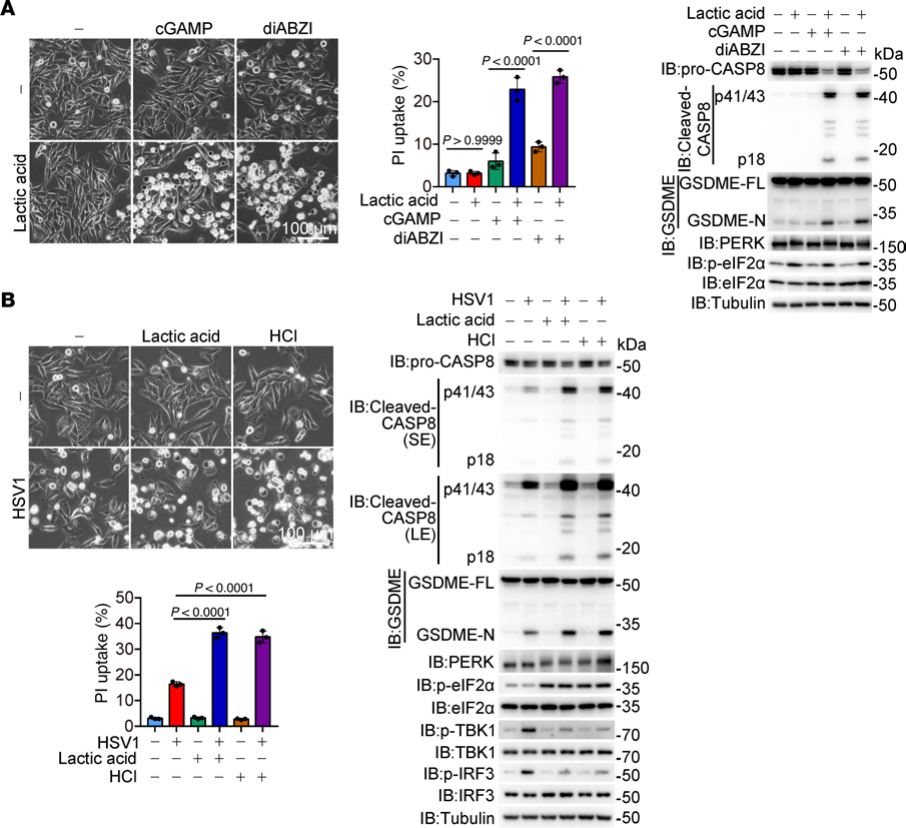
cGAS activation converges with intracellular acidification to induce pyroptosis. Melanoma A375 cells were treated with different stimulants for 20 hours to assess pyroptotic features (including characteristic morphology, caspase8/GSDME cleavage, and LDH release), unless specifically defined. (**A**) Cells were cotreated with lactic acid (20 mM) and 2,3′-GAMP (10 μg/mL) or diABZI (10 μM), followed by the detection of pyroptosis. (**B**) Cells were infected with HSV1 (10 MOI) in the presence of lactic acid (20 mM) or HCl (20 mM), followed by detection of pyroptosis. Tubulin was used to determine the amount of loading protein. Data are presented as mean ± SEM of 3 independent experiments. Statistical analyses were determined by 2-way ANOVA with Tukey’s multiple-comparison test. Scale bars: 100 μm (**A** and **B**). *P* values are indicated. All Western blots were repeated at least twice, and 1 of them is shown.

Since NH_4_Cl failed to regulate DHN-induced cGAS puncta formation (Supplemental Figure 5J), and the decline in intracellular or ER pH caused by DHN remained unaffected by inhibition of CypD or STING (Supplemental Figure 5, K and L), we postulated that cGAS activation and ER acidification are 2 independent pathways that synergistically contribute to the formation of ER-STING aggregates and subsequent pyroptosis. To further validate this hypothesis, we simulated cGAS activation with cGAMP or diABZI treatment while inducing a decrease in intracellular and ER pH through lactic acid treatment (Supplemental Figure 5M), which is abundant in the tumor microenvironment and known to lower intracellular pH levels (40, 41). We demonstrated that either cGAMP/diABZI treatment alone or lowering pH by lactic acid alone was insufficient to induce pyroptosis; however, simultaneous treatment with cGAMP/diABZI and lactic acid clearly induced GSDME cleavage and subsequent pyroptosis (Figure 6A). Similar phenomena were also observed upon cotreatment of HCl, but not sodium lactate, with cGAMP (Supplemental Figure 5, N and O). Knockdown of LDHA/LDHB (which are essential for lactate metabolism) or AARS1/AARS2 (involved in protein lactylation) (34, 42) had no effect on pyroptosis induced by cGAMP plus lactic acid (Supplemental Figure 5P), thereby excluding the involvement of lactate metabolism or protein lactylation. Moreover, infection with HSV1, a DNA virus known to activate cGAS (43), could also trigger GSDME-mediated pyroptosis in the presence of lactic acid or HCl (Figure 6B). In conclusion, when both cGAS activation and intracellular acidification are simultaneously achieved, STING-dependent GSDME-mediated pyroptosis occurs.

### PERK-induced STING phosphorylation facilitates the polymerization of STING.

**Figure 7:**
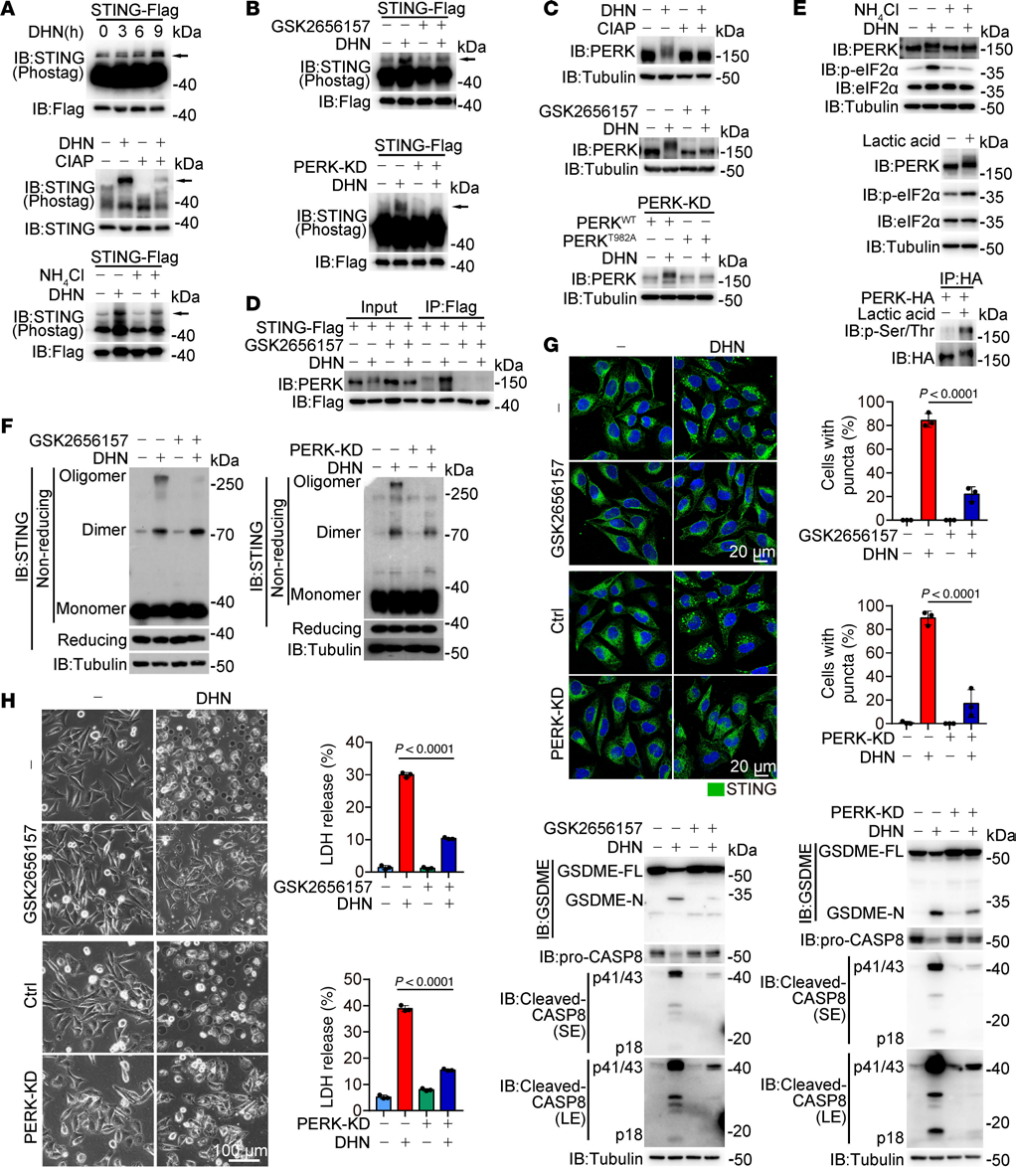
DHN-induced phosphorylation of STING by PERK facilitates the polymerization of STING. Melanoma A375 cells were treated with DHN (15 μM) for 12 hours to detect STING phosphorylation, the puncta of STING in the ER, and STING polymer and location of various proteins in TI; and for 20 hours to assess pyroptotic features, unless specifically defined. (**A** and **B**) Control (**A**, top) or PERK-knockdown A375 cells (**B**, bottom) were treated with DHN in the presence of NH_4_Cl (**A**, bottom, 5 mM) or GSK2656157 (**B**, top, 10 μM). Cell lysates were incubated with calf intestinal alkaline phosphatase (CIAP) (**A**, middle). STING phosphorylation was analyzed using Phos-tag assays. (**C**) Cell lysates were incubated with CIAP (top). Cells were cotreated with GSK2656157 (middle, 10 μM), and PERK^WT^ or PERK^T982A^ were separately transfected into PERK-knockdown cells (bottom). (**D**) Cells were cotreated with GSK2656157 (10 μM); the interaction between STING and PERK was determined. (**E**) Cells were cotreated with NH_4_Cl (top, 5 mM) and DHN, or treated with lactic acid (middle and bottom, 20 mM), followed by the detection of PERK and eIF2α phosphorylation. (**F**–**H**) Cells were cotreated with GSK2656157 (10 μM) or subjected to PERK knockdown, followed by detection of STING polymerization (**F**); STING puncta (**G**), scale bar: 20 μm; and pyroptosis, scale bar: 100 μm (**H**). Tubulin was used to determine the amount of loading protein. Data are presented as mean ± SEM of 3 independent experiments. Statistical analyses were determined by 2-way ANOVA with Tukey’s multiple-comparison test (**G** and **H**). *P* values are indicated. All Western blots were repeated at least twice, and 1 of them is shown.

The mechanism by which an acidic environment facilitates STING polymerization and the formation of ER-STING aggregates remains unclear. We unexpectedly found that DHN induced a time-dependent phosphorylation of STING, as evidenced by the appearance of an upshifted band in a Phos-tag gel, which was abolished when cell lysates were incubated with calf intestinal alkaline phosphatase (CIAP) (Figure 7A). Notably, treatment with NH_4_Cl substantially impaired DHN-induced STING phosphorylation (Figure 7A), demonstrating the association between intracellular acidity and STING phosphorylation. Considering the subcellular localization of STING in the ER, we employed various inhibitors, including GSK2656157 targeting PERK, GSK650394 targeting SGK1, GSK2850163 targeting IRE1α, LRRK2-IN-1 targeting LRRK2, sorafenib targeting FLT3, lenvatinib targeting KDR, and HG-9-91-01 targeting SIK to identify the specific ER-resident protein kinase involved in STING phosphorylation. Among these inhibitors, only GSK2656157 effectively impaired DHN-induced STING phosphorylation (Figure 7B and Supplemental Figure 6A), indicating that PERK was a crucial mediator of STING phosphorylation upon DHN stimulation. Knockdown of PERK inhibited DHN-induced STING phosphorylation (Figure 7B and Supplemental Figure 6B). It has been reported that PERK can be activated through autophosphorylation at the Thr982 residue (44). DHN treatment effectively enhanced PERK phosphorylation (Figure 7C), and mutation of Thr982 or inhibition of PERK activity by GSK2656157 eliminated DHN-induced PERK phosphorylation and the association of PERK with STING (Figure 7, C and D). Therefore, these findings suggest that DHN promotes autophosphorylation and activation of PERK, leading to the association of PERK with STING and subsequent STING phosphorylation.

Recent studies have suggested that cGAS/STING activation directly triggers PERK activation (18). In contrast, inhibition of mtDNA release (CsA treatment or CypD knockdown) or suppression of cGAS/STING activity (G140 treatment or STING knockdown) did not affect PERK phosphorylation upon DHN stimulation (Supplemental Figure 6D), excluding the involvement of cGAS/STING in PERK activation during DHN treatment. Instead, NH_4_Cl treatment profoundly suppressed DHN-induced PERK activation (Figure 7E), and a decrease in intracellular pH caused by lactic acid, HCl, or citric acid was sufficient to induce PERK phosphorylation and activation (Figure 7E and Supplemental Figure 6E). Combined with the fact that inhibition of PERK by GSK2656157 or knockdown of PERK did not affect DHN-induced acidification (Supplemental Figure 6F), it could be concluded that PERK is activated when there is a decline in intracellular pH induced by DHN treatment.

**Figure 8:**
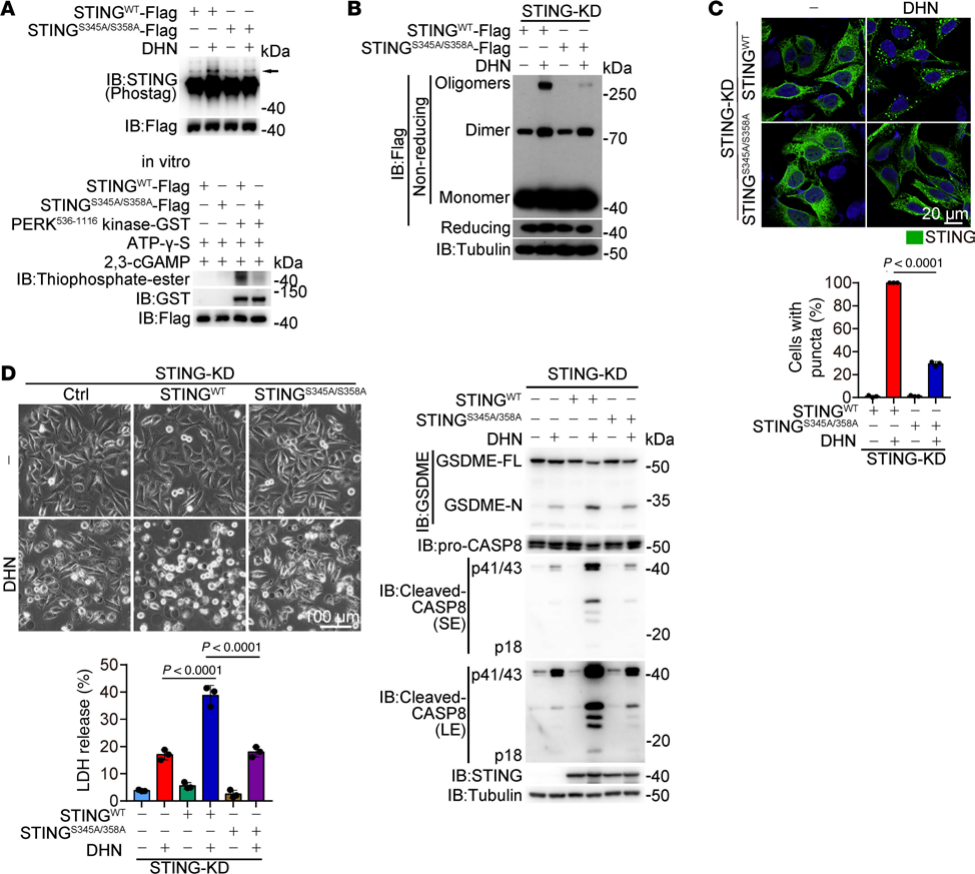
Phosphorylation of STING at Ser345 and Ser358 by PERK is critical for DHN-induced pyroptosis. Melanoma A375 cells were treated with DHN (15 μM) for 12 hours to detect STING phosphorylation and the puncta of STING in the ER; and for 20 hours to assess pyroptotic features, unless specifically defined. (**A**) STING^WT^ and STING^S345A/S358A^ were transfected into STING-knockdown cells, followed by the detection of STING phosphorylation (top). STING^WT^ and STING^S345A/S358A^ was incubated with PERK in vitro (bottom). (**B**–**D**) STING^WT^ and STING^S345A/S358A^ were transfected into STING-knockdown cells, followed by detection of STING polymerization (**B**); STING puncta (**C**), scale bar: 20 μm; and pyroptosis, scale bar: 100 μm (**D**). Tubulin was used to determine the amount of loading protein. Data are presented as mean ± SEM of 3 independent experiments. Statistical analyses were determined by 2-way ANOVA with Tukey’s multiple-comparison test (**C** and **D**). *P* values are indicated. All Western blots were repeated at least twice, and 1 of them is shown.

Although inhibition of PERK did not affect cGAS puncta formation (Supplemental Figure 6G), it markedly impaired DHN-induced STING polymerization (but not dimerization), formation of ER-STING aggregates, cleavage of GSDME by caspase-8 within ER-STING aggregates, and subsequent pyroptosis (Figure 7, F–H, and Supplemental Figure 6, H–K), demonstrating the crucial role of PERK in STING polymerization but not in cGAS activation. According to the PhosphoSitePlus database, there are 4 potential phosphorylated Ser/Thr residues in STING, including Thr84, Ser345, Ser358, and Ser366 (45, 46). Substituting alanine for either Ser345 or Ser358 weakly impaired DHN-induced STING phosphorylation (Supplemental Figure 6L), while combined mutation of these 2 residues (STING^S345A/358A^) resulted in the abrogation of DHN-induced STING phosphorylation in A375 cells and PERK-induced STING phosphorylation in vitro (Figure 8A), leading to elimination of the polymerization of STING, formation of ER-STING aggregates, GSDME cleavage by caspase-8 in the TI fraction, and subsequent pyroptosis (Figure 8, B–D, and Supplemental Figure 6M). Considering that the Cys206 mutation, which abolishes STING polymerization (Figure 5B), had no impact on DHN-induced STING phosphorylation (Supplemental Figure 6N), it is plausible that PERK-mediated phosphorylation of STING serves as an upstream event, which facilitates the assembly of STING polymers upon DHN stimulation, thereby contributing to pyroptosis.

Although inhibition of cGAS did not affect DHN-induced PERK activation (Supplemental Figure 6D), it markedly impaired DHN-induced STING phosphorylation (Supplemental Figure 6O). Considering the essential role of cGAMP in STING function (47), we postulated that the interaction between cGAMP and STING may contribute to PERK-mediated phosphorylation of STING. Addition of cGAMP dramatically enhanced the phosphorylation of STING by PERK in an in vitro kinase assay (Supplemental Figure 6P). STING^R238A/Y240A^, which lacks binding affinity for cGAMP (47), failed to undergo DHN-induced phosphorylation (Supplemental Figure 6Q). Consequently, cells expressing STING^R238A/Y240A^ were resistant to DHN-induced pyroptosis (Supplemental Figure 6R). Therefore, it appears that the interaction between STING and cGAMP functions as a structural priming event for subsequent PERK-mediated STING phosphorylation. In this context, both DHN-induced release of mtDNA (which activates cGAS to generate cGAMP) and intracellular acidity (required for PERK activation) are indispensable for the formation of ER-STING aggregates and induction of pyroptosis.

### Pathological inhibitory effects of DHN on tumor growth in mice.

**Figure 9:**
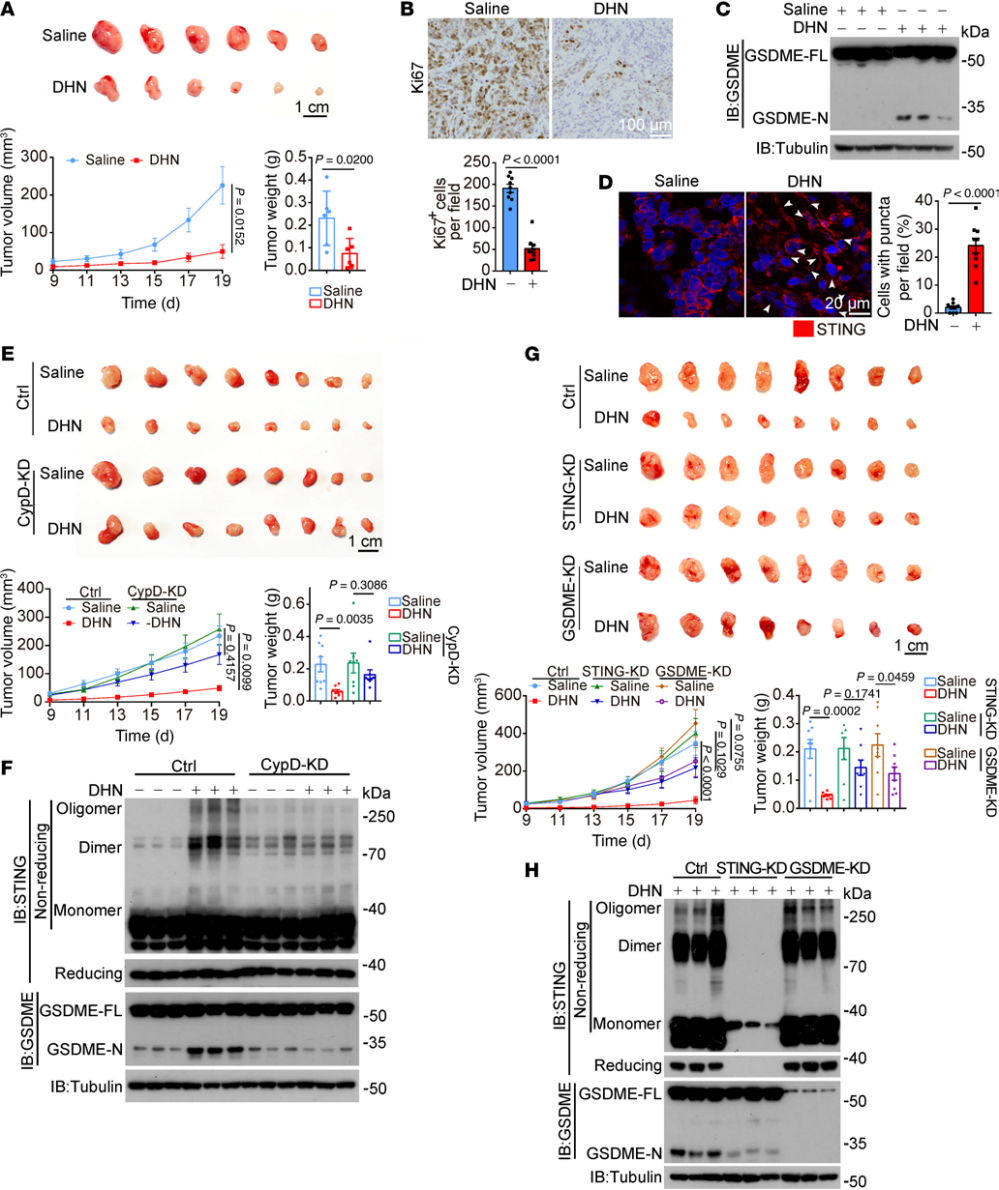
DHN inhibits tumor growth by inducing pyroptosis in mice. A375 cells (2 × 10^6^) were injected subcutaneously into the posterior flanks of nude mice. After 4 days, DHN was intraperitoneally administered to the mice every other day for 2 weeks. The tumor volume and weight were recorded at the indicated times. (**A**–**D**) A375 cells were injected into BALB/c-nu mice to form subcutaneous xenografts (**A**, *n* = 6, scale bar: 1 cm). The expression of Ki67 is shown (**B**; *n* = 9 fields from 3 independent tumor tissues; scale bar: 100 μm). Tumors were collected for detection of GSDME (**C**). STING puncta are indicated by white arrows (**D**, left, scale bar: 20 μm), and the percentage of cells with STING puncta was quantified (**D**, right; *n* = 9 fields from 3 independent tumor tissues). (**E**–**H**) A375 cells with or without knockdown of CypD (**E** and **F**). Scale bar: 1 cm (**E**). STING or GSDME (**G** and **H**) was injected into BALB/c-nu mice to form subcutaneous xenografts (*n* = 8). Tumors were collected for detection of GSDME and monomers, dimers, and oligomers of STING. Scale bar: 1 cm (**G**). Tubulin was used to determine the amount of loading protein. Statistical analyses were determined by unpaired 2-tailed Student’s *t* test (**A**, **B**, and **D**) and 2-way ANOVA with Tukey’s multiple-comparison test (**E** and **G**). *P* values are indicated.

**Figure 10:**
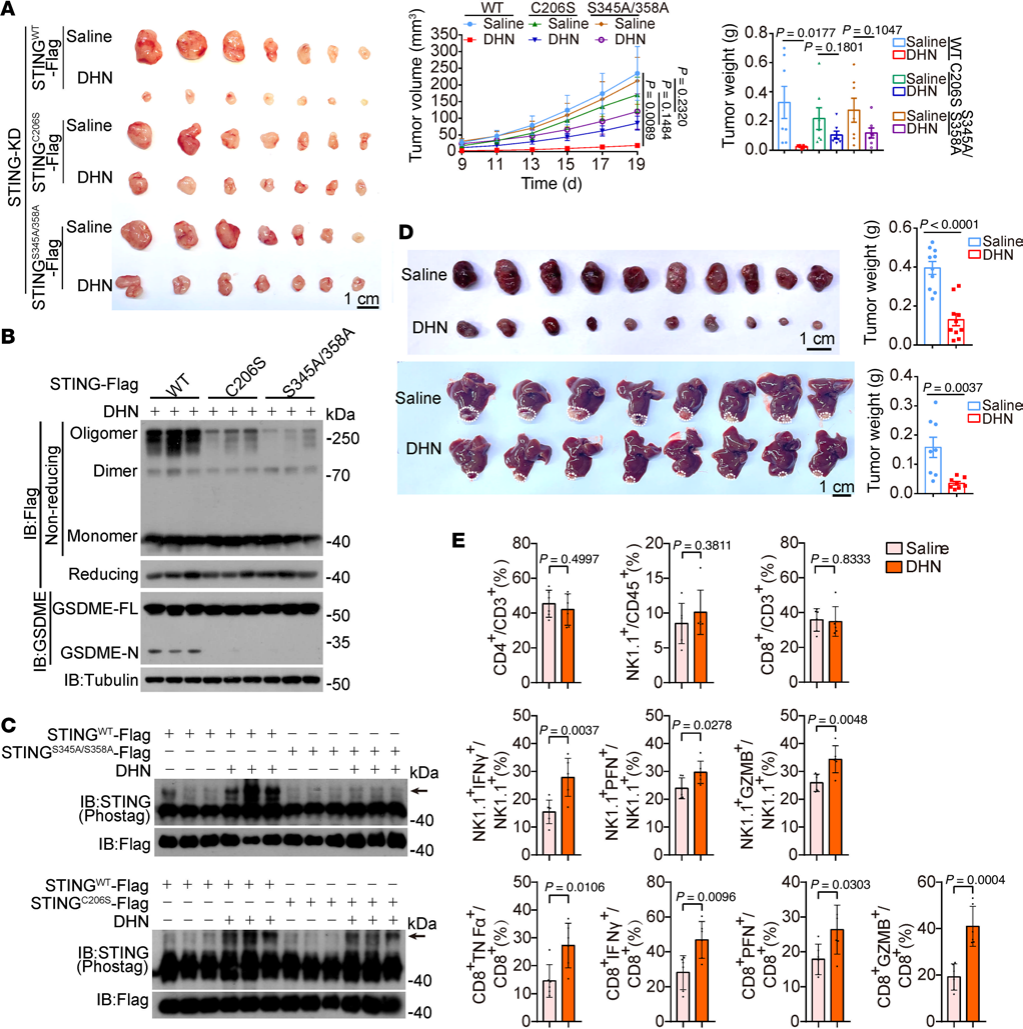
DHN induces antitumor immune responses in a mouse tumor model. A375 cells (2 × 10^6^) were injected subcutaneously into the posterior flanks of nude mice. After 4 days, DHN was intraperitoneally administered to the mice every other day for 2 weeks. The tumor volume and weight were recorded at the indicated times. (**A**–**C**) A375 STING-knockdown cells with expression of STING^WT^, STING^C206S^, or STING^S345A/358A^ were injected into BALB/c-nu mice to form subcutaneous xenografts (**I**, *n* = 7). Tumors were collected for detection of GSDME (**A**), scale bar: 1 cm; monomers, dimers, and oligomers of STING (**B**); and STING phosphorylation (**C**). (**D**) B16 (top, *n* = 10) or Hepa1-6 (bottom, *n* = 8) cells were injected into C57BL/6 mice to form xenografts. DHN was intraperitoneally administered to the mice. Scale bar: 1 cm. (**E**) B16 cell–derived xenograft tumors were collected 24 hours after DHN (10 mg/kg) administration and then analyzed using flow cytometry to determine the proportion and activation status of immune cells within the tumor microenvironment (*n* = 5). Tubulin was used to determine the amount of loading protein. Statistical analyses were determined by unpaired 2-tailed Student’s *t* test (**D** and **E**) and 2-way ANOVA with Tukey’s multiple-comparison test (**A**). *P* values are indicated.

To evaluate the in vivo antitumor efficacy of DHN, nude mice with A375 cell–derived xenografts were utilized. Intraperitoneal administration of DHN significantly suppressed the growth of xenograft tumors, as evidenced by reduced tumor weight and size compared with the control group (Figure 9A). We also observed that DHN treatment significantly decreased the expression of Ki67, a marker of cell proliferation in tumors (Figure 9B). The inhibitory effect of DHN was closely associated with GSDME cleavage (Figure 9C) and the formation of punctate structures of STING (Figure 9D) in tumor tissues. Furthermore, knockdown of CypD, the target protein of DHN, greatly impaired the suppressive effect of DHN on tumor growth as well as STING polymerization and GSDME cleavage (Figure 9, E and F), emphasizing the requirement of CypD for the DHN effect. To further elucidate the involvement of STING and GSDME, nude mice bearing xenografts derived from A375 cells with knocked down STING or GSDME were also administered DHN. As expected, knockdown of either STING or GSDME almost abolished the inhibitory effect exerted by DHN (Figure 9, G and H). Moreover, expression of STING^C206S^ (abolishing STING polymerization) or STING^S345A/358A^ (abolishing PERK-mediated STING phosphorylation) significantly attenuated the effect of DHN on tumor growth (Figure 10A), accompanied by the decrease in STING polymerization and phosphorylation detected in the same tumor samples (Figure 10, B and C). Collectively, these results demonstrate that the inhibitory effect of DHN in vivo is indeed a result of pyroptosis induction and is closely associated with the CypD/STING/GSDME axis.

To further evaluate the antitumoral efficacy of DHN in immunocompetent mouse models, we assessed its ability to induce pyroptosis in B16 (mouse melanoma cells) and Hepa1-6 (mouse hepatocellular carcinoma cells). Our findings demonstrated that DHN effectively induced STING oligomerization, GSDME cleavage, and pyroptosis in both cell lines (Supplemental Figure 7A). Subsequently, we employed B16 and Hepa1-6 cells to establish orthotopic xenografts in C57BL/6 immunocompetent mice. Our findings revealed that the administration of DHN markedly inhibited tumor growth derived from both B16 and Hepa1-6 cells (Figure 10D). Notably, although DHN treatment had minimal impact on the proportions of CD4^+^ T cells, CD8^+^ T cells, or NK cells within the tumor microenvironment, it markedly enhanced the activation status of these cells, as evidenced by increased proportions of IFN-γ^+^, TNF-α^+^, perforin (PFN)^+^, and granzyme B^+^/CD8^+^ T cells as well as IFN-γ^+^, PFN^+^, and granzyme B^+^/NK cells (Figure 10E). Therefore, it could be concluded that this noncanonical cGAS/STING pathway–associated pyroptosis exhibits immunogenic properties and holds potential for inducing antitumor immune responses.

Last, we assessed the pharmacokinetic/pharmacodynamic properties of DHN. To this end, we conducted a dosing study in the B16-derived xenograft model in C57BL/6 mice. The results demonstrated that a concentration of 20 mg/kg exhibited a significant effect in suppressing xenograft growth. However, a concentration of 40 mg/kg did not yield more pronounced effects (Supplemental Figure 7B). Dose-related blood exposure of DHN in these mice was also observed, with a time at maximal concentration (*t*_max_) of approximately 2 hours, correlating with dose-dependent exposure in tumor tissues. The *t*_1/2_ of DHN was approximately 4.5 hours. Furthermore, DHN-induced GSDME cleavage in tumor tissues peaked at 24 hours after injection (Supplemental Figure 7C), which aligns with the observation that DHN induced pyroptosis after 24 hours in melanoma cells, indicating its prolonged therapeutic effect. Notably, DHN administration at doses up to 40 mg/kg had a negligible effect on body weight. The weights and histological structures of the heart, liver, spleen, and kidney remained unchanged after DHN treatment. Plasma levels of the creatine kinase myocardial band, an indicator of cardiac injury; aspartate aminotransferase and alanine aminotransferase (ALT and AST), indicators of hepatic damage; and blood urea nitrogen (BUN), an indicator of renal injury, revealed minimal evidence of organ damage. Additionally, DHN did not affect colon length (Supplemental Figure 7D). Therefore, it can be concluded that DHN exhibits minimal toxicity in mice.

## Discussion

The cGAS/STING signaling pathway has emerged as a pivotal mediator of innate immunity and is closely associated with antitumoral immune responses. STING agonists, either alone or in combination with immune checkpoint blockade therapy, are currently being clinically investigated for their potential efficacy in anticancer treatment. However, the direct induction of tumor cell death through manipulation of this pathway remains unknown. In this study, we identified a compound, DHN, that specifically induces pyroptosis in tumor cells through the noncanonical cGAS/STING pathway. On the one hand, DHN targets CypD in the mitochondria to induce the opening of the mPTP complex, resulting in the release of mtDNA into the cytoplasm, where it activates cGAS to produce cGAMP. On the other hand, DHN causes intracellular acidification to activate PERK. The association of cGAMP with STING serves as a priming step for subsequent PERK-induced STING phosphorylation. This phosphorylation of STING not only retains it in the ER but also facilitates its polymerization to form aggregates, thereby establishing a platform (i.e., ER-STING aggregates) to recruit caspase-8 and GSDME for further cleavage, ultimately triggering pyroptosis. Overall, our findings identify DHN as a pyroptosis inducer with promising implications for antitumor therapy and elucidate an alternative signaling pathway that connects the noncanonical cGAS/STING pathway and an acid environment to pyroptotic induction.

CypD, a well-established sensitizer of the mPTP, is a key component targeted by many pharmacological inhibitors including CsA, the gold-standard inhibitor of the mPTP (24). However, to the best of our knowledge, the existence of an activator specifically targeting CypD for mPTP opening remains unknown. In this study, we demonstrated DHN is an activator of mPTP through direct binding to CypD. Structurally, CsA specifically binds to the active site of CypD without interacting with the residues located in the S2 pocket, a substrate-binding groove adjacent to the active site (48, 49). In contrast, molecular modeling and point mutation analysis suggest that the naphthalene ring of DHN interacts with residues near the active site, while its long hydrophobic carbon chain of DHN lies flat in the S2 pocket, exhibiting a distinct binding mode compared with CsA’s interaction with CypD. This distinctive binding mode might generate an altered protein surface capable of modulating CypD’s associations with binding partners. Considering that the interaction between CypD and components of the mPTP complex, such as ATP synthase or ANT, regulates mPTP opening (24), it is plausible that DHN regulates the association between CypD and the mPTP complex to facilitate its opening, which warrants further investigation in the future. However, neither CsA treatment nor the knockdown of CypD or ANT1 completely abolished mtDNA release upon DHN stimulation. This suggests that other factors may also partially participate in regulating DHN-induced mtDNA release.

The opening of mPTP is closely associated with the release of mtDNA into the cytosol, subsequently triggering innate immune responses by activating the cGAS/STING pathway (25, 50). Although DHN-induced mPTP opening indeed stimulated mtDNA release and activated cGAS, it failed to activate the innate immune response, as evidenced by the absence of TBK1 and IRF3 phosphorylation, as well as transcriptional induction of type I IFNs and IFN-stimulated genes, suggesting a noncanonical activation of the cGAS/STING pathway upon DHN stimulation. This may be attributed to PERK-mediated STING phosphorylation, which hinders STING translocation from the ER to the Golgi apparatus — a prerequisite for TBK1 and IRF3 activation (51). Previous studies have reported that TGF-β–activated kinase 1–mediated phosphorylation at Ser355 is crucial for STING ER exit (52). Considering that in the current case, PERK phosphorylates STING at residues Ser345 and Ser358 in close proximity to TAK1 phosphorylation sites, it is not surprising that PERK-mediated phosphorylation could suppress STING ER exit. In this context, PERK-mediated phosphorylation may act as a functional switch determining whether STING remains in or exits from the ER.

The PERK-mediated retention of STING in the ER leads to noncanonical activation of STING, characterized by the formation of condensed membranous structures within the ER containing multiprotein complexes. This unique subcellular architecture recruits and processes GSDME cleavage by caspase-8 to induce pyroptosis, demonstrating a distinct compartmental regulation for pyroptosis induction. The formation of disulfide bonds at Cys206 is crucial for STING polymerization and subsequent assembly of ER-STING aggregates. In contrast, in the canonical cGAS/STING pathway, STING oligomerization is facilitated by disulfide bonds formed between Cys148 residues on each STING molecule (36). This discrepancy suggests that upon phosphorylation by PERK and retention in the ER, different residues are utilized by STING for polymerization, resulting in a distinct surface geometry necessary for recruiting specific binding partners, such as caspase-8 and GSDME, instead of TBK1 or IRF3. Consequently, this leads to the formation of ER-STING aggregates and induction of pyroptosis rather than activation of innate immunity. In this regard, Cys206-dependent STING polymerization favors noncanonical STING activation, which may partially explain the close association between individuals with mutations at Cys206 of STING and SAVI (STING-associated vasculopathy with onset in infancy), a disease characterized by systemic inflammation resulting from constitutive activation of the canonical STING pathway (53, 54).

DHN-induced PERK activation is not associated with mtDNA release and cGAS activation, but rather linked to DHN-mediated intracellular acidification. Although the exact mechanism underlying this acidification is still not well understood, it is plausible that the weak acidic nature of DHN may at least partially contribute to it. Maintaining a constant intracellular pH is crucial for normal cellular functions since almost all cellular processes rely on a stable pH level (55, 56). Therefore, fluctuations in intracellular pH can modulate intracellular signaling transduction, and protons are considered second messengers in this context (57). In addition to DHN stimulation, we found that a decrease in intracellular pH was sufficient to activate PERK, suggesting that PERK could be responsive to the decline of intracellular pH and that there is a connection between fluctuation of pH and the cGAS/STING pathway. However, considering that changes in intracellular acidity also affect protein folding (56), it is also plausible that PERK, being a key component of the unfolded protein response, gets activated indirectly under conditions of decreased pH.

DHN-induced acidification of the ER also plays a crucial role in the cleavage of GSDME by caspase-8 in ER-STING aggregates. It has been suggested that caspase-8 promotes GSDME processing through activating caspase-3 (5). However, our findings demonstrate that caspase-8 can directly cleave GSDME at Asp270 under acidic conditions, consistent with the optimal pH requirement for caspase-8 being less than 7 (58). In this regard, the compartmental pH fluctuations within cells may serve as an overlooked regulatory pathway for caspase activation and cell death induction, warranting further investigation in future studies.

In summary, we found that DHN induces GSDME cleavage by caspase-8 and subsequent pyroptosis by activating a noncanonical cGAS/STING pathway, which relies on both cGAS activation and STING phosphorylation by PERK. Importantly, this noncanonical cGAS/STING pathway–associated pyroptosis is not limited to DHN stimulation; simultaneous activation of cGAS and reduction in intracellular acidity are sufficient for pyroptosis induction. Pathologically, given that glycolysis-generated lactic acid is one of the major contributors to intracellular acidity in tumors (59), the induction of cytoplasmic DNA in glycolysis-dependent tumor cells under conditions such as defects in the DNA damage response, chromosomal instability, replicative stress, reactivation of endogenous retroelements, or the release of mtDNA (60) may lead to atypical STING activation to some extent. This phenomenon warrants further investigation.

## Methods

Additional details on methods can be found in the Supplemental Methods.

### Sex as a biological variable.

In this study, sex was not considered as a biological variable. All mice used in our study were male for easier handling and management purposes. It is unknown whether the findings are relevant for female mice.

### Cell culture and transfection.

The human melanoma cell line A375, murine melanoma cell line B16, and human hepatoma cell line Huh7 were obtained from Cell Bank in the Chinese Academy of Sciences. HEK293T cells; melanoma cell lines IgR3, M14, and MEL-RM; human monocyte-like cell line THP-1; cervical cancer cell line HeLa; mouse hepatoma cell line Hepa1-6; human hepatoma cell line Plc; human kidney proximal tubule epithelial cell line HK-2; human cardiomyocyte cell line AC16; mouse cardiomyocyte cell line HL-1; mouse myoblast cell line C2C12; and mouse fibroblast cell line L929 were obtained from American Type Culture Collection. The human melanoma cell line MV3 was obtained from Xiamen Immocell Biotechnology Co. The human ccRCC cell line 786-O was obtained from Procell Life Science & Technology. The primary mouse BM-derived macrophages and BM-derived DCs were isolated from C57BL/6 mice. These cell lines were cultured in medium supplemented with 10% FBS. During the drug treatment period, the FBS concentration was reduced from 10% to 2%. Cell transfection was performed using TurboFect transfection reagent (Thermo Fisher Scientific), except for HEK293T cells that were transfected using the calcium phosphate method.

### Microscopy.

Cells were seeded in a 6-well plate at a confluence of 40%–60% and treated with various pharmacological agents to evaluate morphological alterations associated with pyroptosis. Subsequently, phase-contrast images were captured using a Nikon TE2000 microscope. For confocal microscopy, cells were rinsed with DMEM, fixed in 4% paraformaldehyde, blocked with blocking buffer (3% BSA and 0.2% Triton X-100), and subsequently incubated overnight at 4°C with the appropriate primary antibodies: anti-Tom20 (Cell Signaling Technology, catalog 42406S), anti-CALR (Cell Signaling Technology, catalog 12238), anti-GM130 (Proteintech, catalog 11308-1-AP), anti-LAMP2 (Abcam, catalog ab25631), anti-STING (Proteintech, catalog 19851-1-AP), anti-cGAS (ABclonal, catalog 15102), anti-Flag (Sigma-Aldrich, catalog F-1804), anti-PERK (ABclonal, catalog 18196). Next, cells were washed with washing buffer (0.2% BSA and 0.05% Triton X-100) and incubated with secondary antibodies: goat anti-rabbit Alexa Fluor 594 (Thermo Fisher Scientific, catalog A-11037), goat anti-mouse Alexa Fluor 594 (Thermo Fisher Scientific, catalog A-11005), goat anti-rabbit Alexa Fluor 488 (Thermo Fisher Scientific, catalog A-11008), goat anti-mouse Alexa Fluor 488 (Thermo Fisher Scientific, catalog A-11001) for 1 hour at 37°C. Nuclei were visualized by staining with DAPI at a concentration of 50 μg/mL for 5 minutes. Confocal images were acquired using a Zeiss LSM 980 confocal microscope. For the phase separation of depolymerization assay, cells were treated with 5% 1,6-HD for 10 minutes to induce phase separation, followed by processing as described for confocal microscopy.

### LDH release assay.

The activity of LDH released into the cell culture supernatants was measured using the CytoTox 96 Non-Radioactive Cytotoxicity assay kit (Promega) to assess pyroptosis, according to the manufacturer’s instructions.

### Immunoblotting.

Cells were lysed using ELB lysis buffer (containing 150 mM NaCl, 100 mM NaF, 50 mM Tris-HCl at pH 7.6, and 0.5% Nonidet P-40), supplemented with a cocktail of protease inhibitor and phosphatase inhibitor. After centrifugation of the cell lysates at 14,000*g* at 4°C for 15 minutes, the supernatants were combined with an equal volume of 2× SDS loading buffer and boiled for 15 minutes. Subsequently, the samples underwent SDS-PAGE followed by transfer onto a PVDF membrane for immunoblotting analysis using specific antibodies. The details of the primary antibodies are provided in the Supplementary Materials section under “Antibodies”. For STING polymer analysis, samples were prepared with or without β-mercaptoethanol in both reduced and nonreduced forms. Cell lysates were collected in 2× SDS loading buffer excluding β-mercaptoethanol. Subsequently, the samples were separated using either SDS-PAGE or 4%–12% gradient gel electrophoresis.

### Mouse models.

Male BALB/c nude mice (7–8 weeks old, weighing 18–22 g) and C57BL/6 mice (7–8 weeks old) were procured from the SLAC Laboratory Animal Center in China and housed at the Laboratory Animal Center of Xiamen University. They were housed in a controlled environment with a 12-hour light/12-hour dark cycle and provided ad libitum access to food and water.

The A375 cell xenograft tumor model was established by subcutaneously injecting 1 × 10^6^ A375 cells in 100 μL DMEM into the anterior flanks of BALB/c nude mice. After 4 days, the mice were divided into different groups: treatment with vehicle (10% DMSO in saline, 100 μL per mouse) or DHN (20 mg/kg; DHN dissolved in 10% DMSO and further diluted with saline) via intraperitoneal injections every other day for 2 weeks. Subsequently, the mice were euthanized, and their body weights as well as tumor weights were recorded.

The B16 cell xenograft tumor model was established by subcutaneously injecting 1 × 10^5^ B16 cells in 100 μL DMEM into the anterior flanks of C57BL/6 mice. After 4 days, the mice were divided into different groups: treatment with vehicle (10% DMSO in saline, 100 μL per mouse) or DHN (20 mg/kg; DHN dissolved in 10% DMSO and further diluted with saline) via intraperitoneal injections once daily for 1 week. Subsequently, the mice were euthanized, and their body weights as well as tumor weights were recorded.

The Hepa1-6 cell orthotopic xenograft tumor model was established by subcutaneously injecting 2 × 10^6^ Hepa1-6 cells into the subcapsular region of the left liver lobe of C57BL/6 mice. After 4 days, the mice were divided into different groups: treatment with vehicle (10% DMSO in saline, 100 μL per mouse) or DHN (20 mg/kg; DHN dissolved in 10% DMSO and further diluted with saline) via intraperitoneal injections once daily for 2 weeks. Subsequently, the mice were euthanized, and their body weights as well as tumor weights were recorded.

### Statistics.

The statistical analyses were conducted using GraphPad Prism 9 software. The data are presented as mean ± SEM. For the comparison between 2 groups, a 2-tailed Student’s *t* test was employed. To assess differences among multiple groups, 1-way ANOVA with Tukey’s multiple-comparison test and 2-way ANOVA with Tukey’s multiple-comparison test was performed. The exact P values are indicated in figures. P < 0.05 is considered statistically significant. P < 0.01 is considered statistically highly significant.

### Study approval.

The experimental protocols involving animals were approved by the Xiamen University Animal Ethics Committee (approval XMULAC20220044).

### Data availability.

All data generated or analyzed in this study are included in this manuscript and its supplemental materials. Numerical values underlying graphical representations are provided in the Supporting Data Values file.

## Author contributions

LX, YLA, and XYM performed experiments and analyzed data. HL and FNL were responsible for the design of the compound DHN. TG and CC completed synthesis of the compound DHN. XZ and XD helped design and synthesize the probe DHN-P. LZW, QTC, and BZ provided technical advice about the experimental design and joined the discussion. WBH carried out the molecular docking analysis. LMY provided transmission electron microscope technical support and data analysis. JJC provided technical support and data analysis for surface plasmon resonance and FL-DSF. LX, YLA, QW, and HZC designed experiments, wrote the manuscript, and prepared the final scheme presentation. QW and HZC jointly supervised this work. All authors have read and approved the article.

## Supplementary Material

Supplemental data

Unedited blot and gel images

Supporting data values

## Version 1 07/15/2025

In-Press Preview

## Version 2 09/16/2025

Electronic publication
