# Supplementary material for: cGAS activation converges with intracellular acidification to promote STING aggregation and pyroptosis in tumor models
Source: J Clin Invest. 2025 Jul 15;135(18):e188872. doi: 10.1172/JCI188872 (PMC12435844; doi:10.1172/JCI188872)
Supplement: Supplemental data [file jci-135-188872-s027.pdf]

Supplementary Materials and Methods

Chemical reagents

2',3'-cGAMP (Cat# HY-100564), Rotenone (Cat# HY-B1756), CsA (Cat# HY-B0579), Liproxstatin-1 (Cat# HY-12726), Necrosulfonamide (Cat# HY-100573), Necrostatin-1 (Cat# HY-15760), TTM (Cat# HY-W076067), GSK650394 (Cat# HY-15192), GSK2850163 (Cat# HY-U00459), LRRK2-IN-1 (Cat# HY-10875), HG-9-91-01(Cat# HY-15776), Sorafenib (Cat# HY-10201) and Lenvatnib (Cat# HY-10981) were purchased from MedChemExpress. G140 (Cat# T9213), Z-IETD-FMK (Cat# T7019), MCU-i4 (Cat# T9031), GSK8612 (Cat# T5540), Ranolazine (Cat# T6633) and diABZI (Cat# T5516) were purchased from Targetmol. CIAP (Cat# 9001-78-9) was purchased from ACMEC. Biotin (Cat# A600078) was purchased from BBI. NH_4Cl (Cat# 10001508) was purchased from HUSHI. Dimethyl malonate (Cat# 966110) was purchased from J&K Scientific. 1,6-HD (Cat# A601513) was purchased from Sangon. GSK2656157 (Cat# S7033) and Mitoquinone (Cat# S8978) were purchased from Selleck. DAPI (Cat# D9542), Mdivi-1 (Cat# M0199), Ferrostatin-1 (Cat# SML0583) and Mito-TEMPO (Cat# SML0737) were purchased from Sigma-Aldrich. Oligomycin (Cat# 9996L) was purchased from Cell Signaling Technology. ER-Tracker™ Red was purchased from Thermofisher. Z-VAD-FMK (Cat# A1902) was purchased from ApexBio. Recombinant active caspase-3 (Cat# 707-C3-010/CF) was obtained from R&D Systems. Recombinant active Caspase-8 (Cat# ALX-201-062-U025) was purchased from Enzo Life Sciences.

Antibodies

Anti-GSDME (Cat# ab215191), anti-Lamp2 (Cat# ab25631), anti-GSDMD (Cat# ab210070), anti-Thiophosphate ester (Cat# ab92570), anti-IRF3 (Cat# ab68481) and Anti-Ki67 (Cat# ab16667) antibodies were purchased from Abcam. Anti-STING (Cat# 19851-1-AP), anti-CypD (Cat# 18466-1-AP), anti-Vimentin (Cat# 10366-1-AP), anti-MLKL (Cat# 66675-1-Ig) and anti-GM130 (Cat# 11308-1-AP) antibodies were purchased from Proteintech. Anti-Tom20 (Cat# 42406S), anti-caspase-9 (Cat# 9502S), anti-caspase-3 (Cat# 9665), anti-cleaved Caspase-3 (Cat# 9664), anti-Caspase-8 (Cat# 9746), anti-cleaved Caspase-8 (Cat# 9496S), anti-FADD (Cat# 2782), anti-BID (Cat# 2002), anti-cytochrome c (Cat# 11940), anti-PARP (Cat# 9532), anti-RIPK1 (Cat# 3493), anti-LDHA (Cat# 3582T), anti-VDAC1 (Cat# 4661), anti-Hsp60 (Cat# 4870), anti-CALR (Cat# 12238), anti-AIF (Cat# 4642), anti-phospho-TBK1/NAK (Cat# 5483), anti-phospho-IRF-3 (Cat# 4947) and anti-LDHA (Cat# 3582T) antibodies were purchased from Cell Signaling Technology. Anti-caspase-8 (Cat# 551244) and anti-phosphoserine/threonine (Cat# 612549) antibodies were purchased from BD Transduction Laboratories™. Anti-cGAS (Cat# 15102), anti-ANT1 (Cat# A20842), anti-TGN46 (Cat# A19618), anti-TBK1/NAK (Cat# A3458), anti V5-Tag (Cat# AE017), anti GSDMC (Cat# A14550), anti eIF2α (Cat# A21221), anti p-eIF2α (Cat# AP0692) and anti-PERK (Cat# 18196) antibodies were purchased from ABclonal. Anti-GST (Cat# SC-138) and anti-GAPDH (Cat# SC-47724) antibodies were purchased from Santa Cruz. Anti-HA (Cat# H-9658), anti-Flag (Cat# F-1804) and anti-tubulin (Cat# T-4026) antibodies were purchased from Sigma-Aldrich. The goat anti-

rabbit (Cat# 31210) and anti-mouse (Cat# 31160) secondary antibodies were purchased from ThermoFisher Scientific. BV605-anti-CD3 (Cat# 100237), PerCP-anti-CD8 (Cat# 100732), BV711-anti-IFNγ (Cat# 505836), FITC-anti-GzmB (Cat# 372206), PE-anti-perforin (Cat# 154305) and BV605-anti-TNF ((Cat# 506329) antibodies were purchased from BioLegend. APC-cy7-anti-CD45 (Cat# A15395), PE-anti-CD8 (Cat# 12-0081-82), BV711-anti-CD4 (Cat# 407-0042-80), APC-anti-NK1.1 (Cat# 17-5941-82) and APC-anti-NK1.1 (Cat# 17-5941-82) antibodies were purchased from ThermoFisher Scientific

HSV-1 Plaque Assay

HSV-1 (KOS strain) was provided by Dr. Jiahuai Han (Xianmen University) and propagated in Vero cells. Vero cells (3×10^5/well) were seeded in 12-well plates 12-24 h pre-infection. At 90-100% confluency, cells were infected with serially diluted HSV-1/2 supernatants (2 h, 37°C) with plate rocking every 30 min. Post-infection (2 h), cells were PBS-washed and overlaid with 2 ml plaquing medium (0.4% agarose/1% FBS in DMEM), prepared by mixing 4% agarose (65°C liquefied) and 1% FBS-DMEM (37°C preheated). Cells were incubated at 37°C for 3-5 d until plaque visibility. Plaques were stained with MTT (5 mg/ml in PBS, 2-4 h) and were counted.

Plasmid construction

The cDNA sequences encoding full-length CypD, caspase-8, STING, PERK and GSDME were cloned into the pLenti vector (Addgene 22255). siRNA-resistant mutants were generated by introducing silent mutations at the siRNA target sites. Mutations in

CypD (R97A/Q105A), GSDME (D229A, D251A, D256A, D267A, D270A, D279A), STING (T84A, C148A, C206S, S345A, S358A, S366A, S345A/358A), and PERK (T982A) were introduced using a QuikChange mutagenesis kit (Stratagene) and confirmed by sequencing.

Generation of the lentiviral system

Lentivirus is generated through transfection of lentiviral plasmids, packaging plasmids psPAX2 (Addgene 12260) and pMD2.G (Addgene 14887) (4 μg:3 μg:1 μg for 6-cm dish) into HEK293T cells using calcium phosphate transfection. Following a 48-hour incubation period, the culture medium containing lentivirus supernatant was collected to infect target cells with polybrene at a concentration of 10 μg/mL (Cat# H9268, Sigma). Cells exhibiting high knockdown efficiency (>80%) in terms of puromycin resistance were selected for subsequent experiments. The shRNA sequences were as follows (5′-3′):

shRNA-GSDMA: CCTCGCCTCTGTGCTCTTTAT

shRNA-GSDMB: GCTGATAGATTCCGCTGCTTC

shRNA-GSDMC: GGTGGAGTAGAGACAGTAATG

shRNA-GSDMD: GCAGGAGCTTCCACTTCTA

shRNA-GSDME: GCGGAGAATTCTTAGCATA

shRNA-Caspase8: GGAGCAACCCTATTTAGAA

shRNA-Caspase3: GGAACCAAAGATCATACATGG

shRNA-CypD: GCGTGGTGCTGGAGCTGAAGG

shRNA-ANT1: GCAGTACAAAGGGATCATTGA

shRNA-STING: TGTTGCTGCTGTCCATCTATT

shRNA-PERK: GCGAGAAGTTAAAGCCTTAGC

shRNA-FADD: TGAACTCAAGCTGCGTTTATT

shRNA1-LDHA: GGACTTGGCAGATGAACTT

shRNA2-LDHA: CTGGGAGTTCACCCATTA

shRNA-LDHB: GGAAGAAGAGGCAACAGTTCC

shRNA1-AARS1: GCAGAATAAGATGTCCAACTA

shRNA2-AARS1: CGATGTCCAGAAACGAGTGTT

shRNA1-AARS2: GCCGCCTTTCTGAACTTCTTT

shRNA2-AARS2: CCTGGTCTTCATGCAACACAA

The following oligonucleotide sequences for the lentiGuide-Puro sgRNAs were used (5′-3′):

Control: AAATGTCAGGCCGCGCCGTT

cGAS: AATATCTGTGGATATAACCC

Real-time PCR

The total RNA was extracted using TRIzol kit (Invitrogen), followed by cDNA synthesis utilizing a reverse transcriptase kit (Tiangen, Beijing, China). For specific quantitative RT-PCR experiment, the cDNA served as the amplification template with actin level employed as an internal control for normalization. The primer sequences used were as follows (5′-3′):

ISG15: forward CGCAGATCACCCAGAAGATCG

reverse, TTCGTCGCATTTGTCCACCA

RIG1: forward, CTGGACCCTACCTACATCCTG

reverse, GGCATCCAAAAAGCCACGG

RSAD2: forward, TGGGTGCTTACACCTGCTG

reverse, GAAGTGATAGTTGACGCTGGTT

CXCL10: forward, GTGGCATTCAAGGAGTACCTC

reverse, TGATGGCCTTCGATTCTGGATT

IFNβ: forward, AGTAGGCGACACTGTTCGTG

reverse, GCCTCCCATTCAATTGCCAC

GSDMA: forward, GATGTTGGGGACGTACACGAA

reverse, GGGAGCTTTGCCCTACTCG

GSDMB: forward, CTGAAAAGCGACCGGCAATA

reverse, CTTCCCTTTGGCCCTTGTGT

GSDMC: forward, CTCCAGAGTGAGGGTTTGGC

reverse, CTCTGGACTGACTGCCCATC

GSDMD: forward, CGCTGAGTGTGGACCCTAAC

reverse, ACTTCCACCTCCTTCTGTGTC

LDHB: forward, GGCTCAATCTGGTGCAGAGAA

reverse, CTCCACACAGCCACACTTGA

AARS1: forward, AGAGGGAGATCGCTGACCTT

reverse, TTCTGGACATCGGCTTTGCT

AARS2: forward, ACACTGACCTCTTTTCCCCG

reverse, GACATCCCAGGGAAGATGCC

Immunoprecipitation

Immunoprecipitation was performed according to previously established protocol (Nat Chem Biol. 2014;10(2):133-40). Briefly, cells were lysed on ice using lysis buffer (50 mM Tris, pH 7.5, 150 mM NaCl, 1 mM EDTA, 1 mM EGTA, 2.5 mM sodium pyrophosphate, 1% Triton X-100, 1 mM PMSF and protease inhibitor cocktail). The cell lysates were incubated overnight at 4 °C with the appropriate antibody followed by an additional hour of incubation with protein G-Sepharose beads at 4 °C. The beads were subjected to three washes with lysis buffer and then analyzed by western blotting.

For the ER-STING aggregates immunoprecipitation, cells were treated with 15 μM DHN for 12h. Cells were lysed in lysis buffer, and caspase-8 was immunoprecipitated with 1 μL caspase-8 antibody (CST #9746) coupled to protein A magnetic beads overnight at 4 °C. The beads were subjected to three washes with lysis buffer and then analyzed by western blotting.

The *in vitro* GSDME cleavage assay

HEK293T cells were transfected with GSDME-Flag, followed by immunoprecipitation of the GSDME protein using anti-Flag antibody together with

protein A/G agarose beads. The elution was performed with 3× Flag peptide. Subsequently, the eluents were mixed with 2× caspase reaction buffer (Caspase3 buffer: 100 mM HEPES, 6 mM EDTA, 300 mM NaCl, 20 mM DTT, 0.01% Tween-20, pH 7.5; Caspase8 buffer: 100 mM HEPES, 20 mM EDTA, 100 mM NaCl, 20 mM DTT, 0.2% CHAPS, 5% glycerol, pH7.2) at a ratio of 1:1. Following reconstitution of caspases3 or caspase8 according to the manufacturer's protocols, recombinant active caspase (final concetration: caspase3: 1.9 ug/mL and caspase8: 0.5U) was added to the eluents and incubated at 37 °C for three hours. The cleavage of GSDME was detected by western blot analysis.

SPR Assay

Surface plasmon resonance (SPR) analysis was conducted using the BIAcore8K instrument (Cytiva, USA) with a CM5 sensor chip (Cat# BR100530, Cytiva) for protein immobilization. The sensor chip was activated with a mixture of 0.2 M N-ethyl-N′-(dimethylaminopropyl)-carbodiimid-e (EDC) and 0.05 M N-hydroxysuccinimide (NHS) for 7 minutes, following the instructions in the amine-coupling kit (Cat# BR100050, Cytiva). Subsequently, a solution containing 10 μg of protein in 10 mM sodium acetate buffer (pH 4.0) was injected over the activated sensor chip for 420 seconds to immobilize the protein. The immobilization level was monitored in response units (RU) and was adjusted to approximately 5000 RU. Remaining active groups on the sensor chip were then quenched with a 1 M ethanolamine solution (pH 8.5) for 5 minutes.

Binding assays were performed at 25°C with a flow rate of 25 μL/min, utilizing Tris-T buffer (10 mM Tris, 150 mM NaCl, 0.05% Tween-20, pH 7.4, and 0.1% DMSO) as the running buffer. The assays were executed in multi-cycle mode, consisting of a 180-second association phase and a 200-second dissociation phase, with DHN concentrations ranging from 0.313 μM to 5 μM. To ensure complete removal of residual DHN between cycles, the sensor chip surface was regenerated with a gentle pulse of 10 mM glycine (pH 2.5) for 30 seconds.

The binding sensorgrams were analyzed using the BIAevaluation software of the BIAcore8K. Data were double-referenced by subtracting the responses from both the reference channel and the blank injection of the running buffer. Binding kinetics were evaluated by fitting the sensorgrams to a 1:1 Langmuir binding model using the global fitting algorithm provided by the software.

FL-DSF assay

FL-DSF assay was carried out as described previously (Int J Biol Macromol. 2024;281(Pt 1):136043). CypD proteins were labeled with FITC-NHS through the amine coupling method. A 100 mM stock solution of FITC-NHS was prepared by dissolving it in anhydrous DMSO. Proteins were diluted in 10 mM PBS buffer (pH 7.4) to a concentration of 2 mg/mL, with a dye-to-protein molar ratio optimized at 5:1. The labeling reaction was carried out at room temperature for 1 hour, followed by quenching with 10 mM Tris for 15 minutes. Excess FITC-NHS and Tris were removed using a PD-10 desalting column (Cat# 17-0851-01, Cytiva).

FL-DSF analyses were performed with a StepOne™ Plus PCR instrument (ABI, USA) in "melt curve" mode. The thermal scan was conducted from 30.0°C to 99.9°C at a heating rate of 2.33°C per minute. Binding assays were executed in 10 mM PBS buffer, with DHN concentrations ranging from 10 μM to 0.156 μM while keeping the protein concentration constant at 1 μM. Control samples containing proteins with 0.125% DMSO (v/v) without ligands were included. The binding affinity was determined using a one-site binding model based on the global fitting of melting curves (Sci Rep. 2021;11(1):2331).

FACS analyses of tumor-infiltrating immune cells

As described previously (Nat Cell Biol. 2024;26(9):1545-57), in brief, 1 × 105 B16 cells were injected subcutaneously into the flank of C57BL/6 mice. After the treatment of DHN (20 mg kg-1) daily for 7 days, tumours were collected and the tissue was digested with 5 mg ml−1 collagenase IV (Sigma, V900893) and 0.5 mg ml−1 DNase I (Sangon Biotech, B600473) to prepare a single-cell suspension. Immune cells were purified using a Percoll-gradient centrifugation method with 40% and 70% gradients. To detect tumour-infiltrating immune cells, purified immune cells were stained with cell-surface antibodies or fluorescent dyes: Zombie UV Fixable Viability kit (BioLegend, 423108), APC-cy7-anti-CD45 (Thermo Fisher Scientific, A15395), BV605-anti-CD3 (BioLegend, 100237), PE-anti-CD8 (Thermo Fisher Scientific, 12-0081-82), BV711-anti-CD4 (Thermo Fisher Scientific, 407-0042-80) and APC-anti-NK1.1 (Thermo Fisher Scientific, 17-5941-82). Besides, cells were stimulated with

PMA (1 μM, Yeasen, 50601ES03), ionomycin (2 μM, Yeasen, 50401ES03) and GolgiPlug (1,000×, BD Biosciences, 555029) for a duration of 3 h. Subsequently, the cells were stained with cell-surface antibodies: APC-cy7-anti-CD45, PerCP-anti-CD8 (BioLegend, 100732), APC-anti-NK1.1 (Thermo Fisher Scientific, 17-5941-82). Then, cells were fixed and permeabilized using Foxp3/Transcription Factor Staining Buffer Set (Thermo Fisher Scientific, 00-5523-00) and follow stained with intracellular cytokine antibodies: BV711-anti-IFNγ (BioLegend, 505836), FITC-anti-GzmB (BioLegend, 372206), PE-anti-perforin (BioLegend, 154305) and BV605-anti-TNF (BioLegend, 506329). The samples were analysed using a BD LRS Fortessa ×20 flow cytometer. The analysis of flow cytometry data was conducted using FlowJo software.

DNA laddering assay

Cell DNA was extracted and purified with DNA extraction kit (Beyotime, C0003) according to the manufacturer's instructions. Purified DNA was subjected to agarose gel electrophoresis.

Annexin V/PI staining

Cells were washed twice with PBS and stained using an Annexin V-FITC/PI apoptosis detection kit (eBioscience, 88-8005-74) according to the manufacturer's instructions. The stained cells were analyzed using a BD Fortessa flow cytometer.

Cellular thermal shift assay (CETSA)

HEK293T cells were transfected with CypD-Flag, and CypD proteins were immunoprecipitated using an anti-Flag antibody together with protein A/G agarose beads, followed by elution with 3× Flag peptide. The eluents were incubated with 20 μM DHN or DMSO at 4 °C for 2 hours, then divided into 60 μL aliquots and individually heated at various temperatures (40, 45, 50, 55, 60, 65, 70, 75, 80 °C) for 15 minutes. After centrifugation at 20,000× g at 4 °C for 20 minutes to separate soluble fractions from precipitates, the supernatants were analyzed by SDS-PAGE followed by western blot analysis.

Assessment of mPTP Opening by Calcein Loading/$CoCl_2$ Quenching

We performed calcein-AM loading/$CoCl_2$ quenching assay to evaluate mPTP opening. Briefly, cells were treated with 1 μM calcein-AM in darkness at 37 °C for 15 min in the presence of 5 mM cobalt chloride ($CoCl_2$) to quench the cytoplasmic and nuclear signals. Calcein was excited at 494 nm, and emission was recorded at 517 nm. Fluorescence intensity emitted by the cells was assessed using a spectrometer to assess of mPTP Opening.

Cellular fractionation and measurement of cytosolic mtDNA

Cell fractionation was performed according to previously described methods (Journal of immunology (Baltimore, Md : 1950). 1999;163(9):4683-93.). In brief, cells were scraped from the culture dish, collected in ice-cold PBS, and washed twice with ice-cold PBS. The cells were then resuspended in 200 μL of digitonin lysis buffer (75

mM NaCl, 1 mM NaH_2PO_4, 8 mM Na_2HPO_4, 250 mM sucrose, and 190 mg/mL digitonin). Digitonin permeabilizes the cell membrane. Vigorous shaking was avoided to prevent mitochondrial damage. After incubation on ice for 5 minutes, the cells were centrifuged at 12,000× g for 5 minutes at 4°C. The supernatant and pellet were separately collected: the supernatant represented the cytoplasmic fraction devoid of mitochondria, while the pellet contained the mitochondrial fraction. Both fractions were used for subsequent qPCR and Western blot (WB) analyses.

qPCR was used to quantify total cytosolic mtDNA. Genomic DNA was extracted using the MiniBEST Universal Genomic DNA Extraction Kit Ver.5.0 (Cat# 9765, TaKaRa), and mitochondrial DNA content was assessed by RT-PCR, normalized to the nuclear gene RPL13A. The following primers were used (5'-3'):

MT-ND1: forward, CACCCAAGAACAGGGTTTGT

reverse, TGGCCATGGGTATGTTGTTAA

MT-CO2: forward, AATCGAGTAGTACTCCCGATTG

reverse, TTCTAGGACGATGGGCATGAAA

MT-ATP6: forward, AATCCAAGCCTACGTTTTCACA

reverse, AGTATGAGGAGCGTTATGGAGT

RPL13A: forward, GCCCTACGACAAGAAAAAGCG

reverse, TACTTCCAGCCAACCTCGTGA

To detect the length of cytosolic mtDNA, purified mtDNA was subjected to agarose gel electrophoresis. For the measurement of Ox-mtDNA, purified mtDNA was extracted from the cytosolic as indicated. The 8-OH-dG content was then quantified

using 8-hydroxy 2-deoxyguanosine ELISA Kit (Cat# D751009-0048, Sangon Biotech), per manufacturer's instructions.

PCR was used to detect specific cytosolic mtDNA fragments (~600 bp and ~5000 bp). Purified total and cytosol DNA was amplified using ApexHF HS DNA Polymerase FS (ACCURATE BIOTECHNOLOGY(HUNAN) CO., LTD, Changsha, China, AG12202), containing 250 nM of each primer. 5304 bp and D-loop 623 bp PCR was carried out using 5 ng DNA extracted from total cells or 50 ng DNA extracted from cytoplasm as the template. The following PCR conditions were used. Hot start at 98 ℃ for 10 minutes, melting temperature of 98 ℃ for 10 seconds, annealing temperature of 56 ℃ (for 5304 bp and 623 bp) for 30 seconds and extension temperature of 72 ℃ for 30 seconds (623 bp) or 5 minutes (5304 bp), with 30 (623 bp) or 40 (5304 bp) cycles, followed by 72 ℃ for 10 minutes and kept at 16 ℃. Amplified products were analyzed on 1 % agarose gels stained with ethidium bromide. The following primers were used (5'-3'):

D-loop-623 bp: forward, ATTACTGCCAGCCACCATGAATATT

reverse, ATAGGATGAGGCAGGAATCAAAGACA

mtDNA-5304 bp: forward, GAGTAAATAATAGGAGCTTAAACCCCCT

reverse, GGAGTGGGACTTCTAGGGGATTTAG

Preparation of the TI fraction

Cells were lysed using pre-chilled Triton X-100 lysis buffer (50 mM Tris-HCl, pH 7.5, 150 mM NaCl, 1% Triton X-100, 1 mM PMSF, and a protease cocktail).

Subsequently, the lysates were incubated on ice for 15 minutes and then centrifuged at 15,000× g at 4 °C for 30 minutes to isolate the TI precipitates. The precipitates were washed three times with lysis buffer and subsequently treated with lysis buffer containing 1% SDS at 60 °C for 45 minutes in a water bath. Following this step, the TI precipitates were sonicated for western blot analysis whereas total proteins were directly extracted using Triton X-100 lysis buffer containing 1% SDS.

TurboID assay

TurboID assay was performed according to previously described methods (Cell Res. 2023;33(12):904-22; Nature biotechnology. 2018;36(9):880-7). Briefly, culture medium was switched to serum-free medium for 0.5 hours, followed by the addition of 100 μM biotin for 10 minutes incubation at 37 °C. Subsequently, the medium was replaced with 10% FBS-containing medium for a 2 hours incubation period, and biotin-labeled proteins were purified using streptavidin magnetic beads (Cat# 88816, Thermo Fisher Scientific). Finally, the samples were prepared for western blot analysis.

Endoplasmic reticulum-IP

Three million STING-HA overexpressing cells were plated in 15-cm plate and treated with 20 μM DHN for 12 hours. Cells were washed with KPBS (136 mM KCl, 10 mM KH_2PO_4, pH 7.25 adjusted with KOH), pelleted by centrifugation at 2,000× g at 4 °C for 1 minute, and resuspended in 1 mL of ice-cold KPBS, with 50 μL of this suspension collected as the whole-cell control. The cell suspension was homogenized

in a 7 mL Dounce homogenizer (Cat. P0610, Sigma) on ice for 60 strokes, followed by centrifugation at 1,000× g at 4°C for 10 minutes. The supernatant was centrifuged at 20,000× g for 20 minutes, and the resulting pellet was gently resuspended in ice-cold KPBS. This suspension was then mixed with 100 μL of KPBS-washed anti-HA magnetic beads (Cat# 88836, Thermo Fisher Scientific) and incubated at 4 °C for 5 minutes. After washing the beads, whole-cell and immunoprecipitated samples were analyzed by western blot.

Transmission electron microscopy

Cells were fixed in ice-cold 2.5% glutaraldehyde in 0.1 M phosphate buffer (pH 7.4) at 4 °C for 3 hours, then washed and post-fixed in 1% OsO_4 in 0.1 M PBS (pH 7.4) at 4 °C for 2 hours. Samples were dehydrated in an ethanol gradient (30%, 50%, 70%, 90%, and 100% ethanol) and embedded in Spurr resin. Ultrathin sections (60 nm) were prepared, stained, and examined under a JEM2100HC transmission electron microscope (Hitachi).

APEX assay

APEX assay was performed according to previously described methods (Nature protocols. 2017;12(9):1792-816.). Briefly, the STING-APEX-expressing cells were seeded onto 6-cm plate overnight and fixed in ice-cold 2.5% glutaraldehyde in 0.1 M phosphate buffer (pH 7.4) at 4°C for 3 hours. Fixed cells were then treated with a freshly

prepared solution containing 0.5 mg/ml diaminobenzidine and 10 mM hydrogen peroxide in cacodylate buffer at room temperature for 45 minutes in a darkroom. Ultrathin sections were meticulously prepared, stained, and examined under a JEM2100HC transmission electron microscope (Hitachi).

Measuring intracellular pH value

The intracellular pH value of live cells was measured using the pHrodo™ Green AM intracellular pH indicator, a fluorogenic probe, along with the intracellular pH calibration buffer kit, following the manufacturer's protocol (Cat# P35373, Thermo Fisher Scientific) (Cell Res. 2018;28(10):996-1012.). Briefly, cells were washed with live cell imaging solution (LCIS) and then incubated at 37 °C for 30 minutes with a mixture of 10 μL pHrodo™ Green AM, 100 μL PowerLoad™ and 10 mL LCIS, followed by pH measurement using a spectrometer. Subsequently, cells were exposed to lactic acid, phloretin, or lonidamine in DMEM without phenol red as needed. Fluorescence intensity emitted by the cells was assessed using a spectrometer to analyze their intracellular pH value.

pH measurement of endoplasmic reticulum

The pH value of the endoplasmic reticulum (ER) was measured following previously described methods (Scientific reports. 2018;8(1):11985; Microbiology (Reading, England). 2009;155(Pt 1):268-78). Briefly, to generate calibration curves, cells expressing ER-sfpHluorin plasmid were resuspended in ice-cold PBS containing

100 μg/mL digitonin for 10 minutes. Subsequently, the cells were washed with PBS and added to a citric acid/Na_2HPO_4 buffer with pH values ranging from 5.0 to 8.5. Excitation spectra at different pH values were recorded using the Tecan Infinite E plex system (λem = 508 nm; λes = 350 to 490 nm). To determine ER-pH, fluorescence emission was measured at 508 nm with excitation wavelengths of 390 nm and 470 nm. ER-pH value was calculated based on the ratio of fluorescence emission (390/470 nm) corresponding to the calibration curves.

STING *in vitro* kinase assays

HEK293T cells were transfected with $STING^{WT}$-Flag or $STING^{S345A/S358A}$, followed by immunoprecipitation of the STING protein using anti-Flag antibody together with protein A/G agarose beads. The elution was performed with 3× Flag peptide. Subsequently, bacterially purified recombinant $PERK^{536\text{-}1116}$ kinase-GST (0.5 μg) was incubated with 10 μL immunoprecipitated STING in 40 μL of kinase reaction buffer (50 mM Tris-HCl (pH 7.5), 1 μM $MnCl_2$, 250 μM ATP-γ-S) at 37 °C for 1 hour, without or with 10 μM cGAMP. Enzymatic reactions were then stopped by 20 mM EDTA, and 5 mM p-Nitrobenzyl mesylate (PNBM) was added for 1 hour at room temperature to induce thiol residues alkylation. Reactions were stopped by 2× SDS loading buffer and submitted to immunoblotting using an anti-thiophosphate ester antibody.

Immunohistochemical staining

The paraffin-embedded tumor tissue was cut into 5μm sections using a microtome and rehydrated. The sections were then microwaved in the 10 mM sodium citrate buffer (pH 6.0) for 10 minutes. Subsequently, sections were blocked at room temperature for 1 hour with 10% normal goat serum or kit of mouse-on-mouse immunodetection, and then incubated with Ki67 primary antibody at 4 °C overnight. Finally, peroxidase labeled polymers and substrate chromogens were used for protein staining.

The tumor tissues were embedded with optimal cutting temperature compound and cut into 7 μm sections using a cryotome, then fixed with acetone. The sections were blocked at room temperature for 1 hour with blocking buffer (10% donkey serum, 0.5% BSA and 0.5% Triton X-100) and then incubated with STING primary antibody at 4 °C overnight. Followed by, the sections were incubated with FITC or Texas Red-conjugated secondary antibodies (Life Technologies) for one hour at 37 °C. Nuclei were visualized by staining with 4′,6-diamidino-2-phenylindole (DAPI) at a concentration of 50 μg/mL for five minutes. Confocal images were acquired using a Zeiss LSM 980 confocal microscope.

***In vivo* pharmacokinetic and pharmacodynamic analysis**

Mice received a single intraperitoneal dose of DHN (20 mg/kg). Blood samples were collected at 15 min, 30 min, 1 h, 2 h, 4 h, 6 h, 8 h, 24 h, 32 h, and 48 h post-administration. Plasma was isolated by centrifuging whole blood at 1,500 × g for 10 min. DHN concentrations in plasma were quantified using liquid chromatography-tandem mass spectrometry (LC-MS/MS). Pharmacokinetic parameters were calculated

with Phoenix WinNonlin® 7.0 (ZT-1a module). All plasma DHN measurements were performed by Truway Biotechnology (Suzhou, China).

For tumor-implanted mice, samples were obtained following the final DHN treatment. Blood and tumor tissues were collected at 1 h, 2 h, 4 h, 8 h, and 24 h. DHN levels in whole blood and tumor homogenates were determined by Truway Biotechnology (Suzhou, China). For pharmacodynamic assessment, tumor tissues were homogenized and lysed to extract proteins. Western blotting detected GSDME-N-terminal cleavage fragments, with band intensities quantified as the GSDME-N/Tubulin ratio.

Chemical Labelling of DHN-P

Cells were lysed in ELB lysis buffer containing a protease inhibitor cocktail and phosphatase inhibitor cocktail, followed by centrifugation at 14,000× g at 4 °C for 15 min. Subsequently, the supernatants were incubated with 20 μM DHN probes (DHN-P) at 4 °C for 2 hours, followed by exposure to UV light at a wavelength of 365 nm for 20 minutes. The supernatants were adjusted to final concentration of 1 mM TCEP (Cat# T10252, Aladdin), 0.1 mM TBTA (Cat# T162437, Aladdin), 1 mM $CuSO_4$, and 1 mM Biotin-azide (Cat# HY-129832, MedChemExpress) linker and incubated at 4 °C for one hour. Protein aggregates were removed through centrifugation at 20,000× g for fifteen minutes. NeutrAvidin beads (diluted to a ratio of 1:100) were then added to the lysates and incubated with gentle rotation for two hours. Finally, the beads were washed three times using an ELB lysis buffer volume that was one hundred times

greater than their own volume before being mixed with an equal volume of 2× SDS loading buffer for western blotting.

For fluorescence localization of DHN-P, cells were treated with 20 μM DHN-P, Neg-P, or DMSO for 1 hour, followed by UV irradiation (365 nm) for 20 minutes. After fixation with 4% PFA for 15 minutes and permeabilization with 0.1% Triton X-100 in PBS for 5 minutes, cells were washed three times with a solution of 1% BSA/PBS. Subsequently, samples were incubated in the dark at room temperature for 30 minutes with a click reaction cocktail consisting of 1 μM rhodamine-N3 (Cat# F278701, Aladdin), 1 μM TBTA, 10 μM TCEP and 10 μM $CuSO_4$. This was followed by three washes with PBS before confocal microscopy was performed to capture images using a Zeiss LSM 780 confocal microscope.

Synthesis of DHN-P and Neg-P

Reagents and solvents were purchased from chemical suppliers and used unless specified. 1H nuclear magnetic resonance (NMR) and 13C NMR spectra were obtained on a BrukerUltrashield Plus-600 (600-MHz) spectrometer. Chemical shifts are reported in parts per million (δ) relative to residual undeuterated solvent as an internal reference. Coupling constants (J) are reported in hertz. Spin multiplicities are described as s (singlet), d (doublet), dd (double-doublet), dt (double-triplet), t (triplet), q (quartet), and m (multiplet). High resolution mass spectra (HRMS) were obtained on Thermo Fisher Q-exactive combined quadrupole Orbitrap mass spectrometer. Mass spectra were recorded on Waters 3100 Mass Detector.Reactions were monitored by thin-layer

chromatography and were visualized with UV light. Removal of solvents was conducted by using a rotary evaporator, and residual solvent was removed from nonvolatile compounds using a vacuum manifold maintained at 1 torr. Chromatography was performed on ISCO CombiFlashRf 200 with prepacked silica-gel cartridges (RediSepRf Gold High Performance). Preparative high-pressure liquid chromatography was performed on a Waters Symmetry C18 column (19× 50 mm, 5 μM) using a gradient of 5%–95% methanol in water containing 0.1% trifluoacetic acid over 8 min (10-min run time) at a flow rate of 20 mL/min.

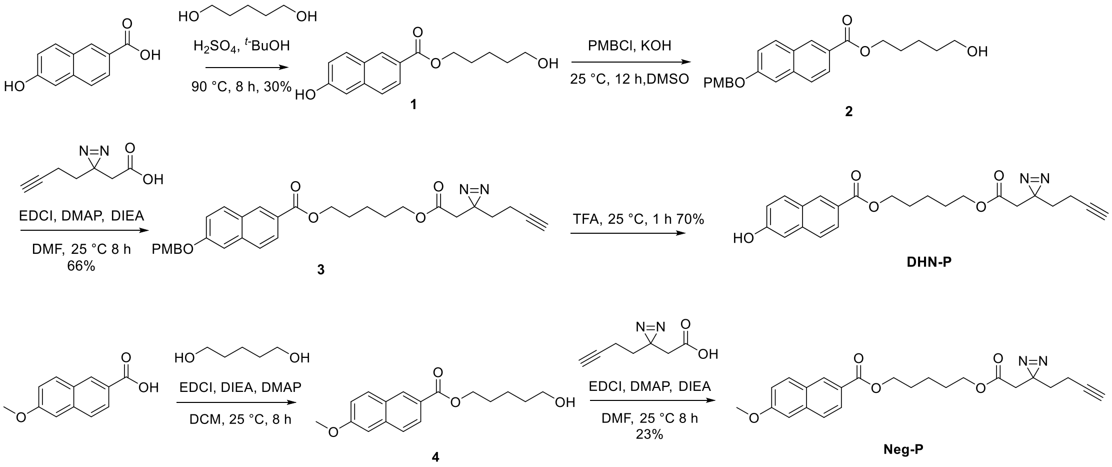

To a stirred mixture of 6-hydroxy-2-naphthoic acid (564.0 mg, 3.0 mmol) and 1,5-pentanediol (1.56 g, 15.0 mmol) in t-BuOH (10.0 mL) was added concentrated sulfuric acid (1.0 mL) at 0 °C, then the reaction mixture was heated at 90 °C for 8 h until the reaction was completed. The reaction product was purified by silica gel column chromatography to afford compound 1 (247.0 mg, 30% yield) as a white solid. To a stirred solution of 1 (247 mg, 0.9 mmol) in dimethyl sulfoxide (DMSO) (1.0 mL) was sequentially added KOH (90 mg, 2.25 mmol) and 4-methoxybenzyl chloride (172 mg, 1.1 mmol). The resulting mixture was stirred at 25 °C for 12 h until the reaction was

completed. Then the reaction mixture was dried, filtered and concentrated to give crude product 2 as a white solid (261 mg). The crude product 2 was used directly in the next step without further purification. To a stirred solution of 1-ethyl-(3-dimethylaminopropyl)carbodiimide hydrochloride (EDCI) (38.4 mg, 0.2 mmol), N,N-diisoproylethylamine (DIEA) (25.8 mg, 0.2 mmol), 4-dimethylaminopyridine (DMAP) (1.22 mg, 0.01 mmol) and 2-(3-(but-3-yn-1-yl)-3H-diazirin-3-yl)acetic acid (19 mg, 0.12 mmol) in dimethylacetamide (DMF) (1 mL) was dropwisely added the solution of compound 2 (39.4 mg in 1 mL of DMF) at room temperature for 12 h until the reaction was completed. The reaction product was purified by silica gel column chromatography to afford compound 3 (34.8 mg, 66% yield) as a faint yellow solid. To a solution of 3 (10 mg, 0.019 mmol) in dichloromethane (0.2 mL) was added trifluoroacetic acid (200 μL) at 0 °C. The mixture was stirred for 5 min, then warmed up to room temperature and stirred for additional 1 h. After the reaction was completed, the solvent was removed under reduced pressure and the residue was purified by preparative high-pressure liquid chromatography using a gradient of 5%–95% methanol in water containing 0.1% trifluoacetic acid to afford probe DHN-P as dark red solid (7.1 mg, 90% yield).

DHN-P, 1H NMR (600 MHz, Chloroform-d): δ 8.52 (s, 1H), 8.00 (dd, J = 8.6, 1.7 Hz, 1H), 7.87 (d, J = 8.7 Hz, 1H), 7.70 (d, J = 8.6 Hz, 1H), 7.21 – 7.13 (m, 2H), 4.38 (t, J = 6.5 Hz, 2H), 4.17 (t, J = 6.6 Hz, 2H), 3.50 (s, 1H), 2.33 (s, 2H), 2.03 (dt, J = 7.4, 2.6 Hz, 2H), 1.99 (t, J = 2.6 Hz, 1H), 1.89 – 1.80 (m, 2H), 1.76 (dt, J = 7.2, 3.9 Hz, 4H),

1.60 – 1.55 (m, 2H). 13C NMR (151 MHz, Chloroform-d): δ 169.23, 166.97, 155.63, 137.13, 131.50, 130.99, 127.82, 126.50, 125.98, 125.35, 118.71, 109.50, 82.48, 69.44, 65.04, 64.72, 39.73, 32.05, 29.73, 28.44, 28.26, 25.54, 22.61, 13.22. MS (ESI) m/z: 409 [M + H] +, HRMS (ESI+): calcd for C23H24N2O5 [M+Na] + 431.1583, found 431.1570.

5-(2-(3-(but-3-yn-1-yl)-3H-diazirin-3-yl)acetoxy)pentyl 6-methoxy-2-naphthoate (Neg-P) was prepared from 6-methoxy-2-naphthoic acid following the synthetic procedure of DHN-P. Neg-P was obtained as faint yellow solid (20 mg, 23% yield).

Neg-P, 1H NMR (600 MHz, Chloroform-d): δ 8.51 (s, 1H), 8.01 (dd, J = 8.6, 1.7 Hz, 1H), 7.84 (d, J = 9.0 Hz, 1H), 7.75 (d, J = 8.6 Hz, 1H), 7.19 (dd, J = 8.9, 2.5 Hz, 1H), 7.15 (d, J = 2.5 Hz, 1H), 4.38 (t, J = 6.5 Hz, 2H), 4.16 (t, J = 6.6 Hz, 2H), 3.93 (s, 3H), 2.32 (s, 2H), 2.05 (s, 1H), 2.02 (dd, J = 7.3, 2.7 Hz, 2H), 1.87 – 1.82 (m, 2H), 1.75 (dt, J = 7.3, 4.5 Hz, 4H), 1.61 – 1.51 (m, 2H). 13C NMR (151 MHz, Chloroform-d): δ 169.12, 166.90, 159.55, 137.16, 130.88, 130.76, 127.90, 126.84, 125.92, 125.39, 119.65, 105.69, 82.47, 69.42, 64.98, 64.64, 55.40, 39.70, 32.08, 28.45, 28.26, 25.50, 22.60, 13.20. MS (ESI) m/z: 423 [M + H] +, HRMS (ESI+): calcd for C24H26N2O5 [M+Na] + 445.1739, found 445.1725.

Synthesis of DHN

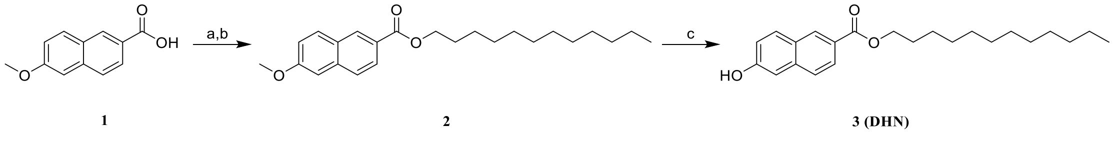

Reagents and conditions: (a) HOBT, EDCI, DCM, 0 °C, 1 h; (b) 1-Dodecanol, Et3N, 50 °C, 5 h, 84%, for 2 steps; (c) BBr3, DCM, -5 °C, 2 h, 76%.

Synthesizing Dodecyl 6-hydroxy-2-naphthoate (3). A solution of 6-methoxy-2-naphthoic acid (404 mg, 2.0 mmol) and HOBT (297 mg, 2.2 mmol) in anhydrous DCM (10 mL) was added EDCI (421.7 mg, 2.2 mmol) under ice bath conditions, stirred thoroughly for 1 h, then added 1-dodecanol (445 μL, 2.0 mmol), Et3N (306 μL, 2.2 mmol), heated to 50 °C and stirred for 5 h. After confirming the completion of the reaction with TLC, the reaction was quenched by aq. NaHCO3 and brine, dried over Na2SO4 and concentrated in vacuo. The obtained liquid was purified by column chromatography with petroleum ether and ethyl acetate to provide 2 (622 mg, 84%, 2 steps) as a white solid. To a solution of 2 (370 mg, 1.0 mmol) in DCM (6 mL), vacuum, argon gas protection, added BBr3 (2.5 mL, 10.0 mmol) at -5 °C, stirred for 2 h. The mixture was poured into water and extracted with ethyl acetate. A diluted solution was washed water (2× 30 mL) and brine (3× 30 mL), and then dried with Na2SO4 and concentrated in vacuo. A residue was purified with column chromatography with petroleum ether and ethyl acetate (PE / EA = 30 : 1) to provide 3 (271 mg, 76%) as a white solid. Mp = 92-94 °C.

DHN, 1H NMR (600 MHz, CDCl3) δ 8.51 (s, 1H), 7.99 (dd, J = 8.6, 1.6 Hz, 1H), 7.85 (d, J = 8.7 Hz, 1H), 7.68 (d, J = 8.6 Hz, 1H), 7.16 (d, J = 2.0 Hz, 1H), 7.14 (dd, J = 8.8, 2.4 Hz, 1H), 5.49 (br s, 1H), 4.34 (t, J = 6.7 Hz, 2H), 1.83 – 1.75 (m, 2H), 1.49 – 1.41 (m, 2H), 1.40 – 1.32 (m, 2H), 1.26 (dd, J = 17.6, 9.5 Hz, 14H), 0.85 (t, J = 7.0 Hz, 3H). 13C NMR (150 MHz, CDCl3) δ 167.11 , 155.60 , 137.08 , 131.46 , 130.94 , 127.81 ,

126.43 , 126.01 , 125.51 , 118.65 , 109.46 , 65.24 , 31.92 , 31.50 , 30.12 , 29.64 , 29.64 , 29.35 , 29.35 , 28.78 , 26.08 , 22.69 , 14.13 . HRMS (ESI): m/z calcd for C23H32O3 [M-H]- 355.2273, found 355.2271.

Supplementary Figure

Supplementary Figure 1

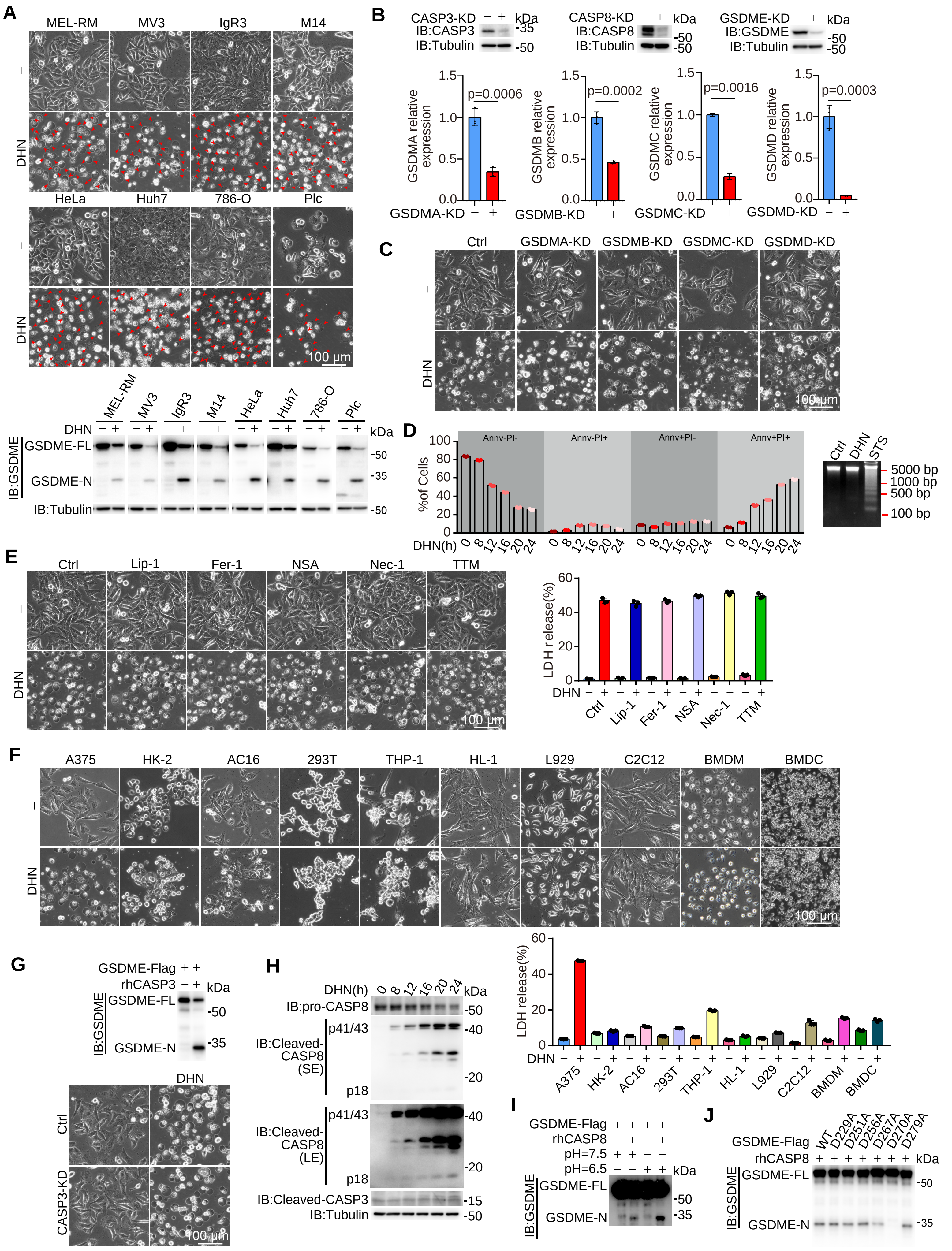

Supplementary Figure 1

Fig. S1A DHN induces pyroptosis through GSDME mediation in different types of cancer cell lines. Different cancer cell lines as indicated were treated with DHN (15 μM) for 20 hours. Pyroptosis was detected with morphology (red arrows indicate pyroptosis cells) and GSDME cleavage.

Fig. S1B Knock down efficiencies of different genes as indicated in A375 cells, detected by real-time PCR or western blotting.

Fig. S1C Knocking down GSDMA, GSDMB, GSDMC or GSDMD cannot affect DHN-induced pyroptosis. These genes were separately knocked down in A375 cells first, and the cells were treated with DHN (15 μM) for 20 hours, the pyroptotic morphology was then determined.

Fig. S1D DHN cannot induce cells to produce DNA laddering and Annexin V^{+}/PI^{-} cells. A375 cells were treated with DHN (15 μM) for 20 hours, purified DNA was subjected to agarose gel electrophoresis (left). A375 cells were treated with DHN (15 μM) for different time, stained using an Annexin V-FITC/PI apoptosis detection kit (right).

Fig. S1E DHN-induced death cannot be inhibited by inhibitors other than pyroptosis. A375 cells were co-treated with DHN and various inhibitors (lip-1: 10 μM, fer-1: 10 μM, NSA: 10 μM, Nec-1: 10 μM, TTM: 10 μM) for 20 hours, pyroptotic morphology and LDH release were determined.

Fig. S1F DHN can hardly induce pyroptosis in non-cancer cell lines. Different non-tumor cell lines as indicated were treated with DHN (15 μM) for 20 hours. Pyroptosis was detected with morphology and LDH release.

Fig. S1G Caspase-3 cleaves GSDME, but fails to impair DHN-induced pyroptosis. Recombinant human caspase-3 protein was incubated with GSDME purified from HEK293T cells, the cleavage of GSDME was detected *in vitro* (top). Caspase-3 was knocked down in A375 cells and then treated with DHN (15 μM) for 20 hours, pyroptotic morphology was determined (bottom).

Fig. S1H DHN promotes the cleavages of caspase-8 and caspase-3. A375 cells were treated with DHN (15 μM) for indicated times, the cleaved-caspase-8 and -caspase-3 were assayed.

Fig. S1I Caspase-8 cleaves GSDME *in vitro*. Recombinant human caspase-8 protein was incubated with GSDME purified from HEK293T cells in cleavage buffers with different pH, the cleavage of GSDME was detected.

Fig. S1J Determination of critical site in GSDME for Caspase-8 cleavage in the *in vitro* case. Different mutants in GSDME were constructed as indicated. Recombinant human caspase-8 protein was incubated with GSDME purified from HEK293T cells that expressed different GSDME mutants, the cleavage of GSDME was detected.

Tubulin was used to determine the amount of loading proteins. Statistical analyses were determined by unpaired two-tailed Student's t-test (**S1B**). P values are indicated. All western blots were repeated at least twice and one of them is shown.

Supplementary Figure 2

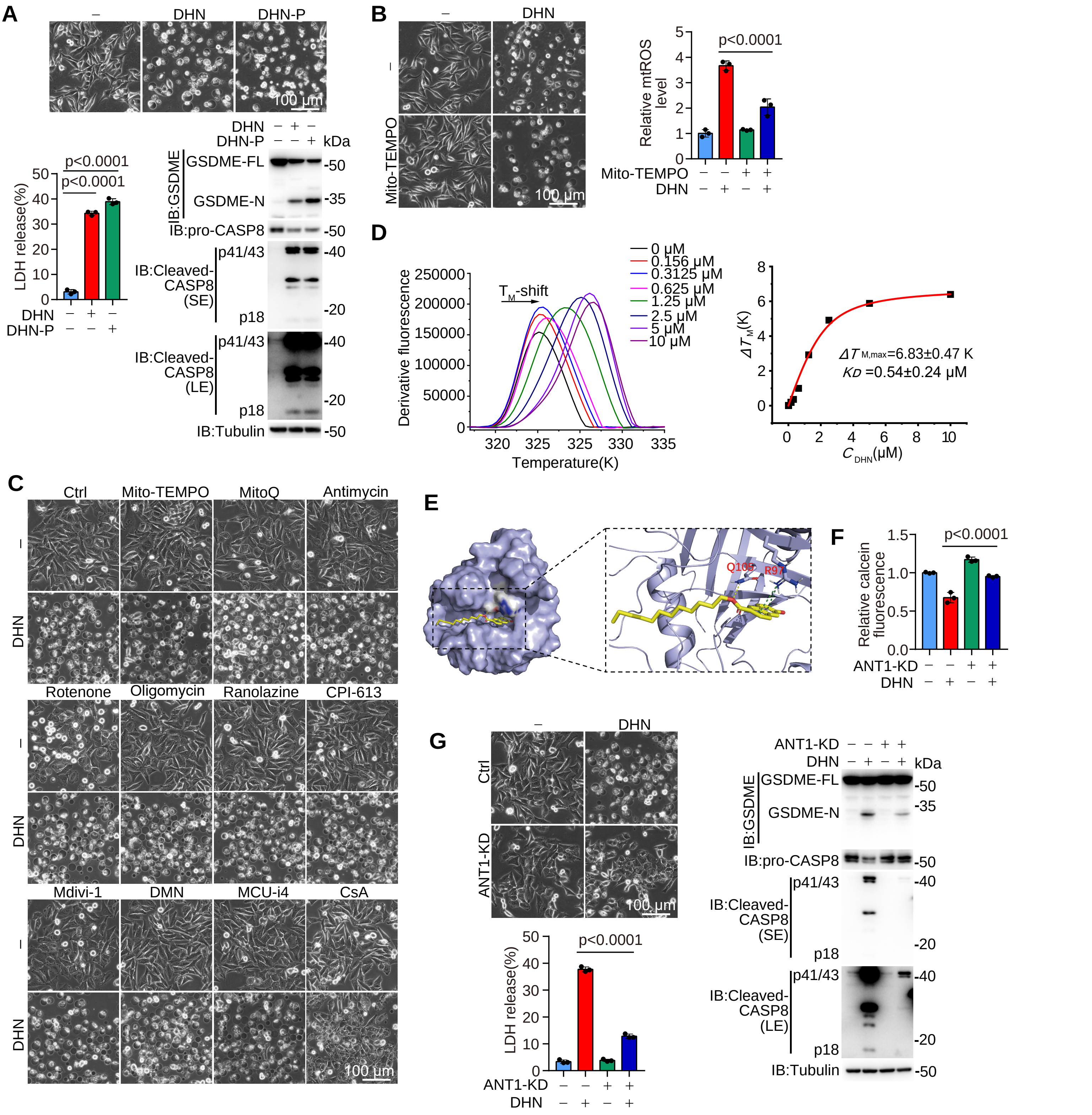

Supplementary Figure 2

Fig. S2A Similar to DHN, DHN-P has the capacity to induce pyroptosis. Cells were treated with DHN-P (150 μM) for 20 hours and pyroptosis was assayed.

Fig. S2B mtROS shows no effect on DHN-induced pyroptosis. A375 cells were co-treated with Mito-TEMPO (10 μM) and DHN (15 μM) for 12 hours, mtROS (right) and pyroptosis (left) were detected.

Fig. S2C CsA inhibits DHN-induced pyroptosis. A375 cells were co-treated with DHN and various inhibitors (Antimycin A: 0.1 μM, Rotenone: 0.1 μM, Oligomycin: 0.1 μM, CPI613: 10 μM, DMN: 8 μM, Mdivi-1: 10 μM, MitoQ: 2 μM, Mito-TEMPO: 10 μM, CsA: 5 μM, MCU-i4: 5 μM, Ranolazine: 10 μM) for 20 hours, pyroptotic morphology was determined.

Fig. S2D The binding affinity between DHN and CypD was determined by FL-DSF. The FL-DSF assay yielded a *Kd* value of 0.69 ± 0.33 μM

Fig. S2E Docking analysis suggests the possible sites of DHN binding to CypD. Molecular docking indicates the theoretical binding mode of DHN to CypD (PDB: 5CBV), in which the naphthalene ring of DHN forms a distinct cationic π-interaction with R97 of CypD (dotted green line), and Q105 forms a hydrogen bond with the oxygen atom (dotted yellow line).

Fig. S2F Knocking down ANT1 suppresses the mPTP opening. The ANT1-knockdown cells were treated with DHN (15 μM) for 12 hours, and the mPTP opening was assayed.

Fig. S2G Knocking down ANT1 suppresses pyroptosis. The ANT1-knockdown cells were treated with DHN (15 μM) for 20 hours, and pyroptosis was assayed

Tubulin was used to determine the amount of loading proteins. Statistical analyses were determined by one-way ANOVA with Tukey's multiple comparisons test (**S2A**) and two-way analysis of variance (ANOVA) with Tukey's multiple comparisons test (**S2F, S2G**). P values are indicated. All western blots were repeated at least twice and one of them is shown.

Supplementary Figure 3

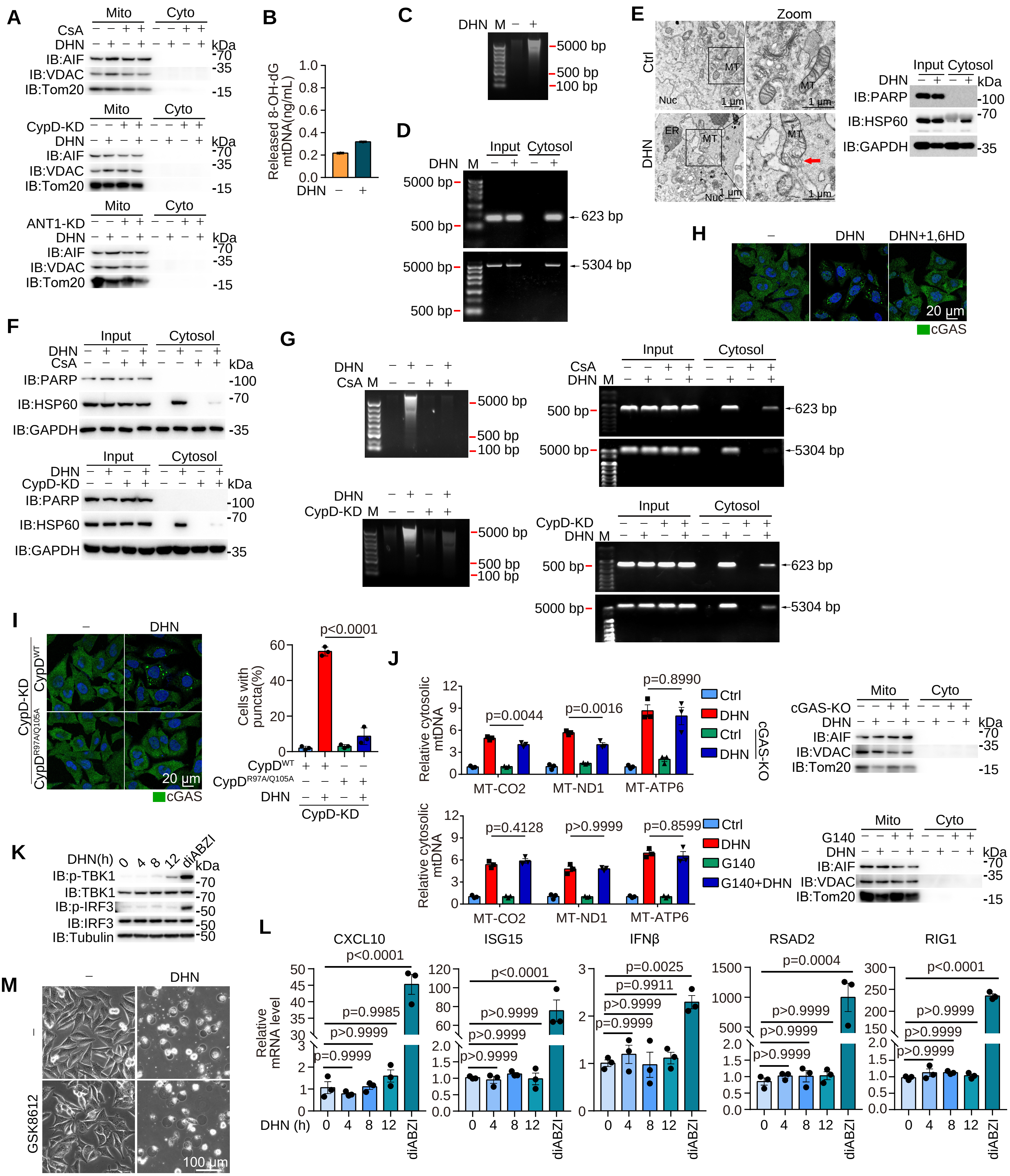

Supplementary Figure 3

Fig. S3A The mitochondrial and cytoplasmic components were isolated. The cells were co-treated with CsA (5 μM, top) and DHN (15 μM) for 12 hours, or the cells with CypD (middle) or ANT1 (bottom) knockdown were individually subjected to DHN treatment, and isolated the cytoplasm. AIF, VDAC and Tom20 were detected by western blot.

Fig. S3B DHN cannot induce the oxidation of mtDNA. A375 cells were treated with DHN (15 μM) for 12 hours and the oxidation of mtDNA was detected.

Fig. S3C DHN induces 623 bp and 5304 bp mtDNA into the cytoplasm. A375 cells were treated with DHN (15 μM) for 12 hours,5304 bp and D-loop 623 bp cytosolic mtDNA fragment were detected.

Fig. S3D DHN induces the presence of fragmented DNA in the cytoplasm. A375 cells were treated with DHN (15 μM) for 12 hours, the length of cytosolic mtDNA was detected.

Fig. S3E DHN induces mitochondrial rupture. A375 cells were treated with DHN (15 μM) for 12 hours. The mitochondrial structure was observed under electron microscope (left). The cytoplasmic Hsp60 was detected by WB (right).

Fig. S3F CypD knockdown or CsA treatment can inhibit mitochondrial rupture induced by DHN. A375 cells were co-treated of CsA (10 μM) with DHN (15 μM) for 12 hours, or CypD was knocked down in A375 cells first and then cells were treated with DHN (15 μM) for 12 hours. The cytoplasmic Hsp60 wasdetected.

Fig. S3G CypD knockdown or CsA treatment can inhibit the release of mtDNA into the cytoplasm induced by DHN. A375 cells were co-treated of CsA (10 μM) with DHN

(15 μM) for 12 hours, or CypD was knocked down in A375 cells first and then cells were treated with DHN (15 μM) for 12 hours. The length of cytosolic mtDNA (left) and 5304 bp or D-loop 623 bp cytosolic mtDNA fragment (right) were detected.

Fig. S3H DHN induces liquid-liquid phase separation of cGAS. A375 cells were treated with DHN (15 μM) for 12 hours and incubated with 1,6-HD (2.5%) for 5 mins. Anti-cGAS antibody was used to indicate the cGAS puncta observed under confocal microscope.

Fig. S3I The R97A/Q105A mutation of CypD suppresses the formation of cGAS puncta. $CypD^{WT}$ or its mutants $CypD^{R97A/Q105A}$ were separately transfected into CypD-knockdown A375 cells, treated with DHN (15 μM) for 12 hours, and then stained with cGAS antibody. cGAS puncta were observed under confocal microscope (left) and the percentage of cells with cGAS puncta was quantified (right).

Fig. S3J Knocking out cGAS or G140 treatment shows no effect on the release of mtDNA induced by DHN. The cGAS-knockout cells were treated with DHN or cells were co-treated with G140 (30 μM) and DHN, followed by detection of mtDNA release.

Fig. S3K DHN rarely activates TBK1 and IRF3. The cells were treated with DHN (15 μM, 12 h) or diABZI (10 μM, 12 h) and the phosphorylation levels of TBK1 and IRF3 were detected.

Fig. S3L DHN cannot affect mRNA expression levels of CXCL10, ISG15, IFNβ, RSAD2 and RIG1. A375 cells were treated with DHN (15 μM) for indicated time, the mRNA expression levels of CXCL10, ISG15, IFNβ, RSAD2 and RIG1 were detected by real-time PCR. diABZI (10 μM, 12 h) was used as a positive control.

Fig. S3M GSK8612 treatment cannot impair DHN-induced pyroptosis. The cells were co-treated with GSK8612 (10 μM) and DHN (15 μM) for 20 hours, followed by the detection of pyroptosis.

Tubulin was used to determine the amount of loading proteins. Statistical analyses were determined by two-way analysis of variance (ANOVA) with Tukey's multiple comparisons test (**S3I, S3J**) and one-way ANOVA with Tukey's multiple comparisons test (**S3L**). P values are indicated. All western blots were repeated at least twice and one of them is shown.

Supplementary Figure 4

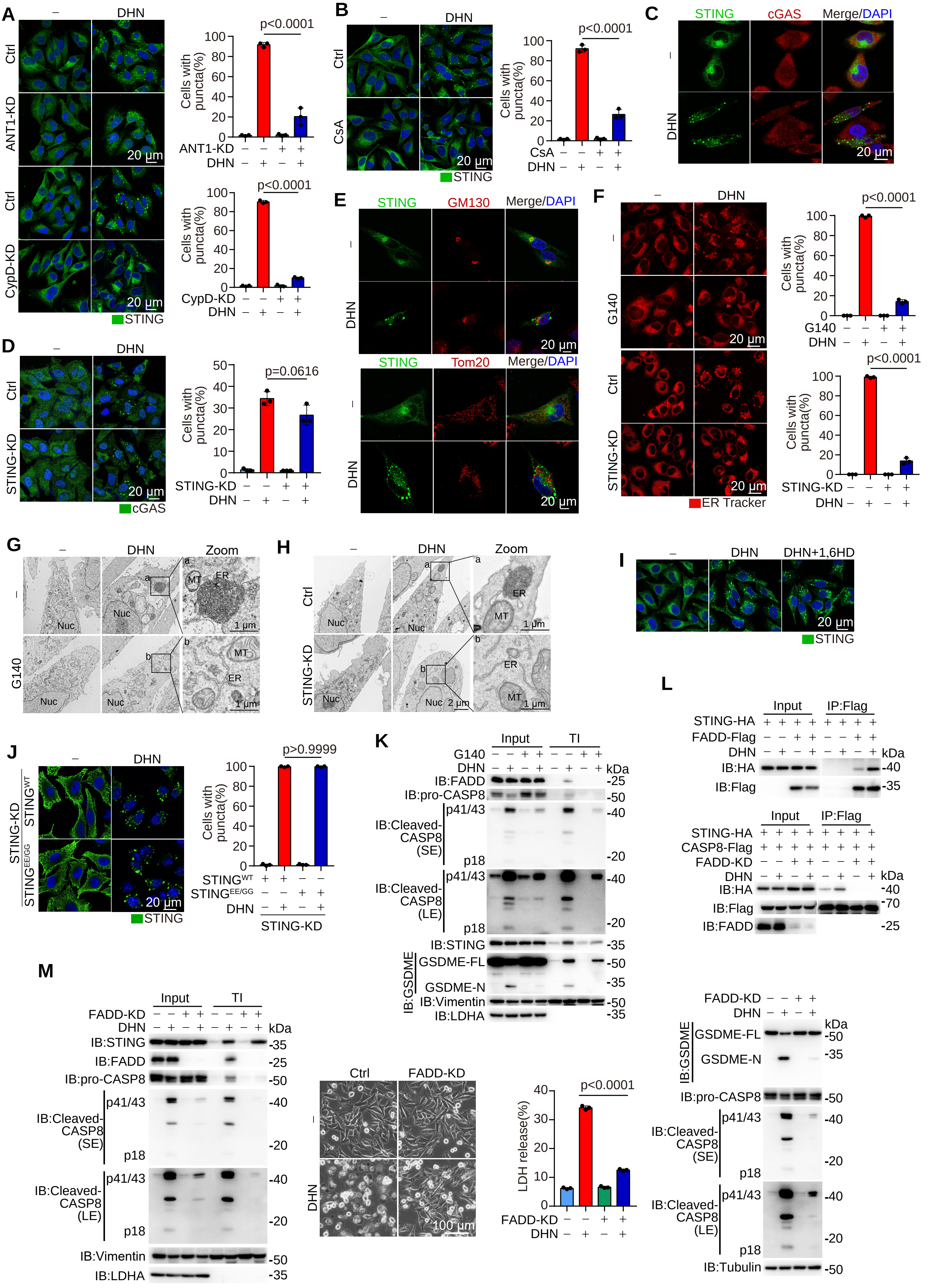

N

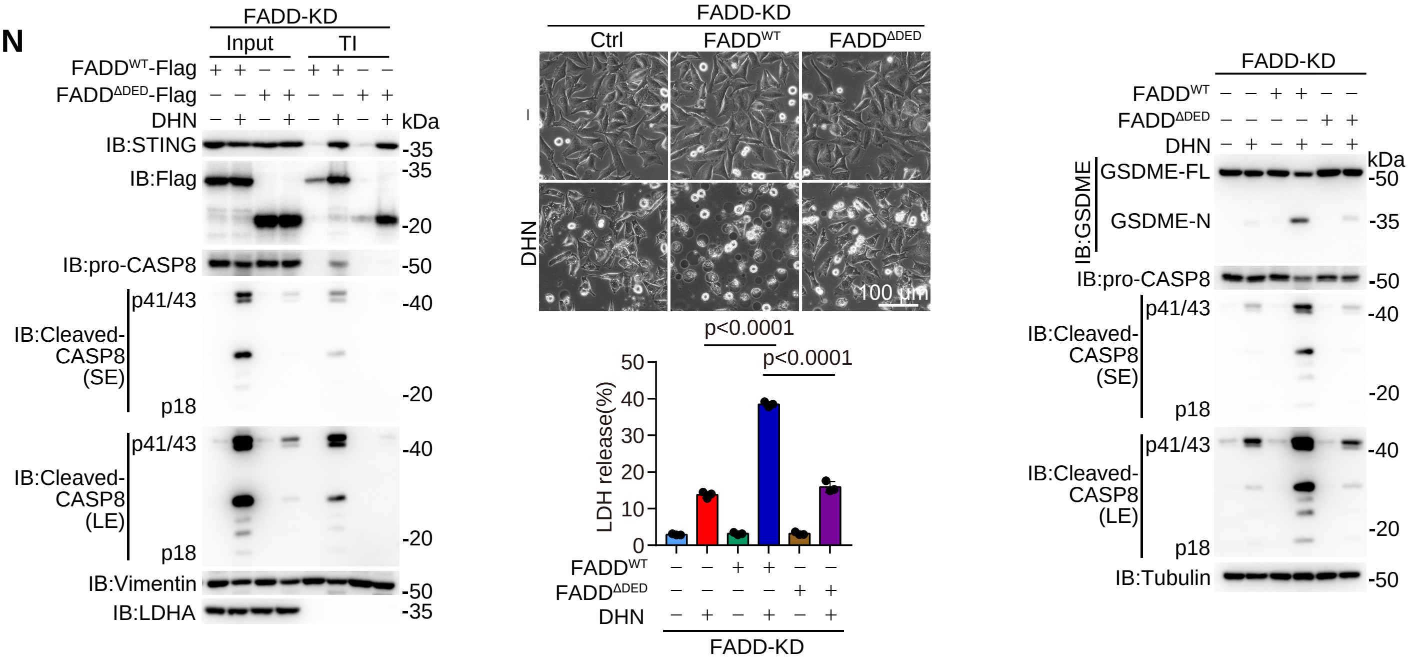

FADD-KD
Input
TI
FADDWT-Flag
FADDΔDED-Flag
DHN
kDa
IB:STING
IB:Flag
IB:pro-CASP8
IB:Cleaved-CASP8 (SE)
IB:Cleaved-CASP8 (LE)
p41/43
p18
IB:Vimentin
IB:LDHA
Ctrl
FADDWT
FADDΔDED
DHN
100 μm
p<0.0001
p<0.0001
LDH release(%)
FADD-KD
IB:GSDME
GSDME-FL
GSDME-N
IB:Tubulin

O

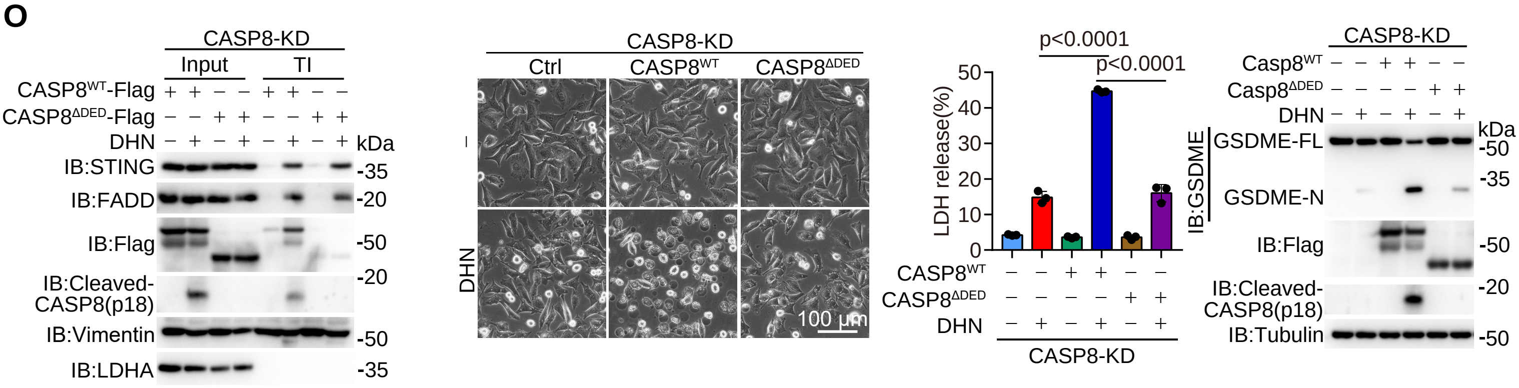

CASP8-KD
Input
TI
CASP8WT-Flag
CASP8ΔDED-Flag
DHN
kDa
IB:STING
IB:FADD
IB:Flag
IB:Cleaved-CASP8(p18)
IB:Vimentin
IB:LDHA
Ctrl
CASP8WT
CASP8ΔDED
DHN
100 μm
p<0.0001
p<0.0001
LDH release(%)
CASP8-KD
Casp8WT
Casp8ΔDED
IB:GSDME
GSDME-FL
GSDME-N
IB:Tubulin

Supplementary Figure 4

Fig. S4A-B Knocking down ANT1 or CypD, or CsA treatment suppresses the formation of STING puncta. The ANT1-knockdown (**A**, top) or CypD-knockdown (**A**, bottom) cells were treated with DHN (15 μM) for 12 hours, or cells were co-treated with CsA (**B**, 5 μM), and stained with STING antibody. STING puncta were observed under confocal microscope (left) and the percentage of cells with STING puncta was quantified (right).

Fig. S4C STING puncta show no spatial colocalization with cGAS puncta. STING-GFP was transfected into A375 cells and then treated with DHN (15 μM) for 12 hours, non-colocalization of STING puncta with cGAS was observed under confocal microscope.

Fig. S4D Knocking down STING cannot affect the formation of cGAS puncta. The STING -knockdown A375 cells were treated with DHN (15 μM) for 12 hours and stained with cGAS antibody. cGAS puncta were observed under confocal microscope (left) and the percentage of cells with cGAS puncta was quantified (right).

Fig. S4E STING puncta show no spatial colocalization with Golgi or mitochondria. STING-GFP was transfected into A375 cells and then treated with DHN (15 μM) for 12 hours, non-colocalization of STING puncta with GM130 (top, the Golgi marker) or Tom20 (bottom, the mitochondria marker) was observed under confocal microscope.

Fig. S4F G140 treatment or knocking down STING suppresses the formation of ER puncta. A375 cells were co-treated of G140 (30 μM) with DHN (15 μM) for 12 hours, or STING was knocked down in A375 cells first and then cells were treated with DHN

(15 μM) for 12 hours. Morphology of the ER stained with ER tracker was indicated (left) and the percentage of cells with ER puncta was quantified (right).

Fig. S4G-H G140 treatment or knocking down STING restores the ER structure to normal. A375 cells were co-treated of G140 (**g**, 30 μM) with DHN (15 μM) for 12 hours, or STING was knocked down (**H**) in A375 cells first and then cells were treated with DHN (15 μM) for 12 hours. Cells were collected as a sample for electron microscopy. The ER structure was observed under electron microscope.

Fig. S4I DHN-induced STING puncta is not a phase separation structure. A375 cells were treated with DHN (15 μM) for 12 hours and incubated with 1,6-HD (2.5%) for 5 mins. Anti-STING antibody was used to indicate the STING puncta observed under confocal microscope.

Fig. S4J The mutant $STING^{EE/GG}$ does not affect the formation of STING puncta. $STING^{WT}$ and $STING^{EE/GG}$ were separately transfected into STING-knockdown cells, and then cells were treated with DHN (15 μM) for 12 hours, followed by staining with STING antibody. STING puncta were observed under confocal microscope (left) and the percentage of cells with STING puncta was quantified (right).

Fig. S4K G140 treatment inhibits the enrichment of GSDME, cleaved-CASP8 and STING in TI components induced by DHN. The cells were co-treated of G140 (30 μM) and DHN (15 μM) for 12 hours. The localization of STING, cleaved-CASP8, and GSDME in the Triton X-100 insoluble fractions (TI) was indicated.

Fig. S4L DHN can enhance the interaction between FADD and STING. Knockdown of FADD can inhibit the interaction between STING and CASP8. In A375 cells

overexpressing STING-HA and FADD-Flag, treatment with DHN (15 μM) for 12 h demonstrated the binding between STING and FADD (top). In control and FADD-knockdown cells overexpressing STING-HA and CASP8-Flag, treatment with DHN (15 μM) for 12 h revealed the binding between STING and CASP8 (bottom).

Fig. S4M Knockdown of FADD suppresses pyroptosis. The FADD-knockdown cells were treated with DHN (15 μM) for 20 hours, and pyroptosis was assayed.

Fig. S4N The mutant $FADD^{\Delta DED}$ impairs DHN-induced pyroptosis. $FADD^{WT}$ and $FADD^{\Delta DED}$ were separately transfected into FADD-knockdown cells, and then cells were treated with DHN (15 μM) for 20 hours to detect pyroptosis.

Fig. S4O The mutant $CASP8^{\Delta DED}$ impairs DHN-induced pyroptosis. $CASP8^{WT}$ and $CASP8^{\Delta DED}$ were separately transfected into CASP8-knockdown cells, and then cells were treated with DHN (15 μM) for 20 hours to detect pyroptosis.

Statistical analyses were determined by two-way analysis of variance (ANOVA) with Tukey's multiple comparisons test (**S4A, S4B, S4D, S4F, S4J, S4M-S4O**). P values are indicated. All western blots were repeated at least twice and one of them is shown.

Supplementary Figure 5

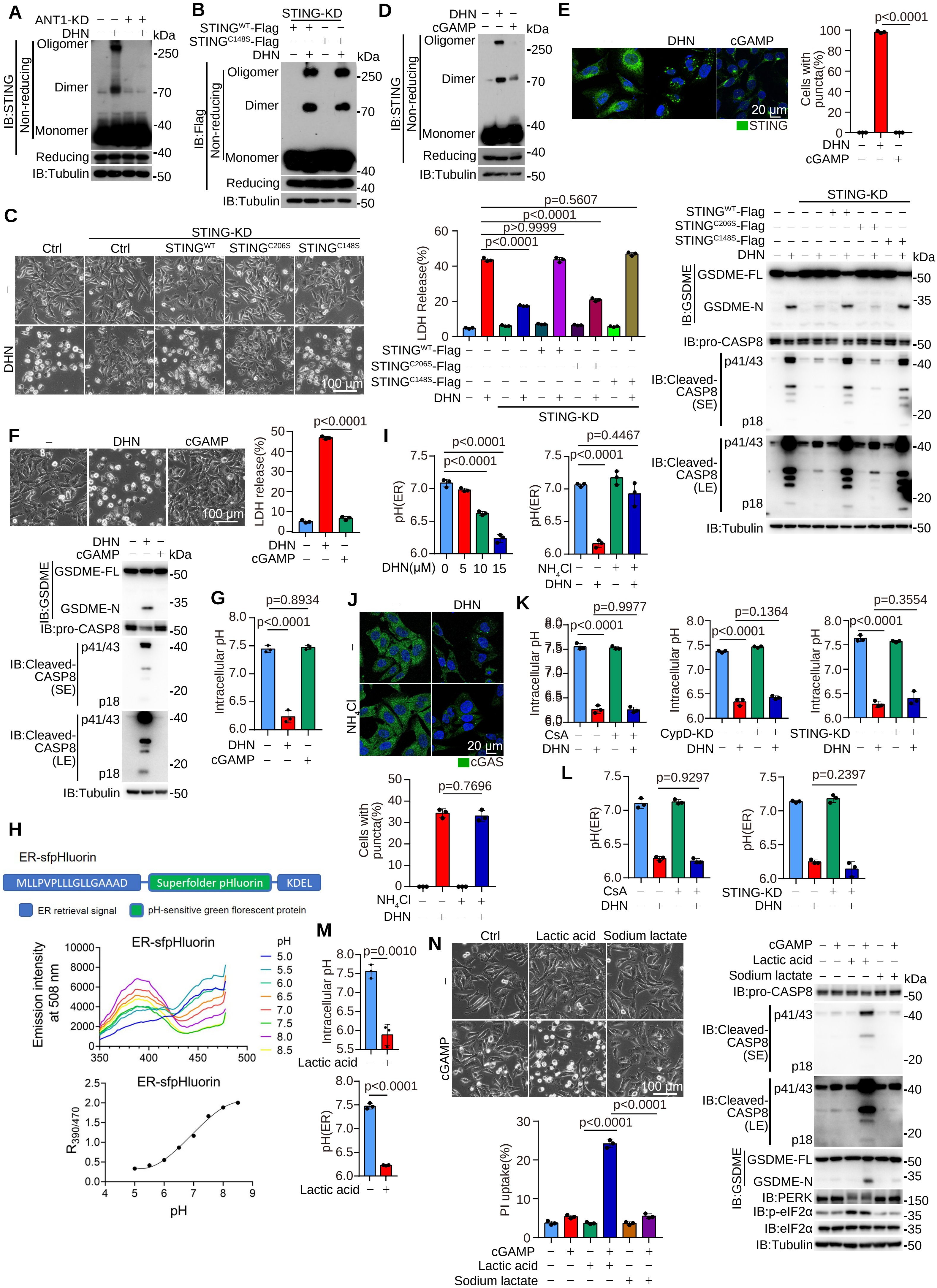

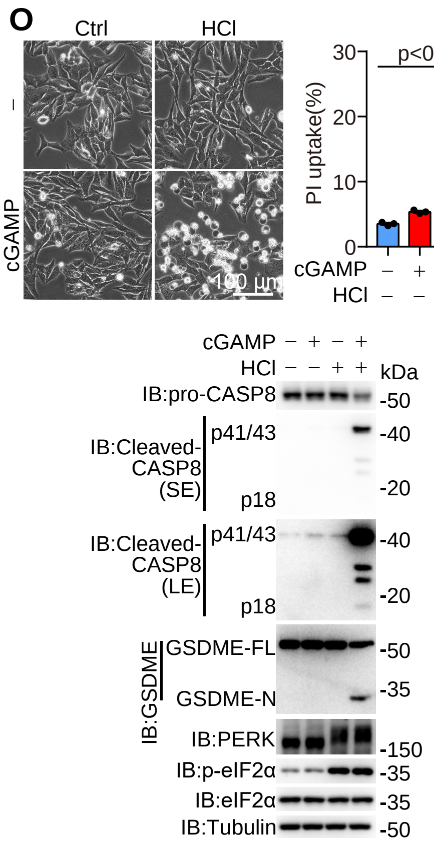
O
Ctrl
HCl
–
cGAMP
100 μm
PI uptake(%)
p<0.0001
cGAMP – + – +
HCl – – + +
kDa
IB:pro-CASP8
IB:Cleaved-CASP8 (SE)
p41/43
p18
IB:Cleaved-CASP8 (LE)
IB:GSDME
GSDME-FL
GSDME-N
IB:PERK
IB:p-eIF2α
IB:eIF2α
IB:Tubulin
-50
-40
-20
-35
-150

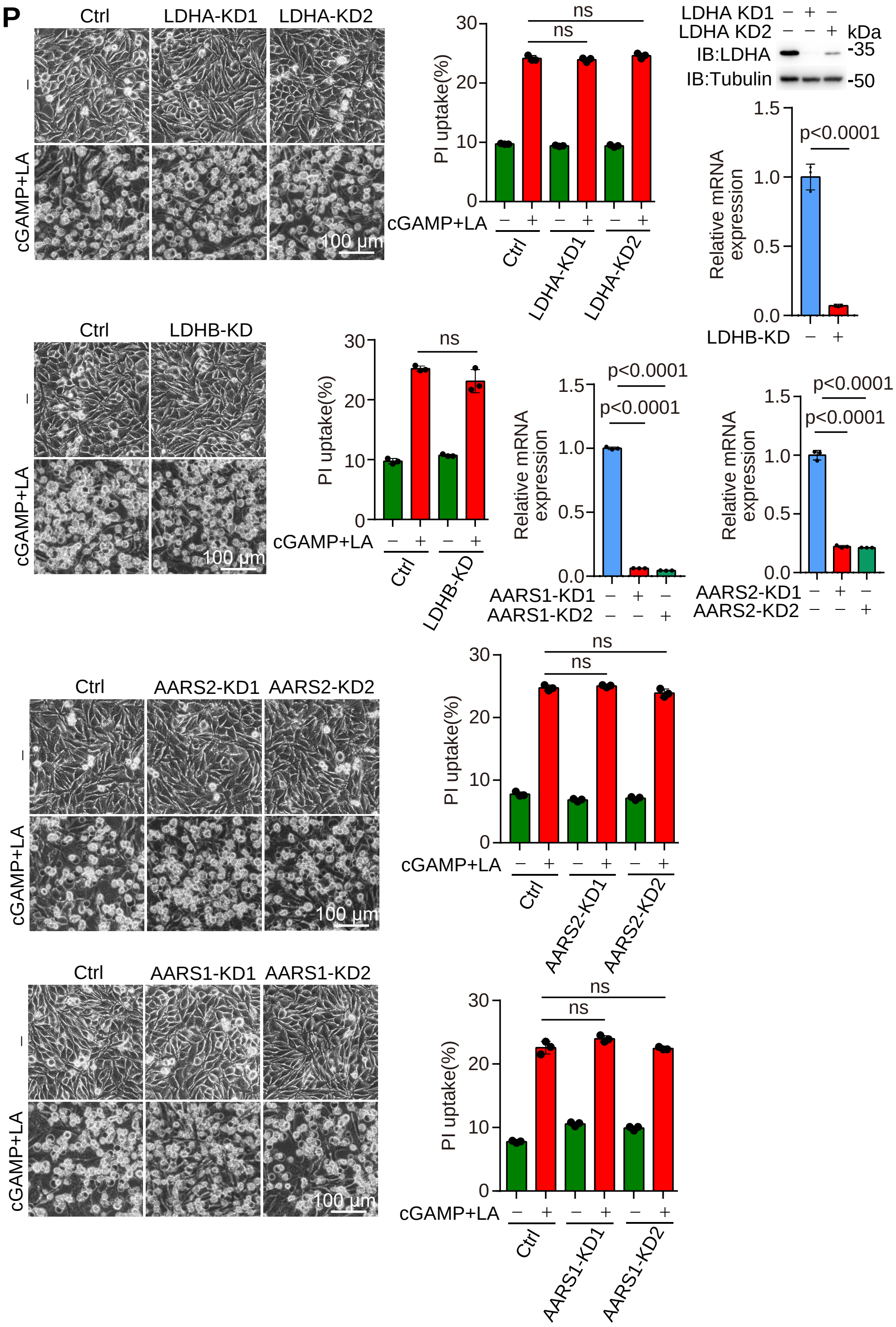
P
Ctrl
LDHA-KD1
LDHA-KD2
–
cGAMP+LA
100 μm
PI uptake(%)
ns
cGAMP+LA – + – + – +
LDHA KD1 – + –
LDHA KD2 – – +
kDa
IB:LDHA
IB:Tubulin
-35
-50
p<0.0001
Relative mRNA expression
LDHB-KD – +
Ctrl
LDHB-KD
AARS1-KD1 – + –
AARS1-KD2 – – +
AARS2-KD1 – + –
AARS2-KD2 – – +
Ctrl
AARS2-KD1
AARS2-KD2
Ctrl
AARS1-KD1
AARS1-KD2

Supplementary Figure 5

Fig. S5A Knocking down ANT1 suppresses the dimer and oligomers of STING. The ANT1-knockdown cells were treated with DHN (15 μM) for 12 hours, monomer, dimer and oligomers of STING were indicated.

Fig. S5B The mutant $STING^{C148S}$ does not affect the dimer and oligomers of STING. $STING^{WT}$ and $STING^{C148S}$ were separately transfected into STING-knockdown cells, and then cells were treated with DHN (15 μM) for 12 hours to detect STING monomer, dimer and oligomers.

Fig. S5C The mutant $STING^{C206S}$, but not $STING^{C148S}$, impairs DHN-induced pyroptosis. $STING^{WT}$, $STING^{C206S}$ and $STING^{C148S}$ were separately transfected into STING-knockdown cells, and then cells were treated with DHN (15 μM) for 12 hours to detect pyroptosis.

Fig. S5D-G DHN, but not cGAMP, possesses the capability to induce pyroptosis, promote STING aggregation, and attenuate cellular pH levels. Cells were treated with DHN (15 μM) or cGAMP (10 μg/ml) to detect dimer and oligomers of STING (**S5D**), STING puncta (**S5E**), pyroptosis (**S5F**) and cytosolic pH values (**S5G**).

Fig. S5H The structure of ER-sfpHluorin fluorescent protein (top), pH-dependent excitation spectra of ER-sfpHluorin (Middle) and the calibration curves of fluorescence with different pH value (bottom) are indicated. ER-sfpHluorin was transfected into A375 cells and the cells were permeabilized with digitonin and then resuspended in citric acid/Na_2HPO_4 buffer of pH values ranging from 5.0 to 8.5. The emission intensity was recorded at 508nm under Tecan Infinite E plex plate reader.

Fig. S5I Detection of ER pH value. Cells were treated wtih DHN at different concentrations (left) or co-treated of NH_4Cl (5 mM) with DHN (15 μM) for 12 h (right), the ER pH value was measured using the ER-located and pH-sensitive fluorescent protein.

Fig. S5J NH_4Cl does not affect the formation of cGAS puncta. Cells were co-treated of NH_4Cl (5 mM) with DHN (15 μM) for 12 hours, and stained with cGAS antibody. cGAS puncta were observed under confocal microscope (top) and the percentage of cells with cGAS puncta was quantified (bottom).

Fig. S5K CsA treatment**,** knocking down CypD or STING does not impair the decrease of cytoplasmic pH. Cells were co-treated of CsA (10 μM) with DHN (15 μM) for 12 hours, or the Cypd-knockdown or STING- knockdown cells were were treated with DHN (15 μM) for 12 hours, followed by measurement of cytosolic pH values.

Fig. S5L CsA treatment or knocking down STING does not impair the decrease of ER pH. Cells were co-treated of CsA (5 μM) with DHN, or the STING-knockdown cells were treated with DHN, followed by measurement of ER pH values.

Fig. S5M Lactic acid decreases cytosolic and ER pH. Cells were treated with lactic acid (20 mM), followed by measurement of cytosolic and ER pH values.

Fig. S5N Lactic acid, in combination with cGAMP, triggers pyroptosis, whereas sodium lactate cannot. Cells were co-treated of 2,3'-GAMP (10 μg/ml) and lactic acid (20 mM) or sodium lactate (20 mM) for 36 h, followed by the detection of pyroptosis.

Fig. S5O HCl, in combination with cGAMP, triggers pyroptosis. Cells were co-treated of 2,3'-GAMP (10 μg/ml) and HCl (20 mM) for 36 h, followed by the detection of pyroptosis.

Fig. S5P Knockdown of LDHA, LDHB, AARS1, or AARS2 fails to suppress lactic acid- and cGAMP-induced pyroptosis. In A375 cells, knockdown of LDHA, LDHB, AARS1, and AARAS was performed individually. Pyroptosis was assessed after 36 h of co-treatment with lactic acid (20 mM) and 2,3'-GAMP (10 μg/ml).

Tubulin was used to determine the amount of loading proteins. Statistical analyses were determined by two-way analysis of variance (ANOVA) with Tukey's multiple comparisons test (**S5C, S5I-S5L, S5N-S5P**), one-way ANOVA with Tukey's multiple comparisons test (**S5E-S5G, S5I**) and unpaired two-tailed Student's t-test (**S5M**). P values are indicated. All western blots were repeated at least twice and one of them is shown.

Supplementary Figure 6

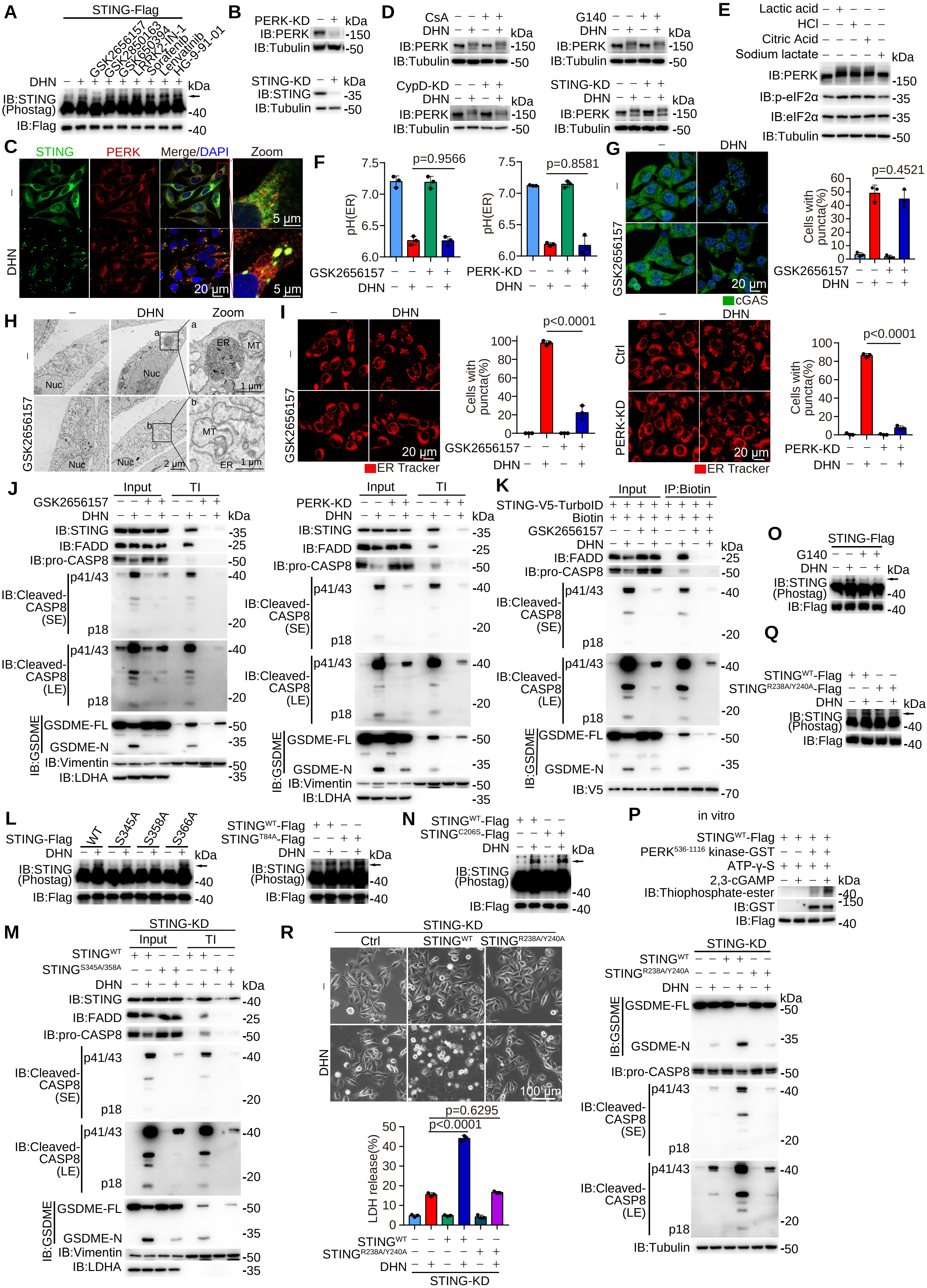

Supplementary Figure 6

Fig. S6A GSK2656157 can inhibit the STING phosphorylation induced by DHN. Cells were co-treated of GSK2656157 (10 μM), GSK2850163 (10 μM), GSK650394 (10 μM), LRRK2-IN-1 (0.2 μM), Sorafenib (0.5 μM), Lenvatinib (0.5 μM) or HG-9-91-01 (0.5 μM) with DHN (15 μM) for 12 hours as indicated, the STING phosphorylation was indicated.

Fig. S6B Efficiencies of knocking down PERK or STING in A375 cells, detected by western blotting.

Fig. S6C PERK shows spatially colocalized with STING puncta. PERK-HA was transfected into A375 cells and then the cells were treated with DHN (15 μM) for 12 hours, PERK and STING localization was observed under confocal microscope.

Fig. S6D The phosphorylation of PERK remains unaffected by treatments of CsA or G140, or knockdown of CypD or STING. A375 cells were co-treated of CsA (5 μM) or G140 (30 μM) with DHN (15 μM) for 12 hours, or CypD or STING was knocked down in A375 cells and the cells were treated with DHN (15 μM) for 12 hours. An up-shift band of PERK (indication of PERK phosphorylation) was indicated.

Fig. S6E Cellular acidification induces PERK phosphorylation. A375 cells were treated wtih lactic acid (20 mM), HCl (20mM), Citric acid (2mM), or sodium lactate (20mM) for 12 hours. An up-shift band of PERK (indication of PERK phosphorylation) was indicated.

Fig. S6F GSK2656157 treatment or knocking down PERK cannot affect the pH value of ER. A375 cells were co-treated of GSK2656157 (10 μM) with DHN (15 μM) for 12

hours or PERK was knocked down in A375 cells and the cells were treated wtih DHN (15 μM) for 12 hours. The pH value of ER was measured.

Fig. S6G GSK2656157 treatment cannot affect the formation of cGAS puncta. Cells were co-treated of GSK2656157 (10 μM) with DHN (15 μM) for 12 hours and stained with cGAS antibody. cGAS puncta were observed under confocal microscope (left) and the percentage of cells with cGAS puncta was quantified (right).

Fig. S6H GSK2656157 restores the ER structure to normal. A375 cells were co-treated of GSK2656157 (10 μM) with DHN (15 μM) for 12 hours, and the ER structure was observed under electron microscope.

Fig. S6I GSK2656157 treatment or knocking down PERK inhibits the formation of ER puncta. Cells were co-treated of GSK2656157 (10 μM) with DHN (15 μM) for 12 hours, or subjected to PERK knockdown, followed by the detection of ER puncta using confocal microscope.

Fig. S6J GSK2656157 treatment or knocking down PERK inhibits the enrichment of GSDME, cleaved-CASP8 and STING in TI components induced by DHN. Cells were co-treated of GSK2656157 (left, 10 μM) with DHN (15 μM) for 12 hours, or subjected to PERK knockdown (right). The localization of STING, cleaved-CASP8, and GSDME in the Triton X-100 insoluble fractions (TI) was indicated.

Fig. S6K GSK2656157 treatment impairs DHN-induced STING polymerization cleavage of GSDME by caspase-8 within ER-STING dependent pyroptosome. Cells were transfected with GFP-V5-turboID or STING-V5-turboID and co-treated of GSK2656157 (10 μM) with DHN (15 μM) for 12 hours, then labeled with biotin (100

μM) for 10 mins, the biotin-labeled proteins were isolated and indicated by corresponding antibodies.

Fig. S6L The mutants $STING^{S345A}$ and $STING^{S358A}$ impair DHN-induced STING phosphorylation. $STING^{WT}$, $STING^{S345A}$, $STING^{S358A}$, $STING^{S366A}$ and $STING^{T84A}$ were overexpressed in cells, treated with DHN, and STING phosphorylation was subsequently detected.

Fig. S6M The mutant $STING^{S345/S358A}$ suppresses DHN-induced enrichment of STING, cleaved-CASP8 and GSDME in TI. $STING^{WT}$ and $STING^{S345A/S358A}$ were separately transfected into STING-knockdown cells, and then cells were treated with DHN (15 μM) for 12 hours to detect the localization of STING, cleaved-CASP8, and GSDME in the TI.

Fig. S6N The mutant $STING^{S206S}$ cannot impair DHN-induced STING phosphorylation. $STING^{WT}$ or $STING^{S206S}$ was overexpressed in cells, treated with DHN (15 μM) for 12 hours, and STING phosphorylation was subsequently detected.

Fig. S6O G140 treatment inhibits STING phosphorylation. A375 cells were co-treated with G140 (30 μM) and DHN (15 μM) for 12hours. STING phosphorylation was subsequently detected.

Fig. S6P cGAMP treatment enhances the phosphorylation of STING by PERK *in vitro*. The protein of $STING^{WT}$ was incubated with PERK kinase protein in the presence of absence of cGAMP. The phosphorylation of STING was showed.

Fig. S6Q The mutant STING$^{R238A/Y240A}$ impairs DHN-induced STING phosphorylation. STING^{WT} and STING$^{R238A/Y240A}$ were overexpressed in cells, treated with DHN (15 μM) for 12 hours, and STING phosphorylation was subsequently detected.

Fig. S6R The mutant STING$^{R238A/Y240A}$ impairs DHN-induced pyroptosis. STING^{WT} and STING$^{S345/S358A}$ were separately transfected into STING-knockdown cells, and then cells were treated with DHN (15 μM) for 20 hours to detect pyroptosis.

Tubulin was used to determine the amount of loading proteins. Statistical analyses were determined by two-way analysis of variance (ANOVA) with Tukey's multiple comparisons test (**S6F, S6G, S6I, S6R**). P values are indicated. All western blots were repeated at least twice and one of them is shown.

Supplementary Figure 7

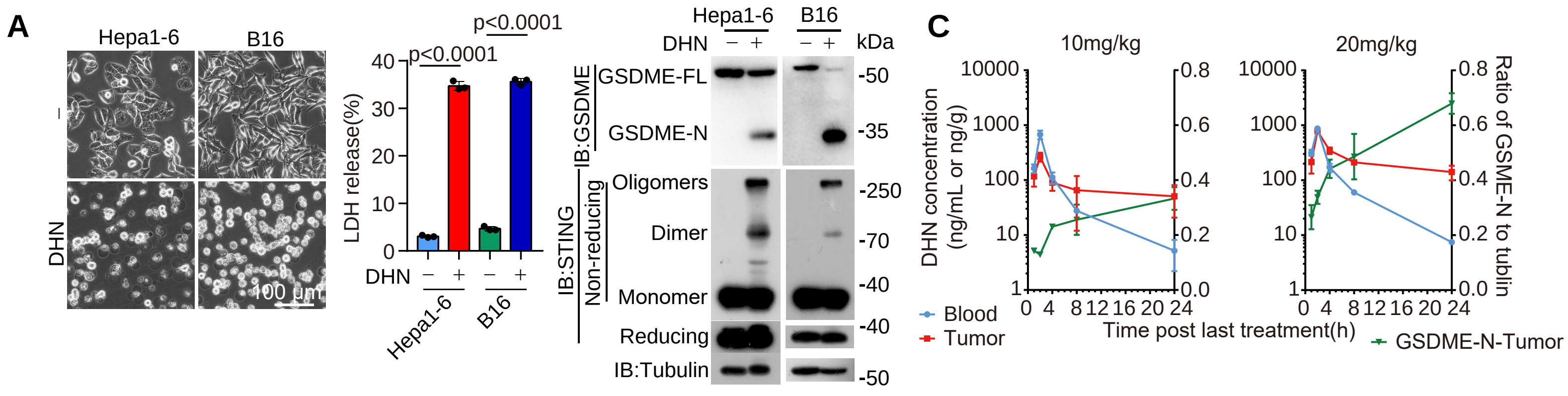

Administration	T1/2 (hr)	Tmax (hr)	Cmax (ng/mL)	AUC $_{0-t}$ (ng·hr/mL)	AUC $_{0-\infty}$ (ng·hr/mL)
I.p. (20 mg/kg)	4.66	2.00	875	2968	3018

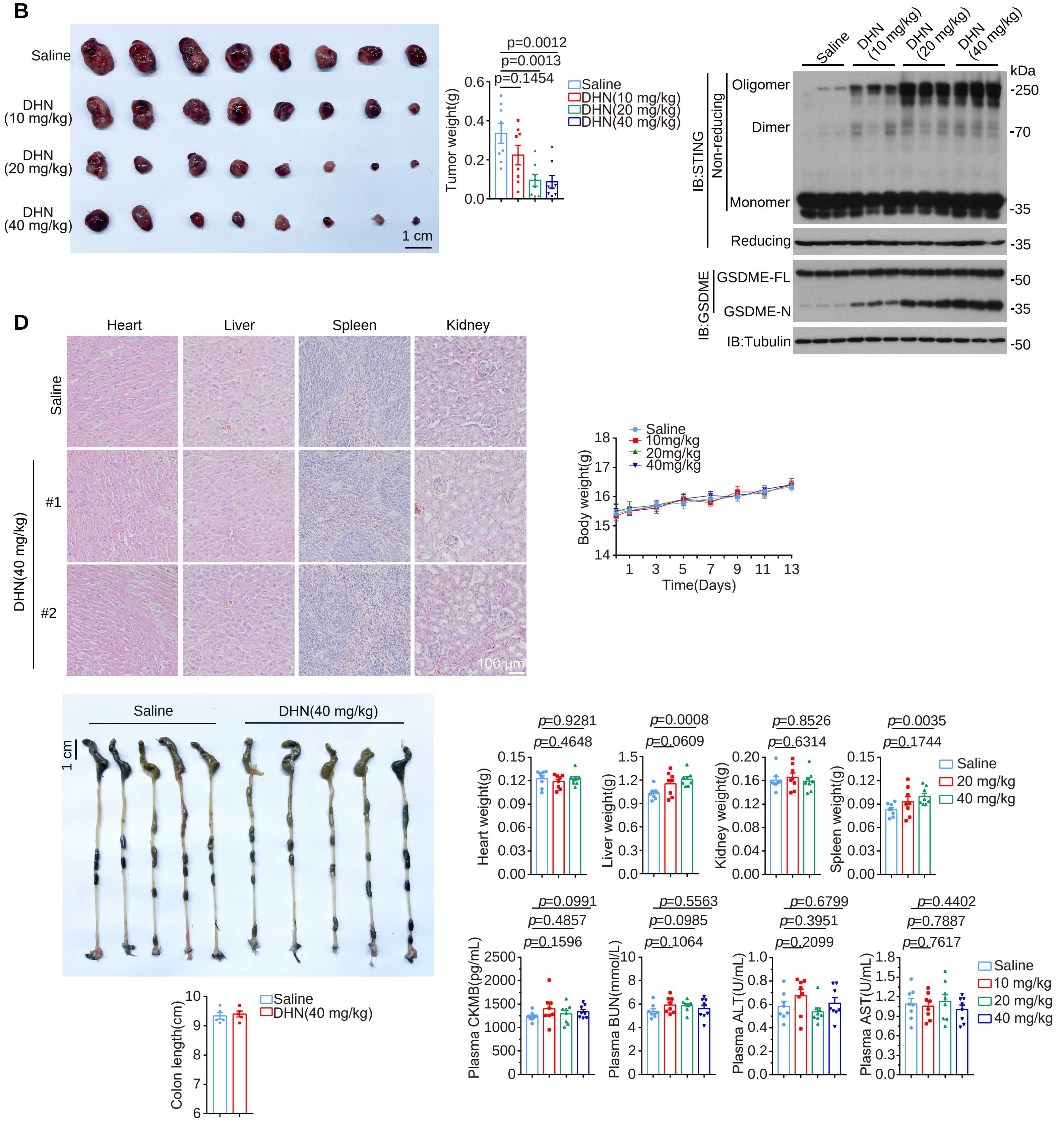

Supplementary Figure 7

Fig. S7A DHN can induce pyroptosis in B16 and Hepa1-6 cells. B16 or Hepa1-6 cells were treated with DHN (15 μM) for 20 hours. Pyroptosis was detected with morphology, GSDME cleavage, LDH release and oligomers of STING.

Fig. S7B DHN can inhibit tumor growth in a dose-dependent manner. B16 cells (1×10^5) were subcutaneously injected into mice for four days and then DHN (20 mg/kg) was administered to the mice every day for one week. Tumors were weighed and collected for detection of GSDME, monomer, dimer and oligomers of STING.

Fig. S7C Kinetics of cleavage of GSDME in B16 subcutaneous tumors after post administration of DHN at indicated doses ($n = 3$ mice for each treatment point). Total blood and tumor exposures of DHN were measured. Tumors were collected for detection of GSDME-N.

Fig. S7D DHN exhibits minimal toxicity in mice. B16 cells (1×10^5) were subcutaneously injected into mice for four days and then DHN (20 mg/kg) was administered. The weight of body, liver, spleen, heart and kidney were recorded (n = 8). HE and toxicity kit were used to detect the side effects of heart, liver, spleen and kidney (n = 8). The length of the colon of the mice was measured (n = 5).

Tubulin was used to determine the amount of loading proteins. Statistical analyses were determined by unpaired two-tailed Student's t-test. P values are indicated.
