# Supplementary material for: cGAS activation converges with intracellular acidification to promote STING aggregation and pyroptosis in tumor models
Source: J Clin Invest. 2025 Jul 15;135(18):e188872. doi: 10.1172/JCI188872 (PMC12435844; doi:10.1172/JCI188872)

Figure 1

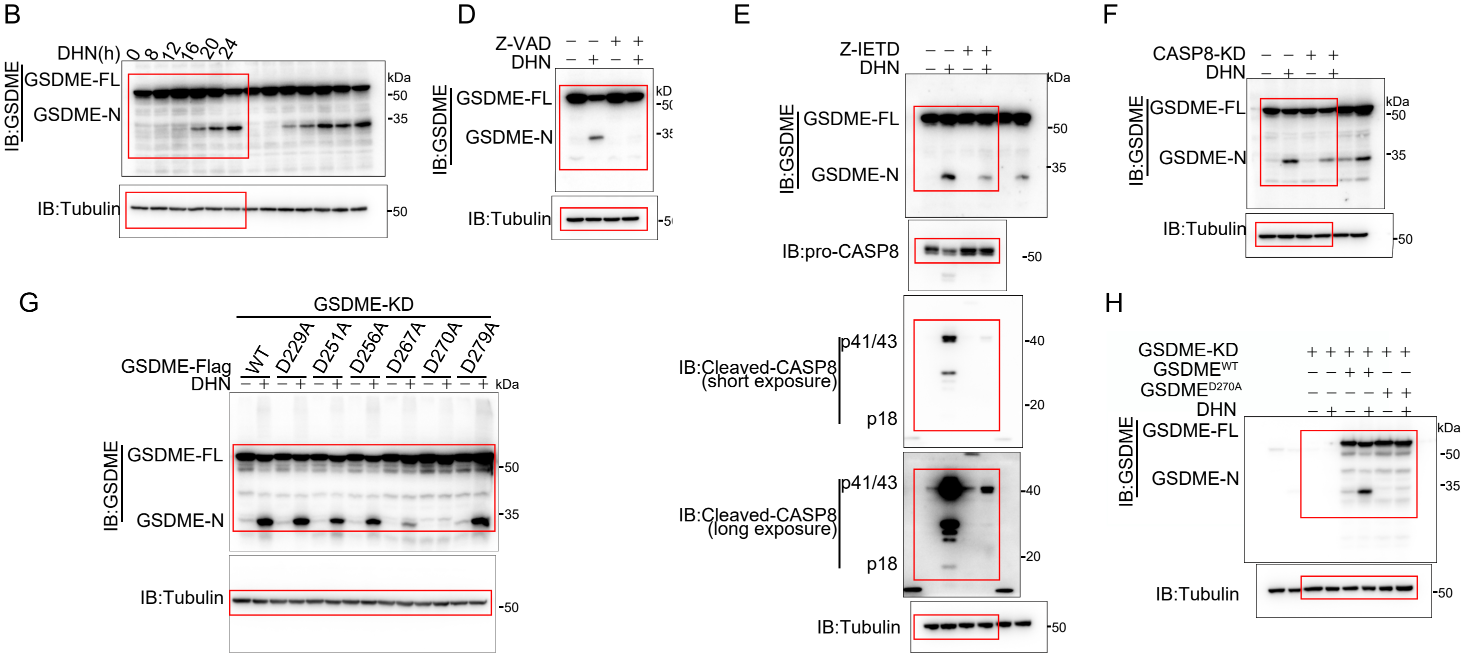

Figure 2

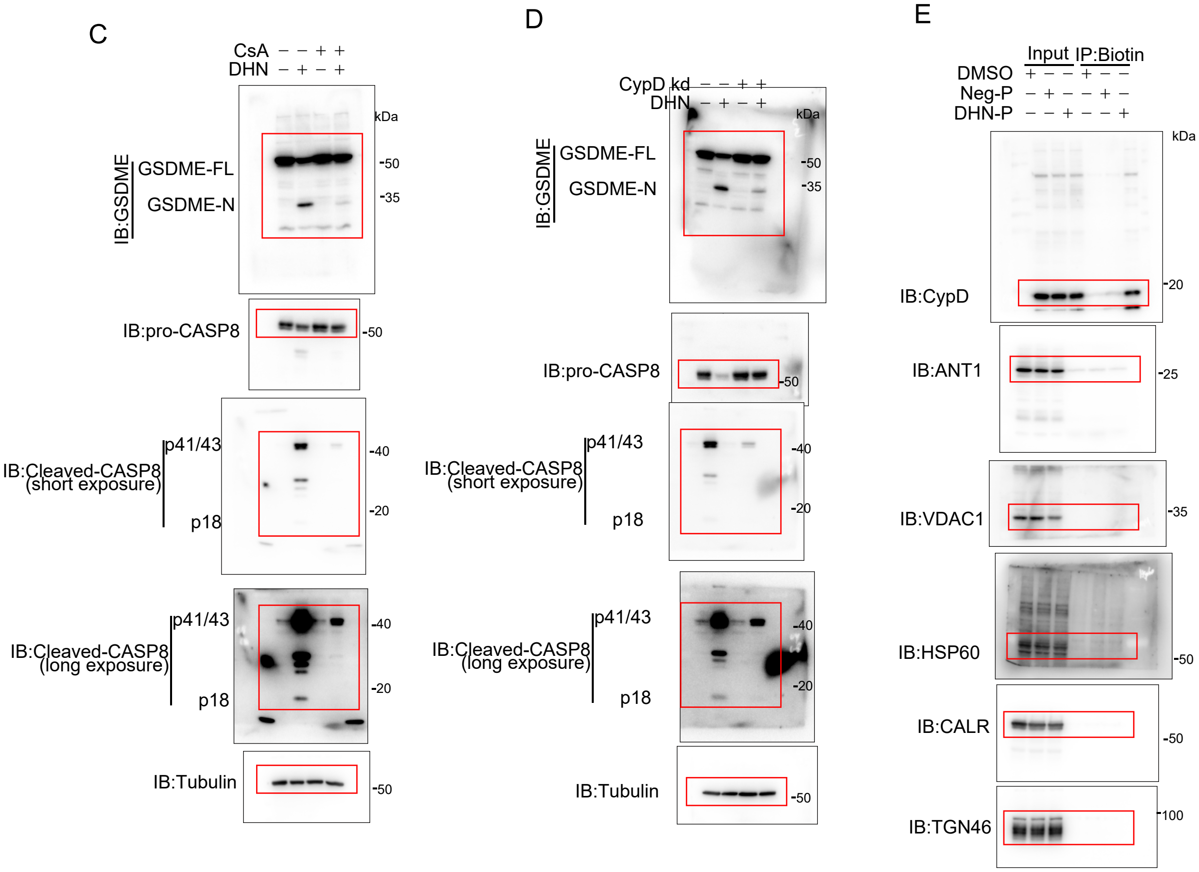

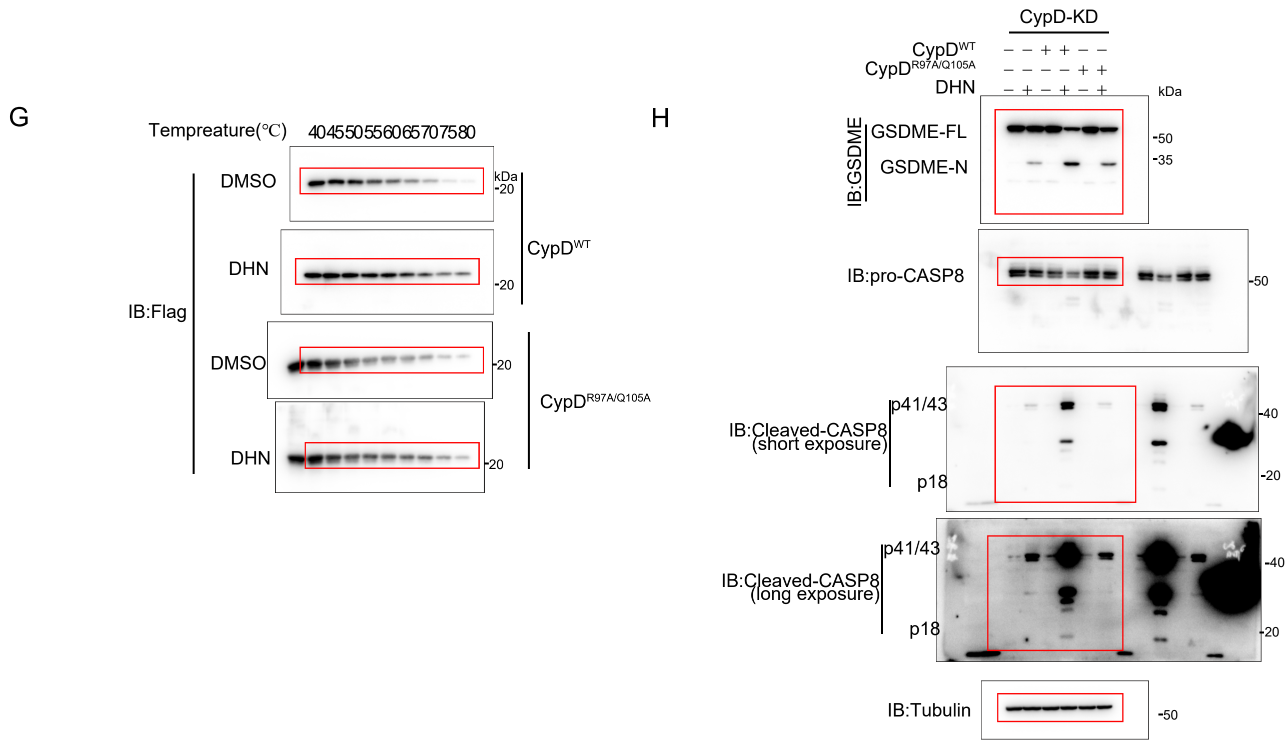
G
Tempreature(℃) 40 45 50 55 60 65 70 75 80
IB:Flag
DMSO
DHN
DMSO
DHN
CypD^WT
CypD^R97A/Q105A
kDa
-20
H
CypD-KD
CypD^WT − − + + − −
CypD^R97A/Q105A − − − − + +
DHN − + − + − +
kDa
IB:GSDME
GSDME-FL
GSDME-N
-50
-35
IB:pro-CASP8
IB:Cleaved-CASP8 (short exposure)
IB:Cleaved-CASP8 (long exposure)
p41/43
p18
-40
-20
IB:Tubulin
-50

Figure 3

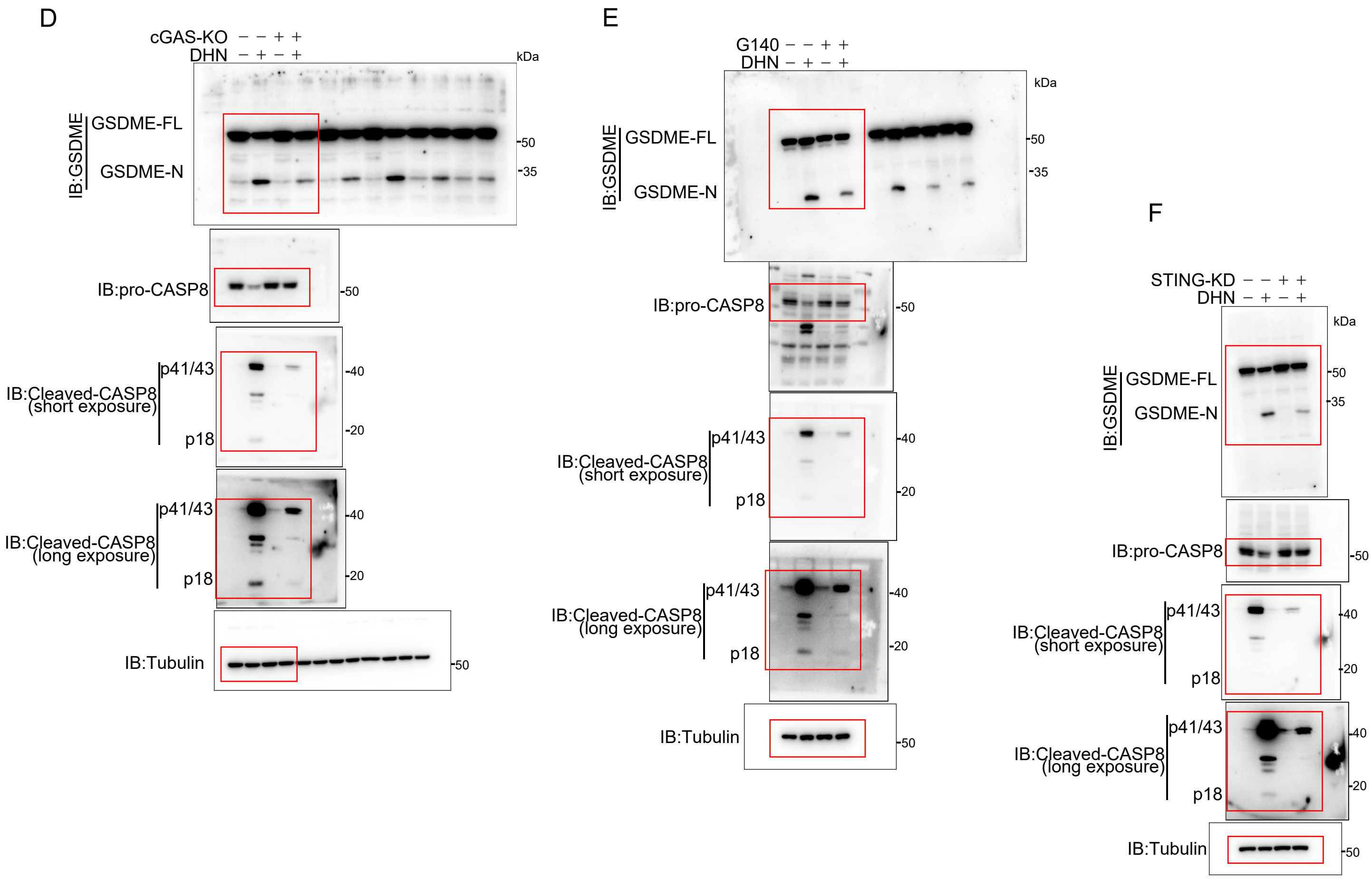
D
cGAS-KO − − + +
DHN − + − +
kDa
IB:GSDME
GSDME-FL
GSDME-N
-50
-35
IB:pro-CASP8
IB:Cleaved-CASP8 (short exposure)
IB:Cleaved-CASP8 (long exposure)
p41/43
p18
-40
-20
IB:Tubulin
E
G140 − − + +
DHN − + − +
F
STING-KD − − + +
DHN − + − +

Figure 4

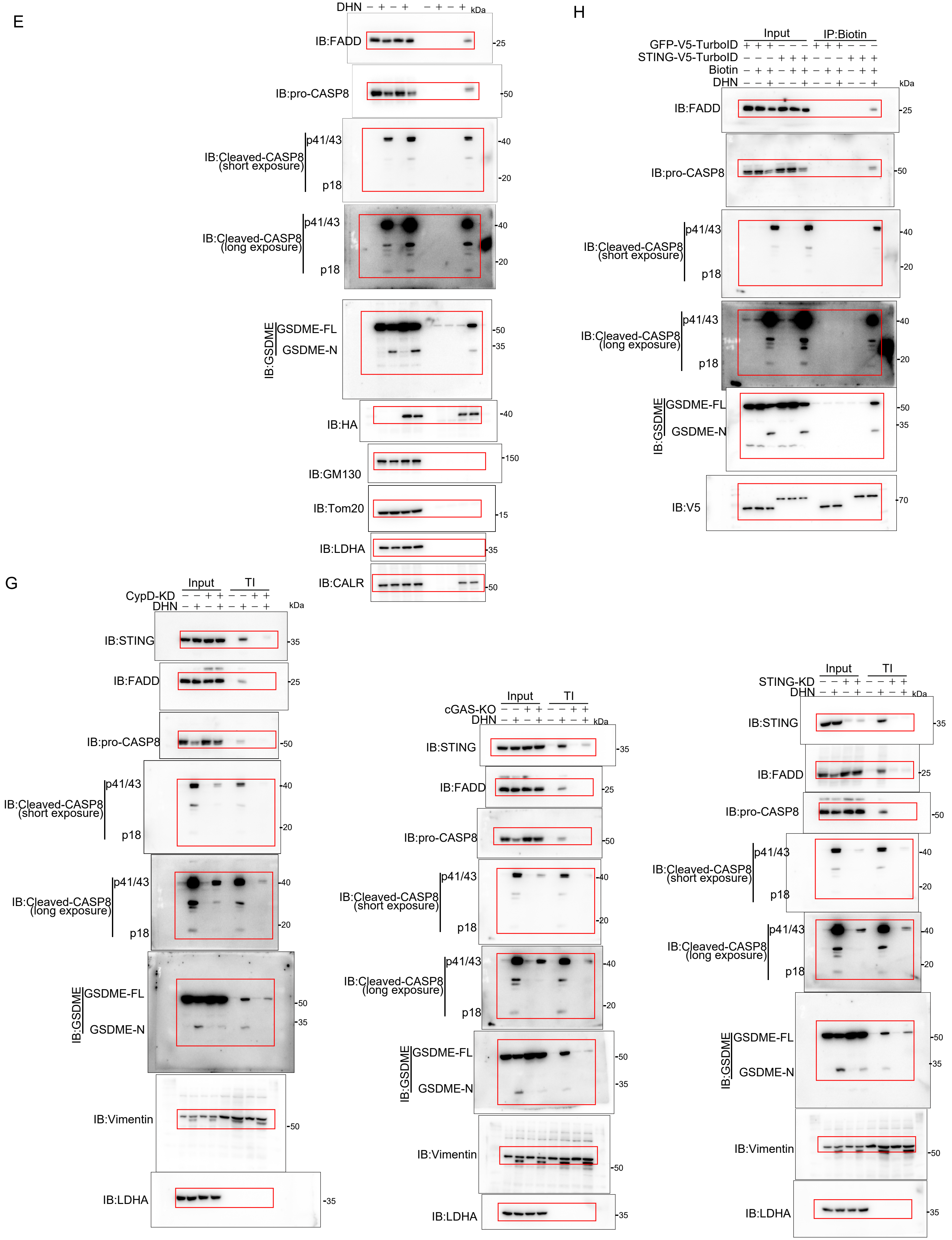
E
Input
IP:HA(ER)
STING-HA
DHN
kDa
IB:FADD
IB:pro-CASP8
IB:Cleaved-CASP8 (short exposure)
IB:Cleaved-CASP8 (long exposure)
p41/43
p18
IB:GSDME
GSDME-FL
GSDME-N
IB:HA
IB:GM130
IB:Tom20
IB:LDHA
IB:CALR
H
Input
IP:Biotin
GFP-V5-TurboID
STING-V5-TurboID
Biotin
DHN
IB:V5
G
Input
TI
CypD-KD
IB:STING
IB:Vimentin
cGAS-KO
STING-KD

Figure 5

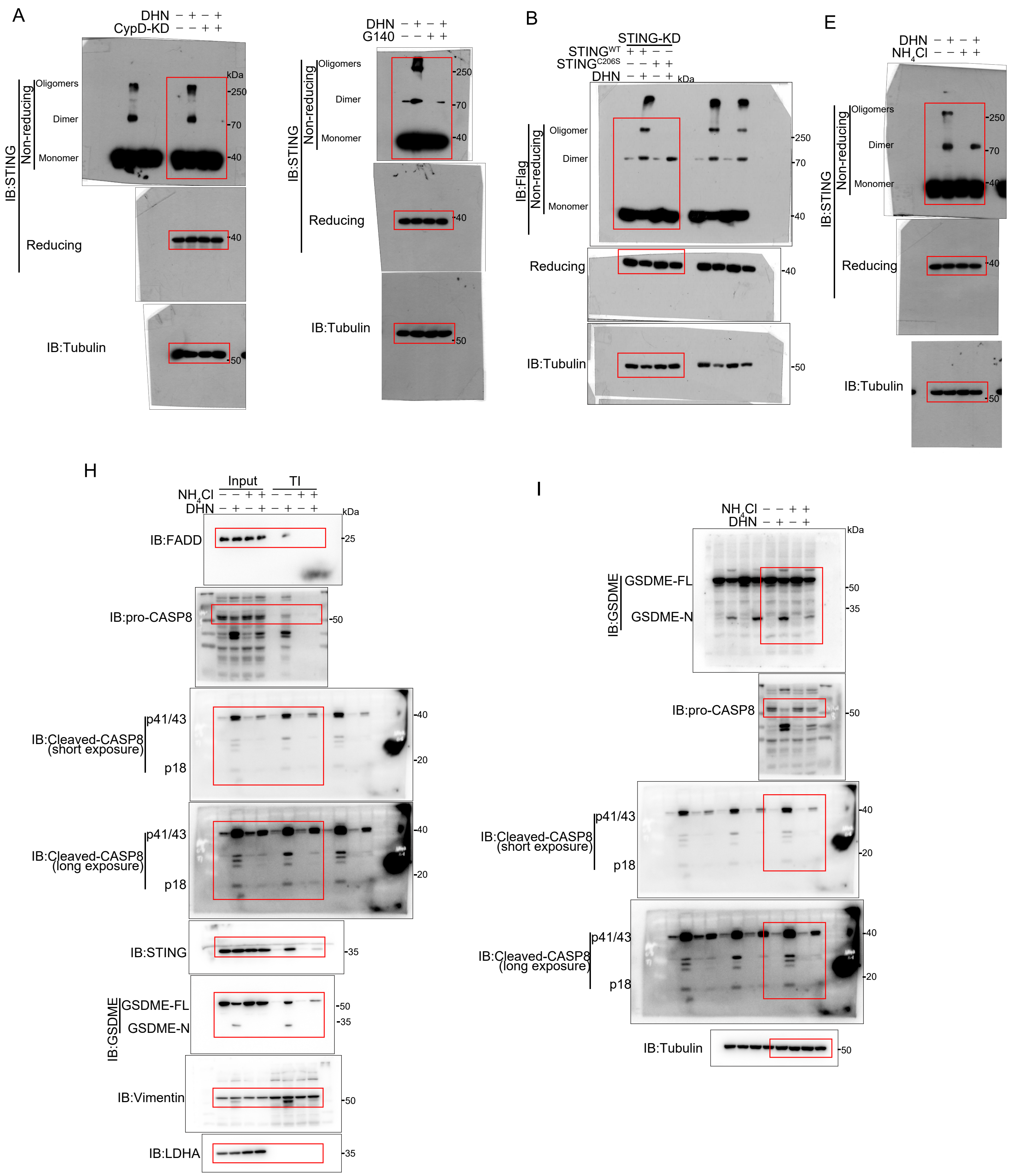
A
DHN − + − +
CypD-KD − − + +
IB:STING
Non-reducing
Oligomers
Dimer
Monomer
Reducing
IB:Tubulin
kDa
250
70
40
40
50
DHN − + − +
G140 − − + +
Oligomers
Dimer
Monomer
250
70
40
IB:STING
Non-reducing
Reducing
40
IB:Tubulin
50
B
STING-KD
STING^WT + + − −
STING^C206S − − + +
DHN − + − +
kDa
IB:Flag
Non-reducing
Oligomer
Dimer
Monomer
250
70
40
Reducing
40
IB:Tubulin
50
E
DHN − + − +
NH4Cl − − + +
IB:STING
Non-reducing
Oligomers
Dimer
Monomer
250
70
40
Reducing
40
IB:Tubulin
50
H
Input
TI
NH4Cl − − + + − − + +
DHN − + − + − + − +
kDa
IB:FADD
25
IB:pro-CASP8
50
IB:Cleaved-CASP8 (short exposure)
p41/43
p18
40
20
IB:Cleaved-CASP8 (long exposure)
p41/43
p18
40
20
IB:STING
35
IB:GSDME
GSDME-FL
GSDME-N
50
35
IB:Vimentin
50
IB:LDHA
35
I
NH4Cl − − + +
DHN − + − +
kDa
IB:GSDME
GSDME-FL
GSDME-N
50
35
IB:pro-CASP8
50
IB:Cleaved-CASP8 (short exposure)
p41/43
p18
40
20
IB:Cleaved-CASP8 (long exposure)
p41/43
p18
40
20
IB:Tubulin
50

Figure 6

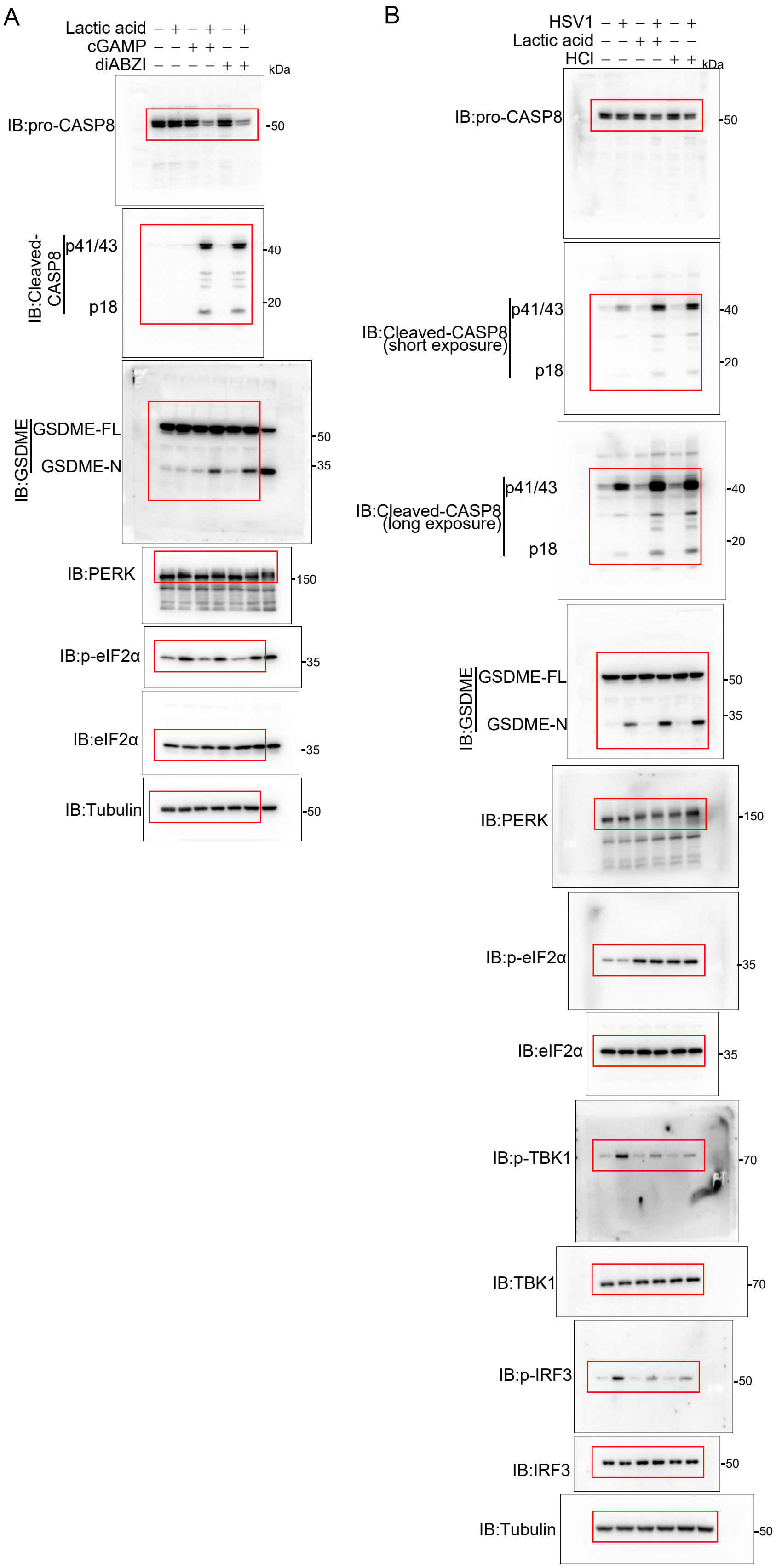
A
Lactic acid – + – + – +
cGAMP – – + + – –
diABZI – – – – + +
kDa
IB:pro-CASP8
50
IB:Cleaved-CASP8
p41/43
p18
40
20
IB:GSDME
GSDME-FL
GSDME-N
50
35
IB:PERK
150
IB:p-eIF2α
35
IB:eIF2α
35
IB:Tubulin
50
B
HSV1 – + – + – +
Lactic acid – – + + – –
HCl – – – – + +
kDa
IB:pro-CASP8
50
IB:Cleaved-CASP8 (short exposure)
p41/43
p18
40
20
IB:Cleaved-CASP8 (long exposure)
p41/43
p18
40
20
IB:GSDME
GSDME-FL
GSDME-N
50
35
IB:PERK
150
IB:p-eIF2α
35
IB:eIF2α
35
IB:p-TBK1
70
IB:TBK1
70
IB:p-IRF3
50
IB:IRF3
50
IB:Tubulin
50

Figure 7

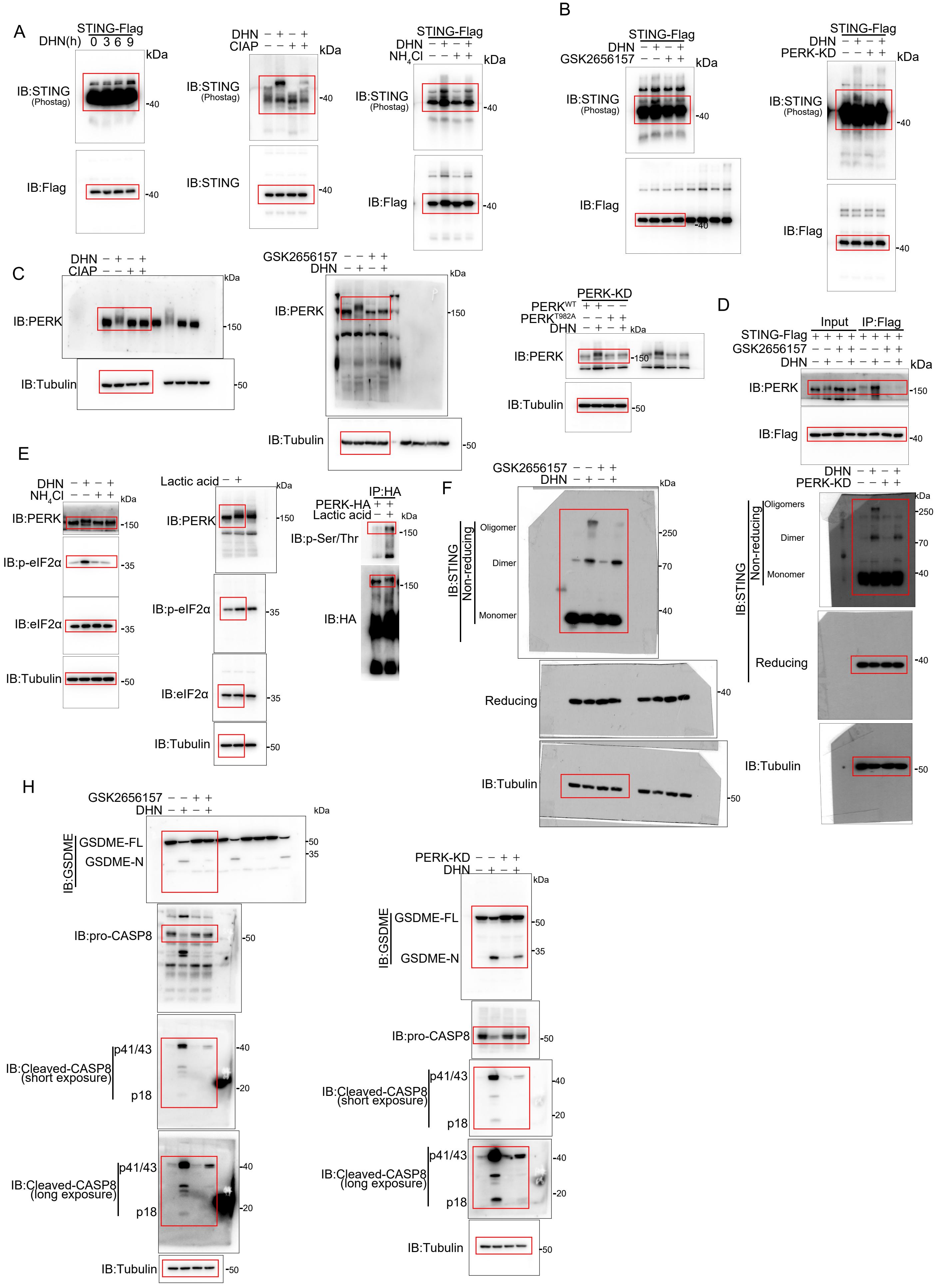
A
STING-Flag
DHN(h) 0 3 6 9
kDa
IB:STING (Phostag)
IB:Flag
DHN − + − +
CIAP − − + +
IB:STING
NH4Cl
B
GSK2656157
PERK-KD
C
IB:PERK
IB:Tubulin
PERK-KD
PERKWT
PERKT982A
D
Input
IP:Flag
E
IB:p-eIF2α
IB:eIF2α
Lactic acid
IP:HA
PERK-HA
IB:p-Ser/Thr
IB:HA
F
IB:STING Non-reducing
Oligomer
Dimer
Monomer
Reducing
Oligomers
H
IB:GSDME
GSDME-FL
GSDME-N
IB:pro-CASP8
IB:Cleaved-CASP8 (short exposure)
IB:Cleaved-CASP8 (long exposure)
p41/43
p18

Figure 8

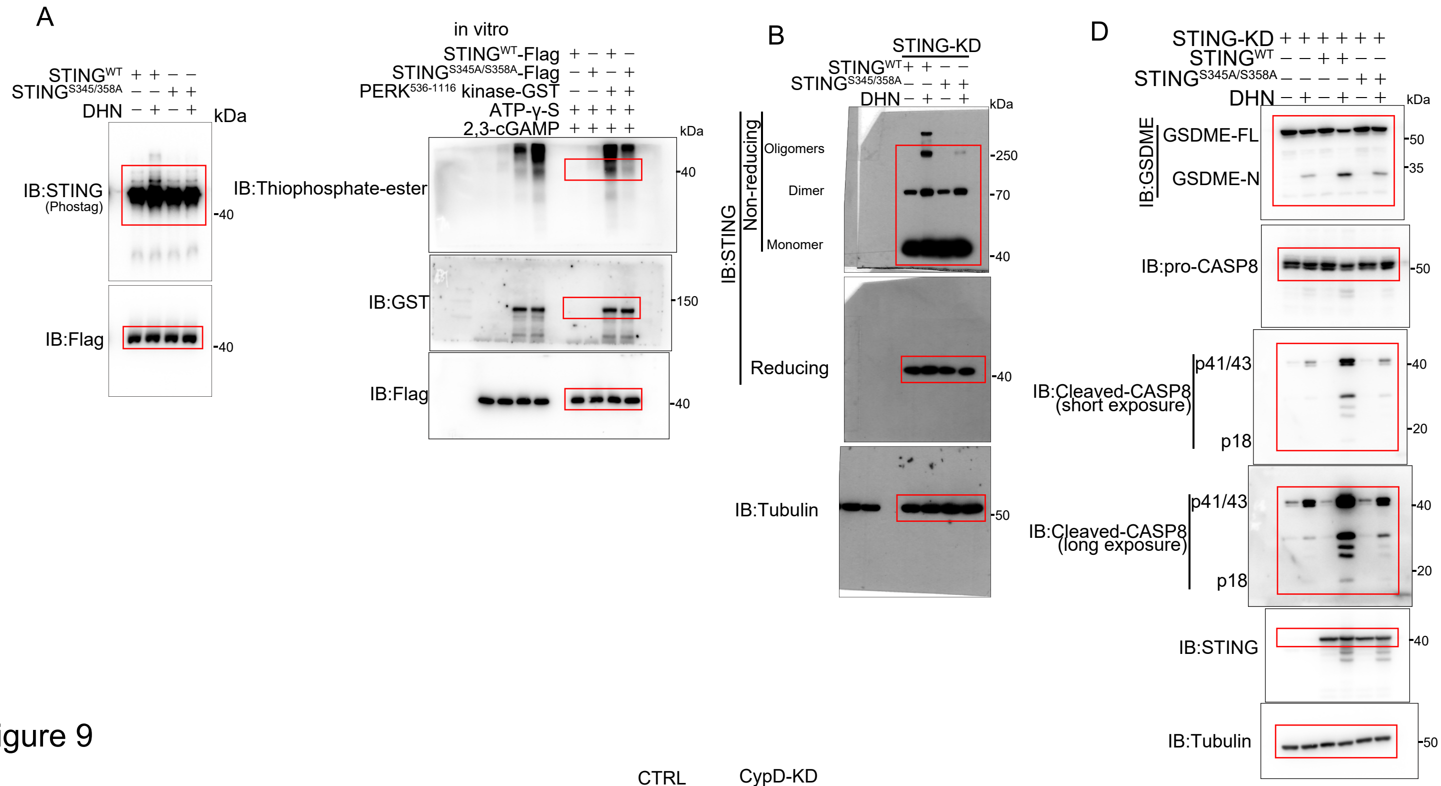

Figure 9

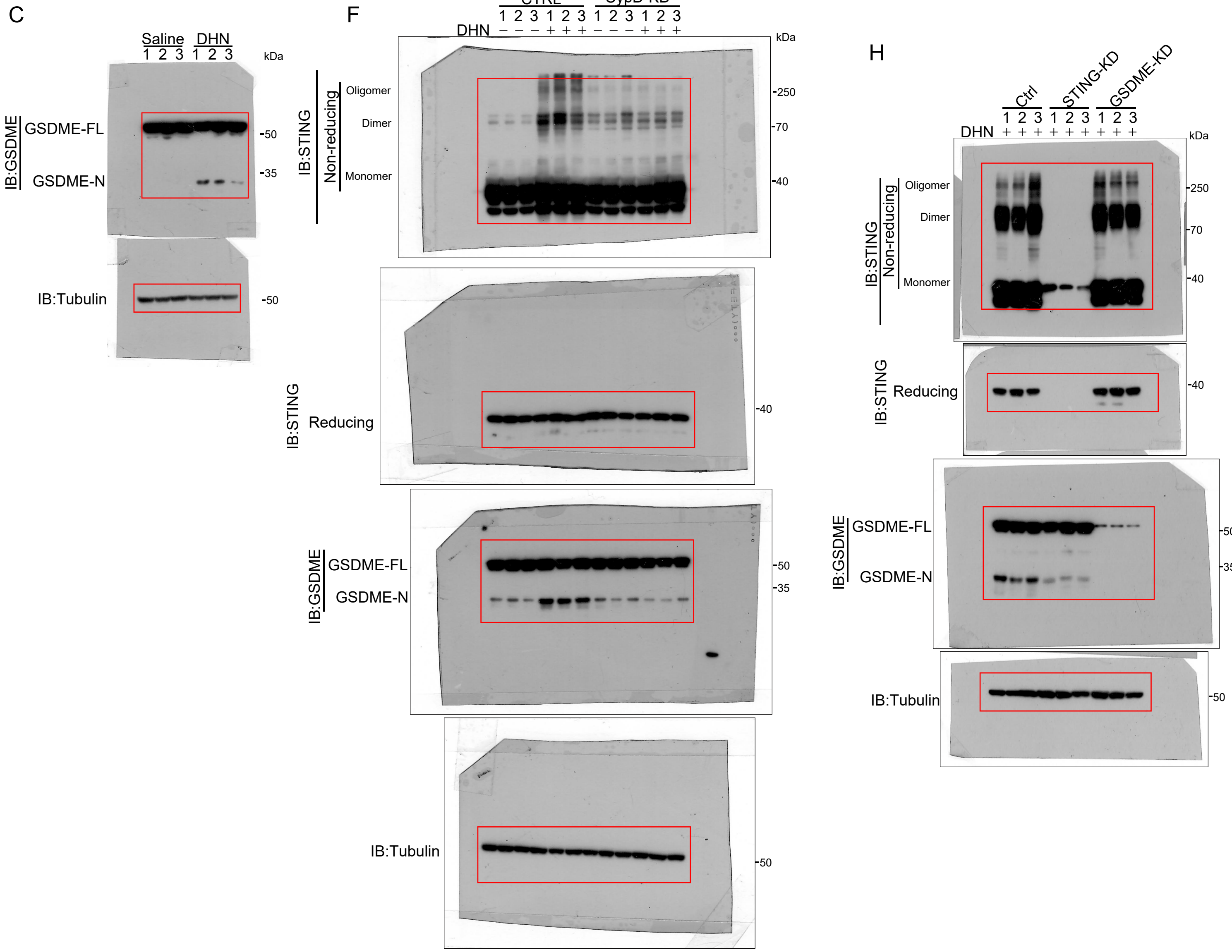

Figure 10

B

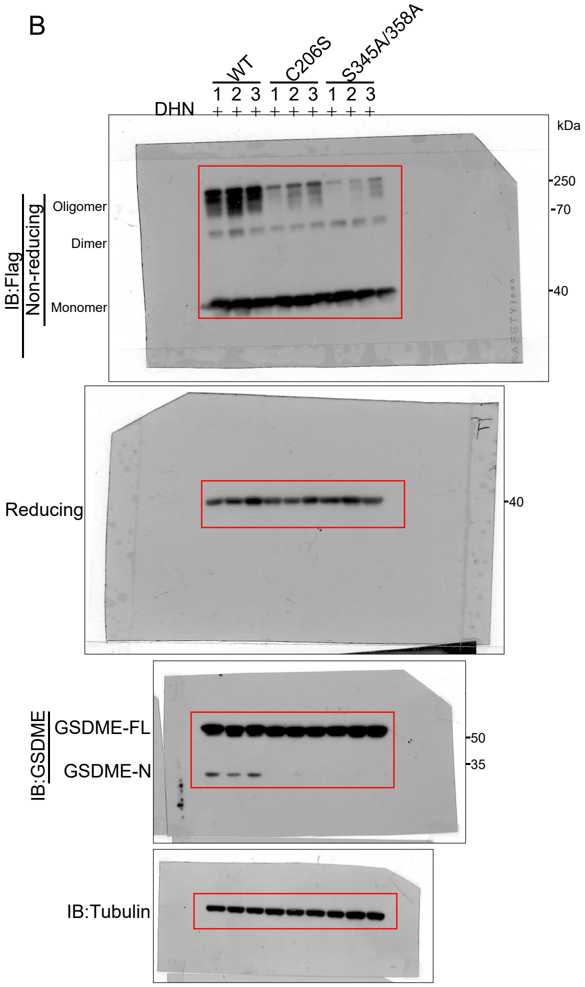
WT
C206S
S345A/358A
1 2 3 1 2 3 1 2 3
DHN
kDa
250
70
40
IB:Flag
Non-reducing
Oligomer
Dimer
Monomer
Reducing
40
IB:GSDME
GSDME-FL
GSDME-N
50
35
IB:Tubulin

C

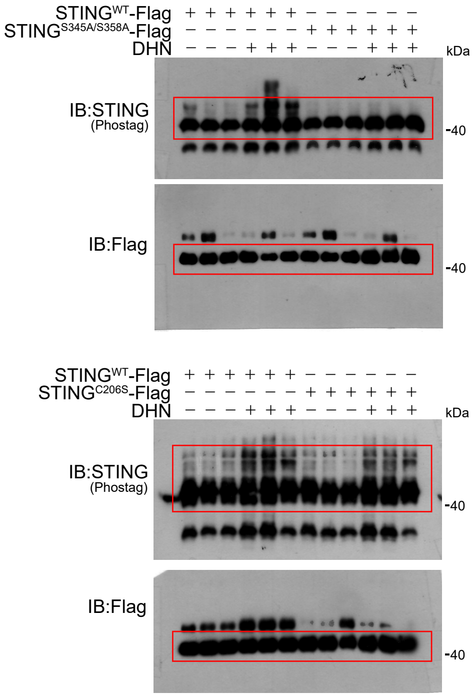
STING^{WT}-Flag
STING$^{S345A/S358A}$-Flag
DHN
kDa
IB:STING
(Phostag)
40
IB:Flag
40
STING^{WT}-Flag
STING^{C206S}-Flag
DHN
kDa
IB:STING
(Phostag)
40
IB:Flag
40

Supplementary Figure 1

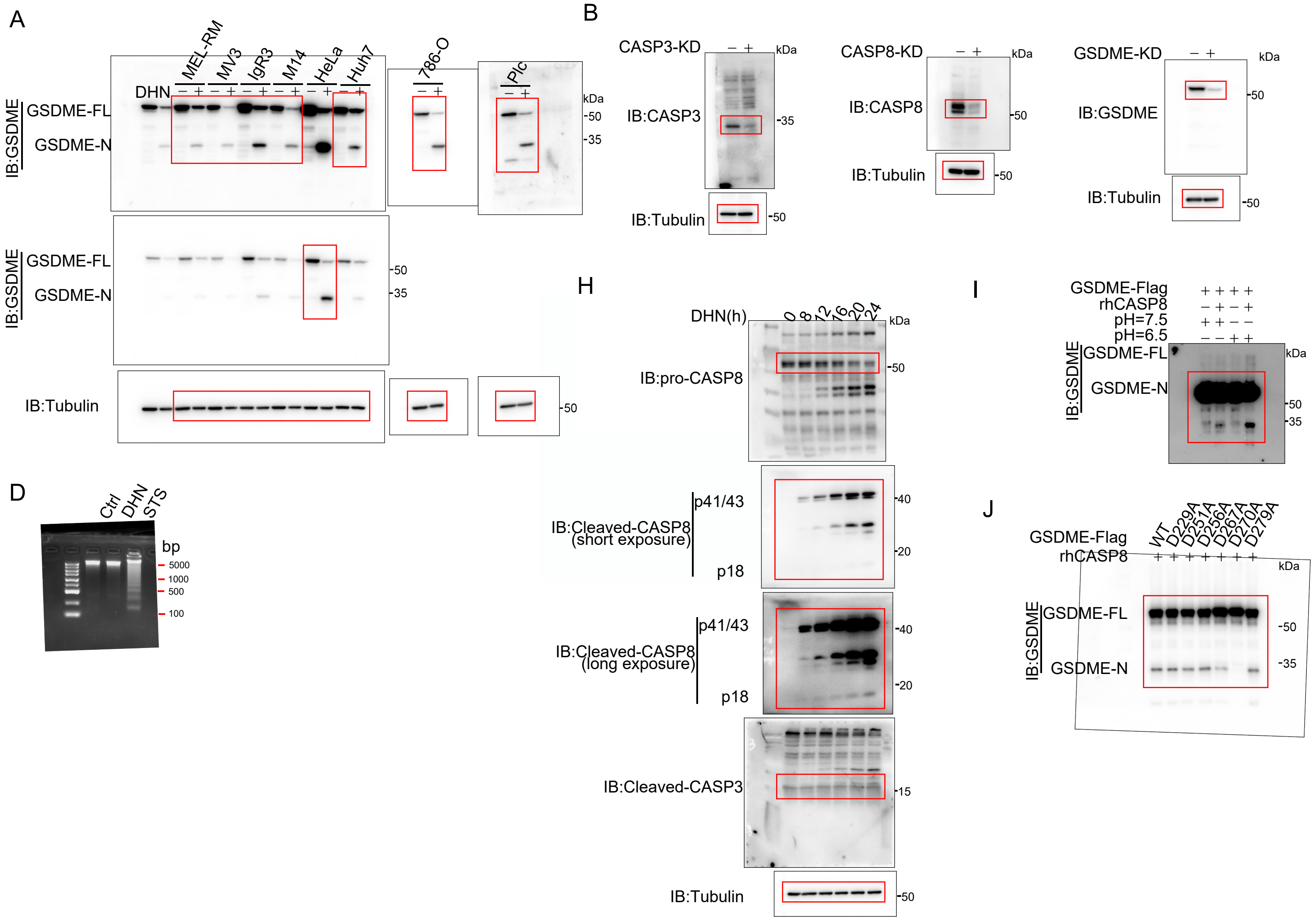

Supplementary Figure 2

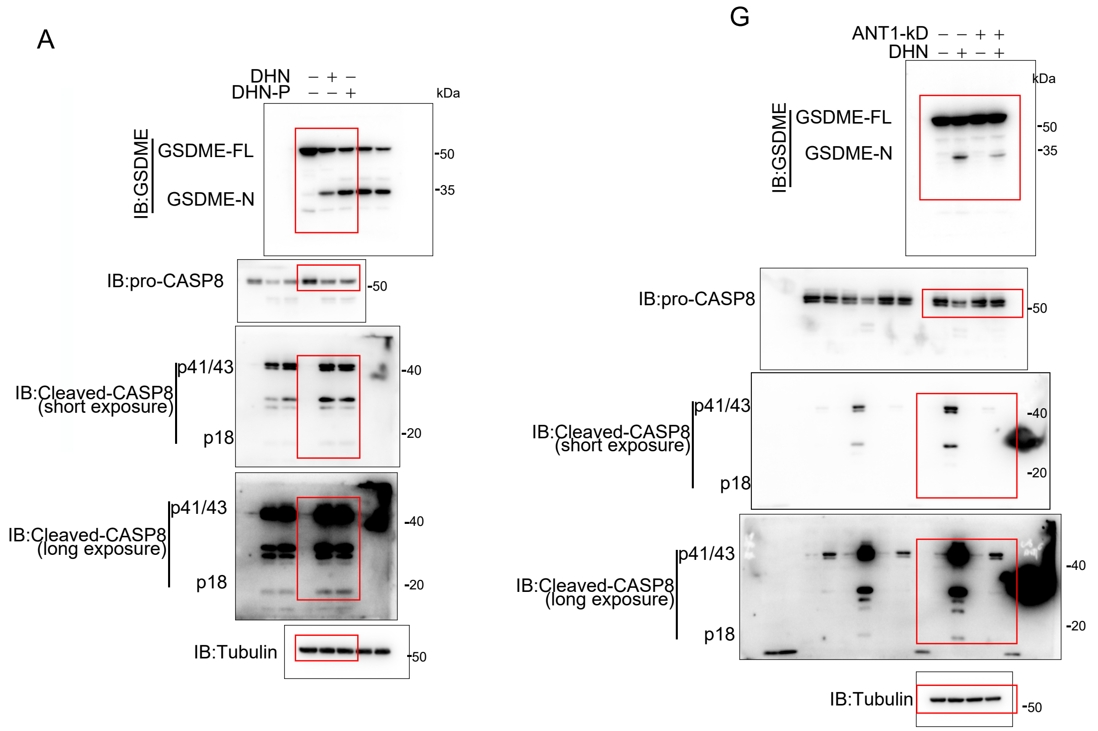

Supplementary Figure 3

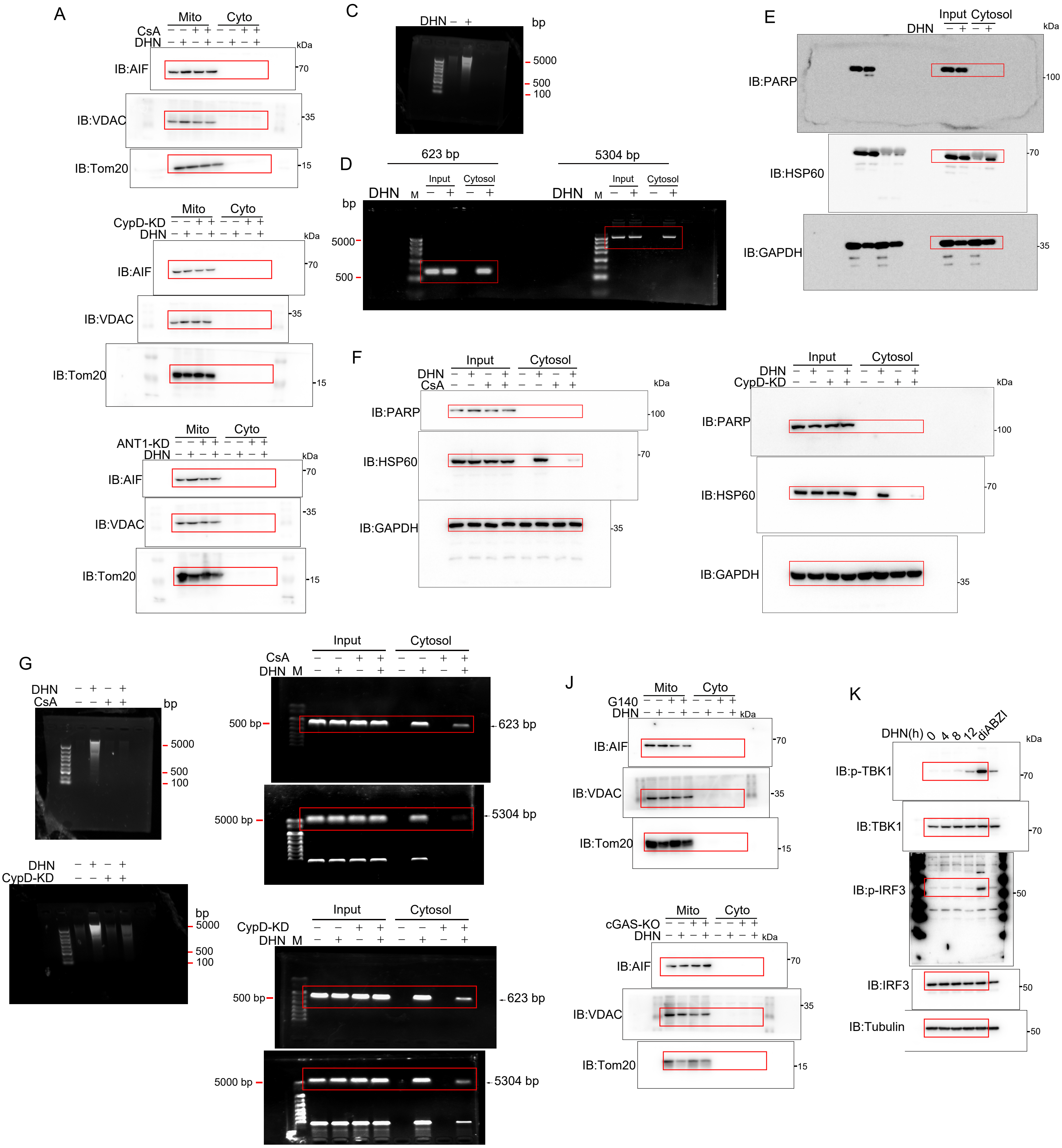

Supplementary Figure 4

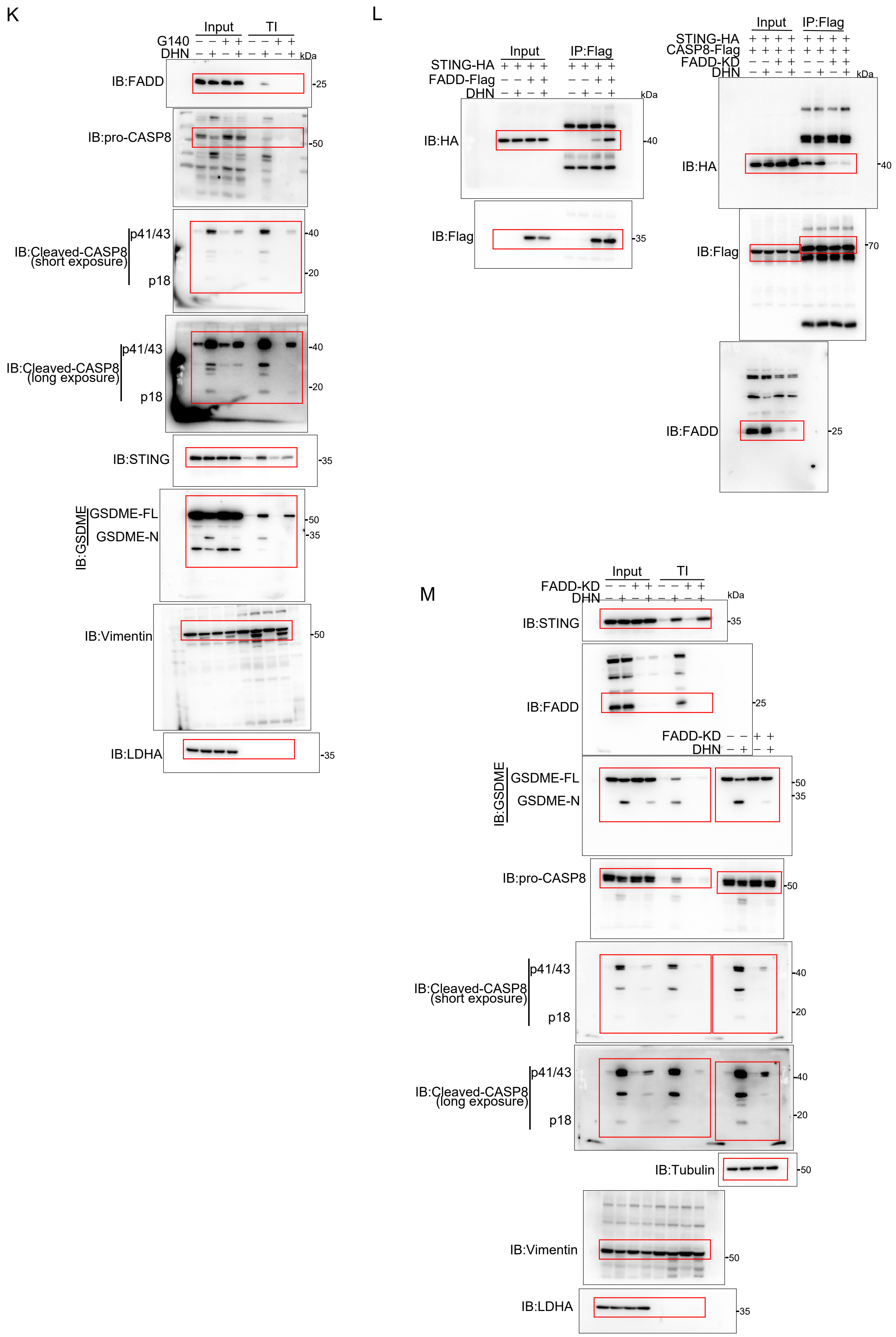

N

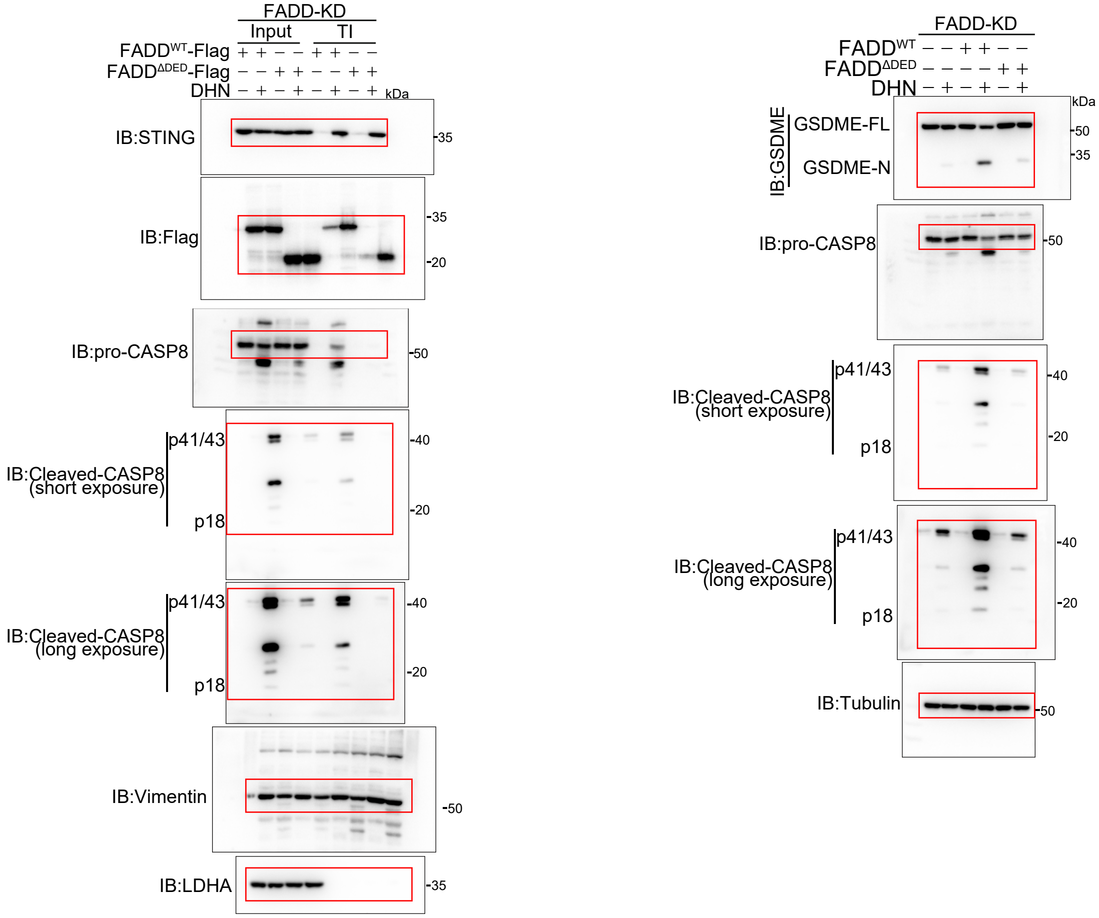
FADD-KD
Input
TI
FADDWT-Flag + + – – + + – –
FADDΔDED-Flag – – + + – – + +
DHN – + – + – + – +
kDa
IB:STING
35
IB:Flag
35
20
IB:pro-CASP8
50
IB:Cleaved-CASP8 (short exposure)
p41/43
p18
40
20
IB:Cleaved-CASP8 (long exposure)
p41/43
p18
40
20
IB:Vimentin
50
IB:LDHA
35
FADD-KD
FADDWT – – + + – –
FADDΔDED – – – – + +
DHN – + – + – +
kDa
IB:GSDME
GSDME-FL
GSDME-N
50
35
IB:pro-CASP8
50
IB:Cleaved-CASP8 (short exposure)
p41/43
p18
40
20
IB:Cleaved-CASP8 (long exposure)
p41/43
p18
40
20
IB:Tubulin
50

O

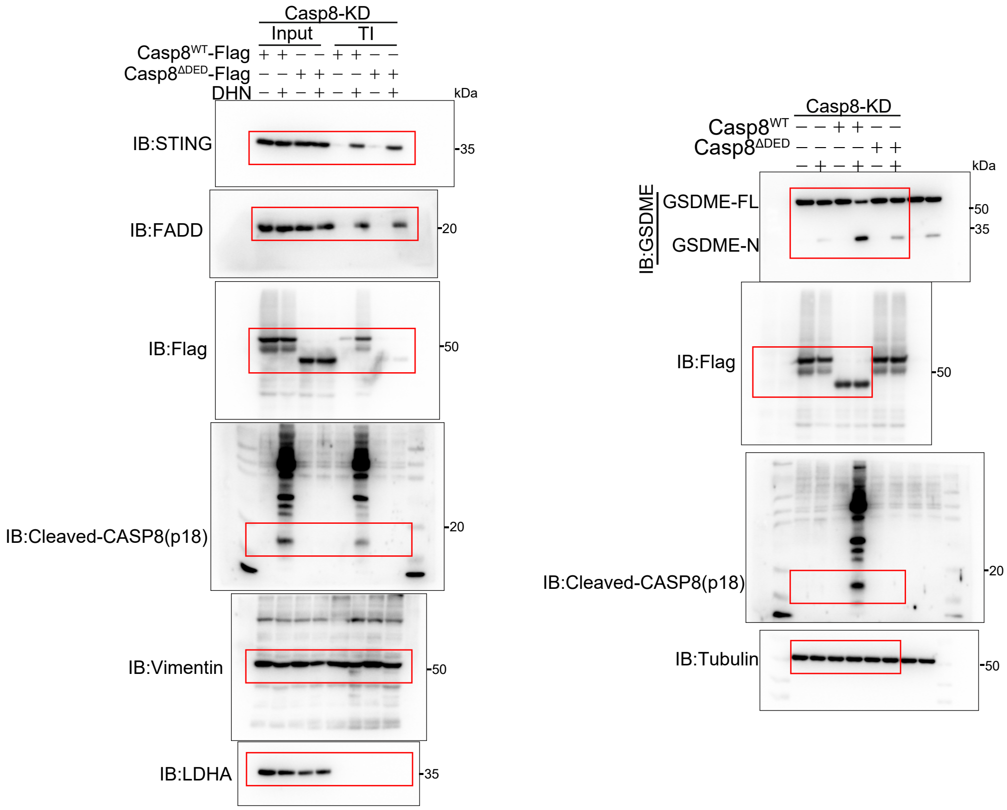
Casp8-KD
Input
TI
Casp8WT-Flag + + – – + + – –
Casp8ΔDED-Flag – – + + – – + +
DHN – + – + – + – +
kDa
IB:STING
35
IB:FADD
20
IB:Flag
50
IB:Cleaved-CASP8(p18)
20
IB:Vimentin
50
IB:LDHA
35
Casp8-KD
Casp8WT – – + + – –
Casp8ΔDED – – – – + +
– + – + – +
kDa
IB:GSDME
GSDME-FL
GSDME-N
50
35
IB:Flag
50
IB:Cleaved-CASP8(p18)
20
IB:Tubulin
50

Supplementary Figure 5

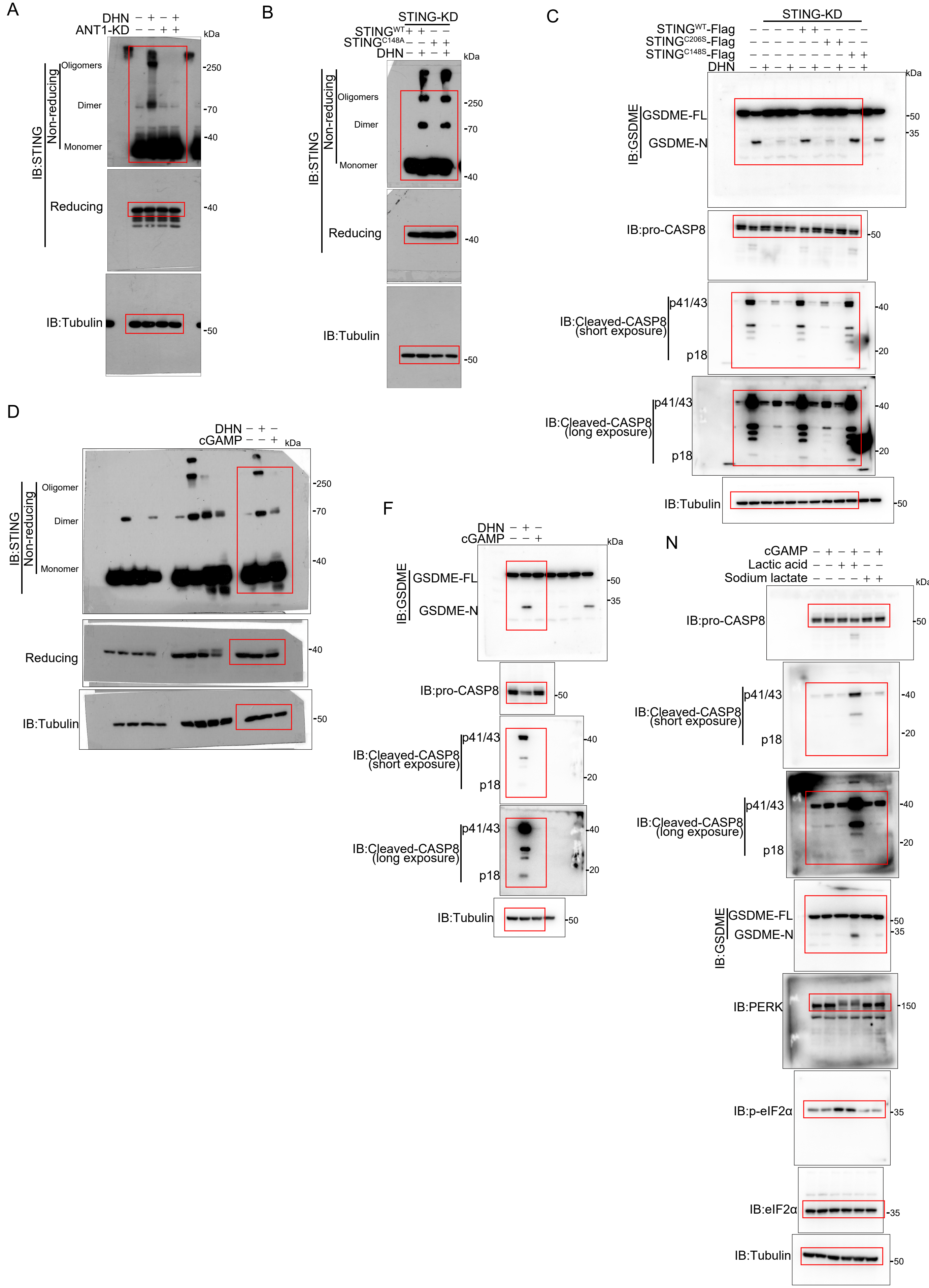

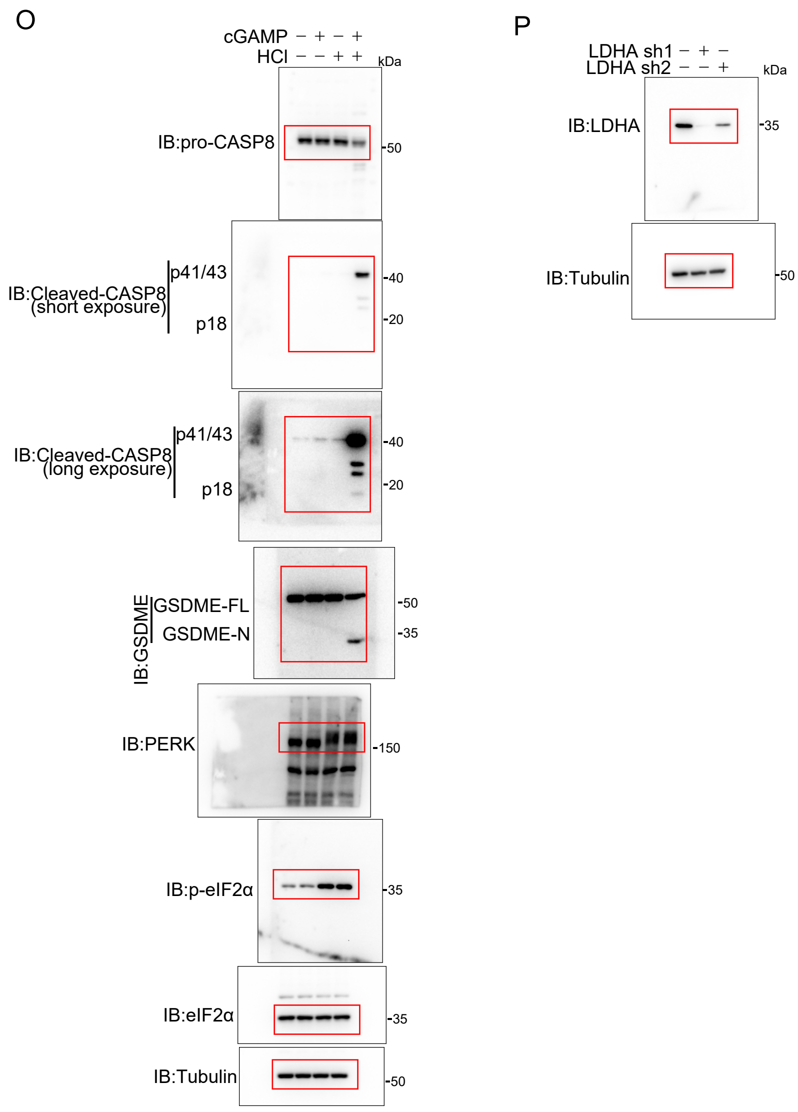

Supplementary Figure 6

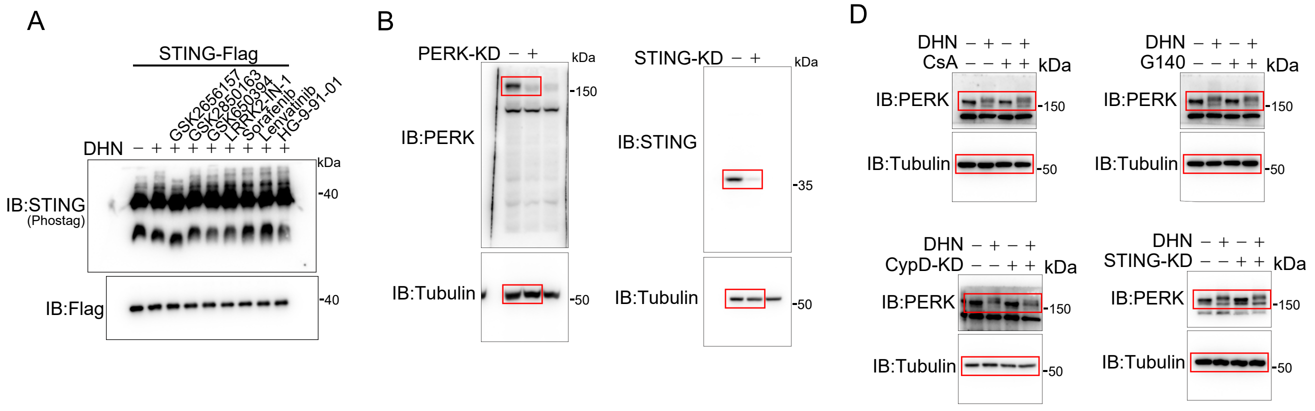

E

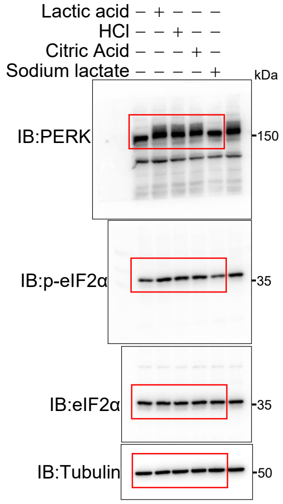
Lactic acid – + – – –
HCl – – + – –
Citric Acid – – – + –
Sodium lactate – – – – +
kDa
IB:PERK
150
IB:p-eIF2α
35
IB:eIF2α
35
IB:Tubulin
50

J

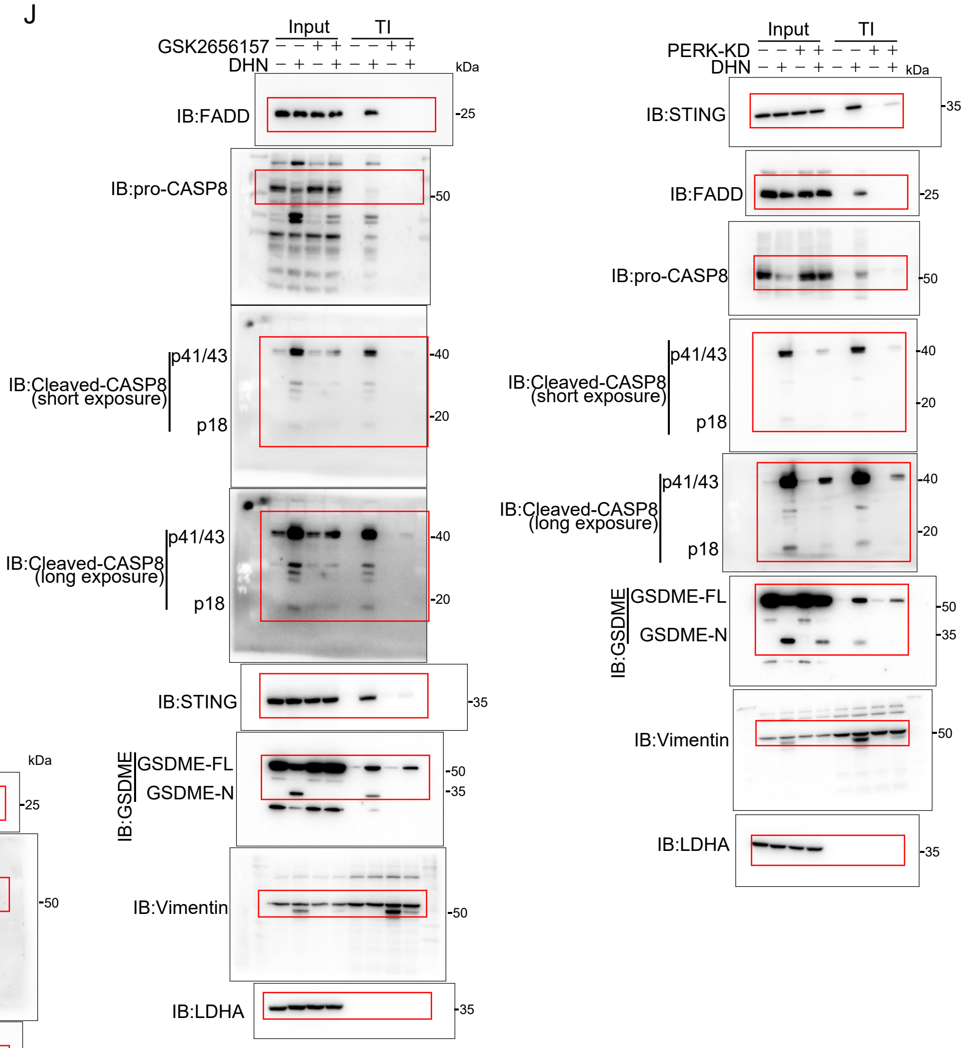
Input
TI
GSK2656157 – – + + – – + +
DHN – + – + – + – +
kDa
IB:FADD
25
IB:pro-CASP8
50
IB:Cleaved-CASP8 (short exposure)
p41/43
p18
40
20
IB:Cleaved-CASP8 (long exposure)
p41/43
p18
40
20
IB:STING
35
IB:GSDME
GSDME-FL
GSDME-N
50
35
IB:Vimentin
50
IB:LDHA
35
Input
TI
PERK-KD – – + + – – + +
DHN – + – + – + – +
kDa
IB:STING
35
IB:FADD
25
IB:pro-CASP8
50
IB:Cleaved-CASP8 (short exposure)
p41/43
p18
40
20
IB:Cleaved-CASP8 (long exposure)
p41/43
p18
40
20
IB:GSDME
GSDME-FL
GSDME-N
50
35
IB:Vimentin
50
IB:LDHA
35

K

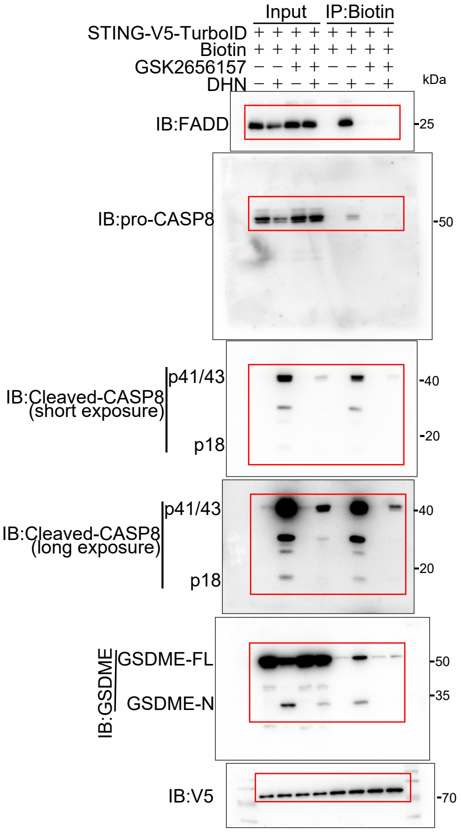
Input
IP:Biotin
STING-V5-TurboID + + + + + + + +
Biotin + + + + + + + +
GSK2656157 – – + + – – + +
DHN – + – + – + – +
kDa
IB:FADD
25
IB:pro-CASP8
50
IB:Cleaved-CASP8 (short exposure)
p41/43
p18
40
20
IB:Cleaved-CASP8 (long exposure)
p41/43
p18
40
20
IB:GSDME
GSDME-FL
GSDME-N
50
35
IB:V5
70

L

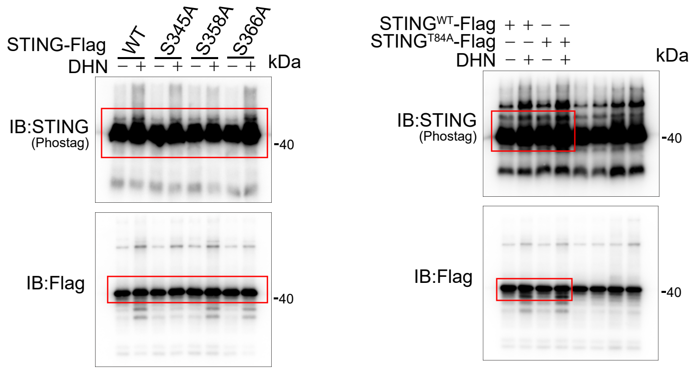
STING-Flag
WT
S345A
S358A
S366A
DHN – + – + – + – +
kDa
IB:STING (Phostag)
40
IB:Flag
40
STING^{WT}-Flag + + – –
STING^{T84A}-Flag – – + +
DHN – + – +
kDa
IB:STING (Phostag)
40
IB:Flag
40

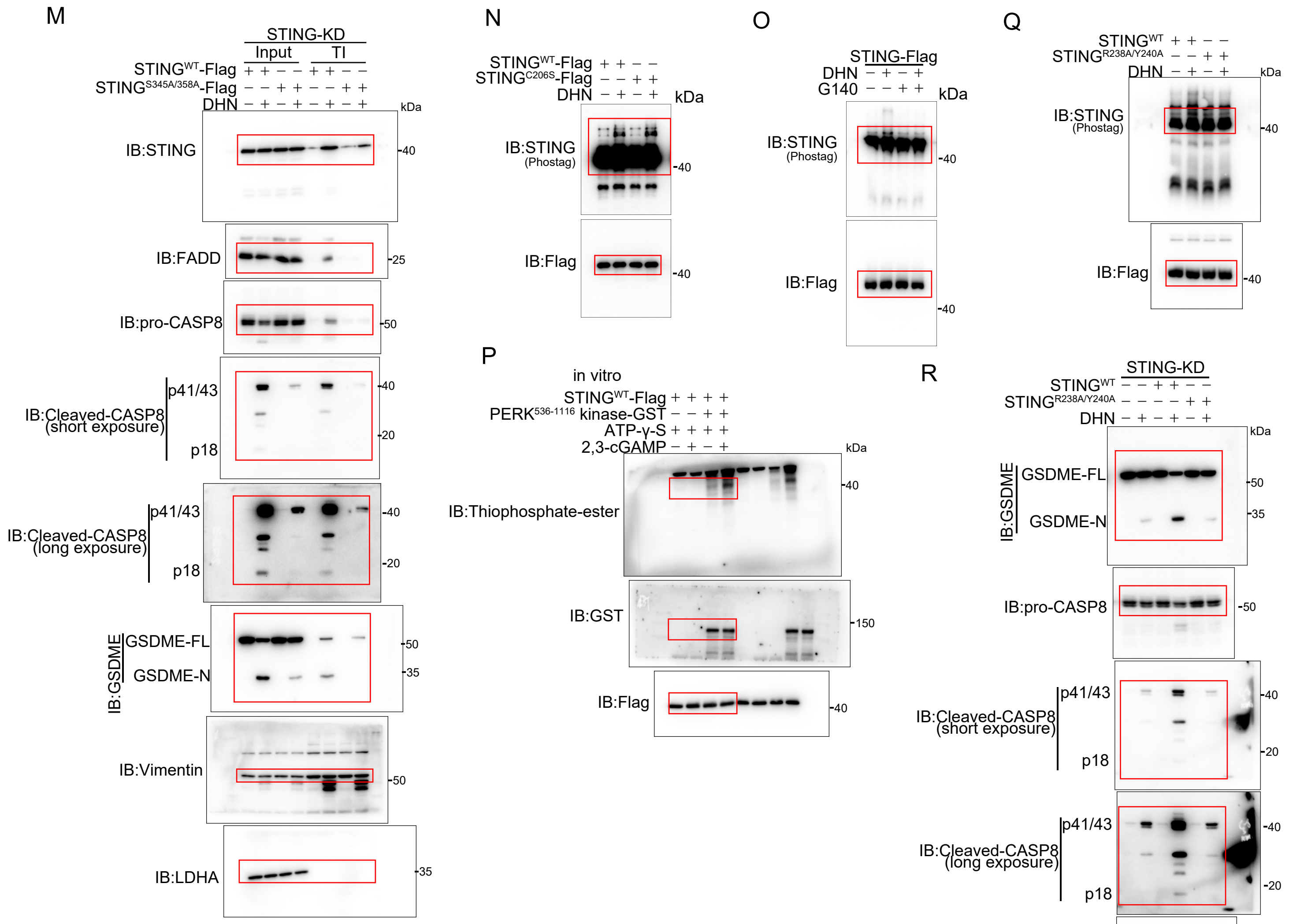

Supplementary Figure 7

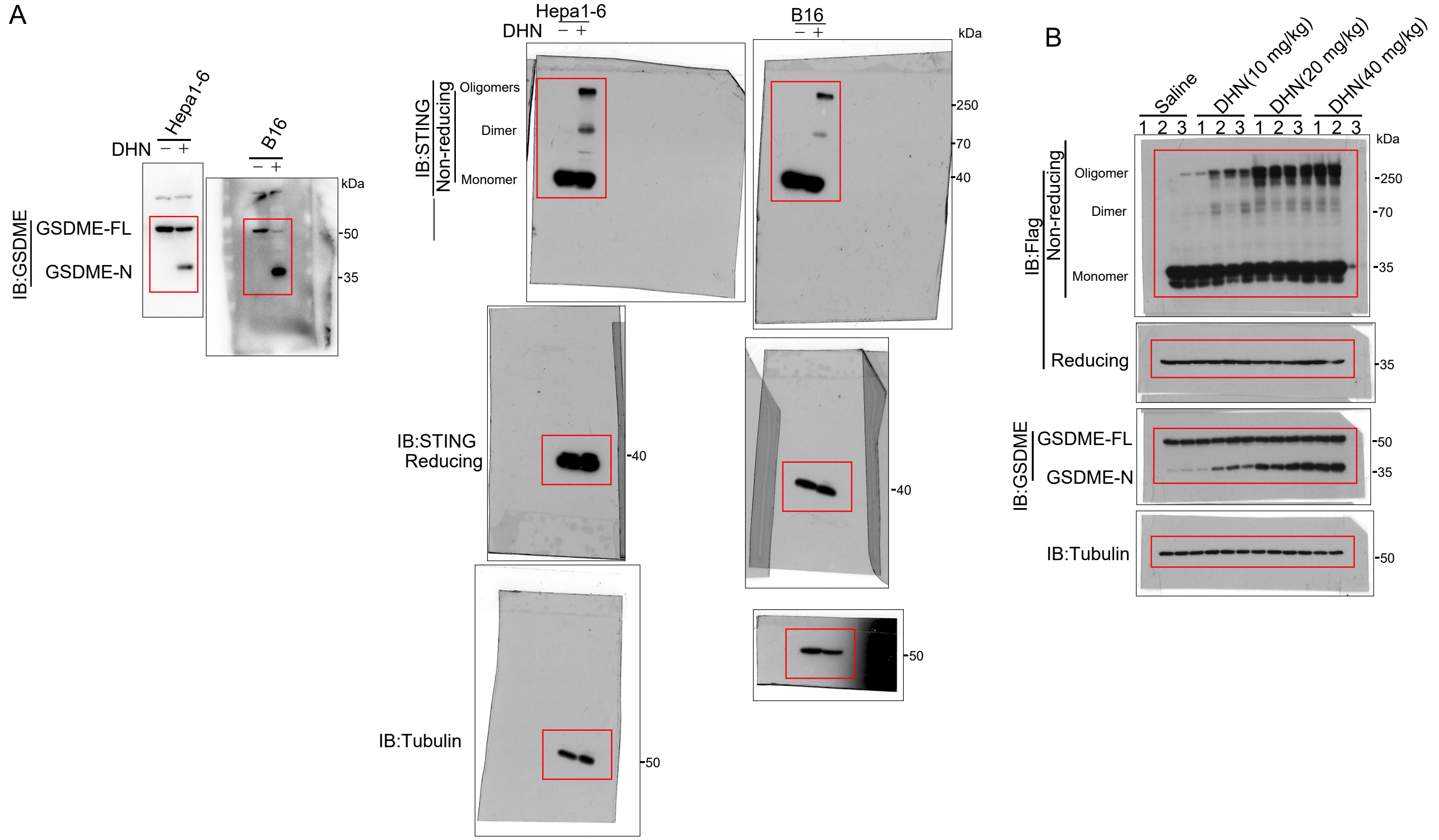

Supplement: Unedited blot and gel images [file jci-135-188872-s028.pdf]
